# Efficacy and safety of multiple treatments for small hepatocellular carcinoma: an updated systematic review and component network meta analysis

**DOI:** 10.1016/j.eclinm.2026.104037

**Published:** 2026-06-23

**Authors:** Peiyan Sun, Jianlin Wu, Dong Cai, Tianrun Lv, Yanwen Jin, Hailin Tang, Fuyu Li, Haijie Hu

**Affiliations:** a Division of Biliary Surgery, Department of General Surgery, West China Hospital, Sichuan University, Chengdu, Sichuan Province, China; b State Key Laboratory of Oncology in South China, Sun Yat-Sen University Cancer Center, Guangzhou, China

**Keywords:** Small hepatocellular carcinoma, Treatment, Disease-free survival, Treatment outcome, Network meta-analysis

## Abstract

**Background:**

This is an updated component network meta-analysis to evaluate efficacy and safety of various therapeutic approaches and their combinations for small hepatocellular carcinoma (SHCC) following PRISMA 2020 guideline.

**Methods:**

We screened two previously published meta-analyses on this topic, and searched PubMed, Embase, and the Cochrane Library from 1 January 2021 to 12 March 2026 for randomized controlled trials (RCTs) focusing on treatments of SHCC. We compared the efficacy and safety of surgical resection (SR), radiofrequency ablation (RFA), microwave ablation (MWA), transcatheter arterial chemoembolization (TACE), radiotherapy (RT), and their combinations. Outcomes were overall survival (OS), recurrence-free survival (RFS), and the rate of complications. Standard network meta-analysis (NMA), component NMA were performed, adding subgroup analysis, network meta-regression, and meta-regression between outcomes for sensitive analysis. This study is registered in PROSPERO (CRD420251132447).

**Findings:**

We included 41 RCTs involving 5804 patients. RFA + ^125^I [OS: Hazard ratio (HR) 0.50, 95% confidence interval (CI) 0.31–0.81; RFS: HR 0.51, 95% CI 0.32–0.81], TACE + RFA (OS: HR 0.66, 95% CI 0.52–0.85; RFS: HR 0.70, 95% CI 0.57–0.86), TACE + SR (OS: HR 0.30, 95% CI 0.16–0.58; RFS: HR 0.37, 95% CI 0.23–0.59) and SR (OS: HR 0.83, 95% CI 0.71–0.98; RFS: HR 0.82, 95% CI 0.71–0.94) had better OS and RFS than RFA; MWA had better RFS (HR 0.75, 95% CI 0.60–0.93), while TACE + RT might worsen RFS (HR 3.53, 95% CI 1.29–9.62). SR yielded more complications (RR 1.52, 95% CI 1.14–2.03). Better liver function (Child-Pugh A) was negatively correlated with HR of RFS (−0.16, 95% CI −0.28 to −0.04).

**Interpretation:**

SR remains the foundational treatment for SHCC, and TACE + SR exhibits superior efficacy in SHCC patients. RFA + ^125^I serves as a potential alternative in patients with unresectable tumors, and most locoregional treatments show effects comparable to SR for SHCC. Liver function may negatively affect relative efficacy of treatments.

**Funding:**

This study is funded by 1.3.5 project for disciplines of excellence-Clinical Research Incubation Project, West China Hospital, SCU; 1.3.5 project for Artificial Intelligence West China Hospital, SCU; National Natural Science Foundation of China for Young Scientists Fund; Sichuan Science and Technology Program; (Qilu) Clinical Research of Sichuan Anticancer Association.

**Research in context**

**Evidence before this study**

The most recent network meta-analysis focusing on small or early-stage hepatocellular carcinoma (SHCC) was published in 2023. Although it integrated 37 randomized controlled trials (RCTs) to facilitate pairwise comparisons, the authors noted that the comparative effectiveness rankings remained highly uncertain due to the suboptimal quality of several included RCTs. While the analysis suggested that surgical resection (SR) and ^125^I implantation combined with radiofrequency ablation (RFA) offer superior survival benefits over percutaneous ethanol injection (PEI) or single RFA, respectively. More importantly, 16 additional randomized controlled trials (RCTs) have been published, underscoring the need for an updated systematic review. Additionally, the European Association for the Study of the Liver released its 2025 guidelines on interventions for hepatocellular carcinoma, though no section specifically addresses early-stage hepatocellular carcinoma. Importantly, no prior systematic review has focused on the incremental effects of treatment combinations. We screened two previously published meta-analyses on this topic and searched PubMed, Embase, and the Cochrane Library from 1 January 2021 to 12 March 2026 for papers published in English using the terms “hepatocellular carcinoma” and “randomized controlled trials”, and screened for studies focusing on small hepatocellular carcinoma manually. Our search yielded 4468 results.

**Added value of this study**

To our knowledge, this study is the first to analyze the incremental effects of treatment components in combination therapies using component network meta-analysis, and provides evidence of moderate-to-high levels on treatment options specifically for SHCC. By analyzing 41 RCTs involving 5804 patients, we demonstrate that surgical resection remains good in treating SHCC, though associated with increased number of complications. Locoregional treatments show promising effects compared to SR, with MWA being slightly more effective than other locoregional treatments. Combination treatments, including ^125^I-implantation + radiofrequency ablation (RFA), transcatheter arterial chemoembolization (TACE) + RFA, and TACE + SR, show better outcomes than single RFA. TACE + RFA/SR showed favorable survival outcomes and suggested potential synergistic signals in the CNMA framework, while TACE + radiotherapy is not favored. No significant difference was observed in subgroup analysis distinguishing tumor size, while liver function (estimated as the proportion of patients with Child–Pugh class A liver function in each study) was negatively associated with the hazard ratio for recurrence-free survival in network meta-regression.

**Implications of all the available evidence**

This study provides moderate to high levels of evidence for the treatment of SHCC. We highlight the role of locoregional treatments (ablations, RT, etc.) for SHCC in achieving effects comparable to surgery, and provide guidance for specialists in selecting appropriate treatments. For patients with significant risk factors (e.g., poor liver function), combination therapies are recommended to prevent recurrence. Additionally, TACE-containing combinations, particularly TACE + RFA and TACE + SR, showed favorable survival outcomes and potential incremental benefits, whereas the combination of TACE and RT should be interpreted cautiously.

## Introduction

Small hepatocellular carcinoma (SHCC) is generally defined as Barcelona Clinic Liver Cancer (BCLC) stage 0-A HCC with a maximum tumor diameter <3 cm.^1^ Nevertheless, the size threshold used to define clinically relevant early-stage HCC varies across studies and treatment guidelines, and recent treatment frameworks have increasingly emphasized tumor burden, liver function, vascular invasion, extrahepatic spread, and eligibility for curative-intent locoregional therapy, rather than tumor diameter alone.^2^ Considering its limited cancer infiltration and low rate of metastasis or vascular invasion, patients with SHCC may be eligible for curative-intent and minimally invasive treatments.^3^ Timely and optimal early therapeutic strategies specifically for SHCC are needed, which may significantly improve survival outcomes.

Surgical resection remains standard care for radical treatment, according to European Association for the Study of the Liver^1^ and American Association for the Study of Liver Diseases.^4^ However, patients differ in tumor locations and baseline liver functions, necessitating multiple therapies suited for patients in different medical conditions. Alternative locoregional therapies, including ablation, percutaneous ethanol injection (PEI), radiotherapy and transcatheter arterial chemoembolization (TACE), have been widely applied for SHCC since early 2000s.^1^ Radiofrequency ablation (RFA) is now recommended as first-line treatment for SHCC, and other techniques, alone or in combination, also achieve favorable local control in clinical practice.^5^, ^6^, ^7^, ^8^, ^9^, ^10^

These therapies could preserve more healthy liver than surgical resection, but their performance for radical treatment remains controversial. Several meta-analyses have compared these modalities,^11^ but most included non-randomized studies and failed to draw a validated conclusion. Since the latest publication in 2021^11^ and 2023,^12^ 16 additional randomized controlled trials (RCTs) have been reported. Moreover, the efficacy of combining therapies remains unclear. Component network meta-analysis (CNMA) is a novel approach that decomposes combination therapies into individual components under an additivity assumption and estimates their incremental effects to explore potential synergistic or antagonistic interactions.^13^

This study provided updated and upgraded evidence to compare the efficacy and safety of different treatments for SHCC. Additionally, we calculated the incremental effects of the treatments to explore the possibly synergistic or antagonistic interactions between the treatment components.

## Methods

### Search strategy, selection criteria, and risk of bias assessment

This study followed the Preferred Reporting Items for Systematic reviews and Meta-Analyses (PRISMA) extension for network meta-analysis, and the protocol was registered in PROSPERO (CRD420251132447). We screened two previously published meta-analyses on this topic,^11^^,^^12^ and searched PubMed, Embase, and the Cochrane Library from 1 January 2021 to 12 March 2026 using search strategy provided in Appendix 1, and the reference list of both included articles and previously published meta-analyses were screened. Duplicate records were removed automatically by Endnote 20®.

The inclusion criteria included: (1) Randomized controlled trials (RCTs) with at least two arms for comparison and with full-text published; (2) Adults (≥18 years) diagnosed with SHCC; (3) No other severe comorbidities or advanced liver disease (e.g., Child-Pugh C liver dysfunction); (4) At least one of the clinical outcomes, including overall survival (OS), progression-free survival (PFS), recurrence rate, and adverse events, is reported. The exclusion criteria included: (1) Non-randomized controlled trials (non-RCTs), animal studies or *in vitro* studies, or unpublished studies, abstracts, conference proceedings; (2) Studies involving patients with liver cancer of non-HCC origin (e.g., metastatic liver cancer, cholangiocarcinoma); (3) Studies that do not clearly specify patients with SHCC; (4) Studies involving pediatric patients (under 18 years of age); (5) Single-arm studies; (6) Non-English language studies. Two reviewers (Peiyan Sun and Jianlin Wu) independently selected potentially relevant studies based on the title and abstract, and full-text articles were then gathered and assessed. When an author or the same medical center has published two publications on the same topic, we checked the registration platform (if available) to make sure that different trials were included. Any discrepancy was checked by another independent reviewer (Fuyu Li).

For each RCT, two reviewers (Peiyan Sun and Jianlin Wu) independently assessed the risk of bias using the Risk of bias (RoB) 2.0 tool.^14^ Any discrepancy was resolved through discussion with a third independent reviewer (Fuyu Li). The risk of bias for each study was evaluated across five domains and rated as “low”, “some concerns”, or “high”. The overall risk of bias judgment was derived according to the algorithm recommended in the RoB 2.0 guideline.

### Outcomes and data extraction

The primary outcomes include overall survival (OS) and recurrence-free survival (RFS), estimated by hazard ratios (HRs) and corresponding 95% confidence intervals (95% CIs). For harmonization across studies, we adopted a broad definition of RFS as the time from treatment completion to the first occurrence of tumor recurrence, progression, metastasis, or death; endpoints reported as RFS, disease-free survival (DFS), or progression-free survival (PFS) in the original articles were all treated as RFS. The secondary outcome was the occurrence rate of overall complications, defined in line with CTCAE v5.0^15^ or Clavien-Dindo system,^16^ and risk ratios (RRs) with corresponding 95% confidence intervals (CIs) were calculated as the effect measures for this outcome.

Two reviewers (Peiyan Sun and Jianlin Wu) independently extracted data by using a prespecified form using Microsoft Excel 365, and any discrepancy was solved by another independent reviewer (Fuyu Li). For the primary outcomes (OS and RFS), we primarily extracted the reported hazard ratios (HRs) and corresponding 95% confidence intervals (95% CIs). When HRs and corresponding 95% CIs were not reported, they were estimated using data digitized from the Kaplan–Meier curves with Engauge Digitizer (version 11.1) following the method of Tierney et al.^17^ For the secondary outcome (complications), the numbers of complications and the corresponding numbers of affected patients were extracted from each included study. Additionally, we also collected demographic characteristics of each study including (1) the maximal tumor size (cm); (2) the percentage of patients with Child–Pugh class A liver function.

### Statistical analysis

We used different models in this study to estimate the pooled effect sizes of the outcomes. We first used standard network meta-analysis (standard NMA) to calculate the pooled effect sizes and heterogeneity. We then used CNMA to calculate the pooled effect sizes. This model was based on the hypothesis that the therapeutic or adverse effects of the treatment combination were additive, reflecting the sum of the individual component effects. Additionally, the incremental effects (iHR, iRR) of each treatment component and the existing combinations were calculated to detect the synergistic or antagonistic effects in the combinations. We then utilized subgroup analyses and network meta-regression as sensitivity analyses. The studies were categorized into two groups based on the maximum tumor size, using 3 cm as the cutoff value. Furthermore, the proportion of patients with Child–Pugh class A liver function was included as a covariate in the network meta-regression model to estimate the effect sizes. Forest plots, surface under the cumulative ranking curve (SUCRA) plots, and league tables were used to present the results. Heterogeneity was quantified using Cochran’s *Q* and I^2^, whereas inconsistency was explored visually using heat maps and assessed quantitatively within the network framework. The transitivity assumption was assessed clinically by comparing the distribution of key effect modifiers across treatment comparisons, particularly maximum tumor size and the proportion of Child–Pugh class A patients. Publication bias was detected using funnel plots and Egger’s test. Moreover, we performed a meta regression between OS, RFS and complications respectively, and the results were presented in bubble plots. Given the relatively sparse network structure and the limited number of studies informing several treatment comparisons, all primary analyses were performed using a fixed-effect model, and we further displayed standard NMA in random-effect model as sensitivity analysis.

All the statistical analyses were performed using R (version 4.3.1).^18^ Standard NMA, CNMA, and subgroup analyses were performed by “netmeta” package (version 3.2-0),^19^ and meta-regressions were performed by “gemtc” package (version 1.1-0)^20^ and “mixmeta” package (version 1.2.0).^21^ The level of statistical significance was set at two-sided 5%. All the protocols of statistical analysis were prespecified and registered in PROSPERO. All authors had access to the study data. They reviewed and approved the final manuscript.

### GRADE certainty assessment

The certainty of evidence was assessed using the Grading of Recommendations Assessment, Development, and Evaluation (GRADE) approach. The evidence quality was classified into four levels: high, moderate, low, and very low, based on the consideration of six domains, within-study bias, reporting bias, indirectness, imprecision, heterogeneity, and inconsistency. For each outcome, the certainty was initially rated as high for RCTs and could be downgraded by one or two levels according to the presence and severity of limitations in each domain. The GRADE assessment was performed using Confidence in Network Meta-Analysis (CINeMA).^22^^,^^23^ For the assessment of imprecision, we prespecified the minimal clinically important difference (MCID) according to the approach proposed by Horita et al.; specifically, a small effect threshold of Cohen’s *d* = 0.2 was adopted and converted to relative effect thresholds of 1.20 or 0.83 for HRs/RRs. Comparisons with confidence intervals crossing these thresholds were judged as imprecise in the GRADE/CINeMA assessment. A table was generated to list the rating of each head-to-head comparison.

### Ethics statement

Current study was performed based on previously published studies. The ethical approval and informed consent were not required.

### Role of the funding source

The funder of the study had no role in study design, data collection, data analysis, data interpretation, or writing of the report. All authors had full access to the data in the study. Peiyan Sun, Jianlin Wu and Dong Cai had final responsibility for the decision to submit the manuscript for publication.

## Results

### Baseline characteristics of the studies

**Fig. 1:**
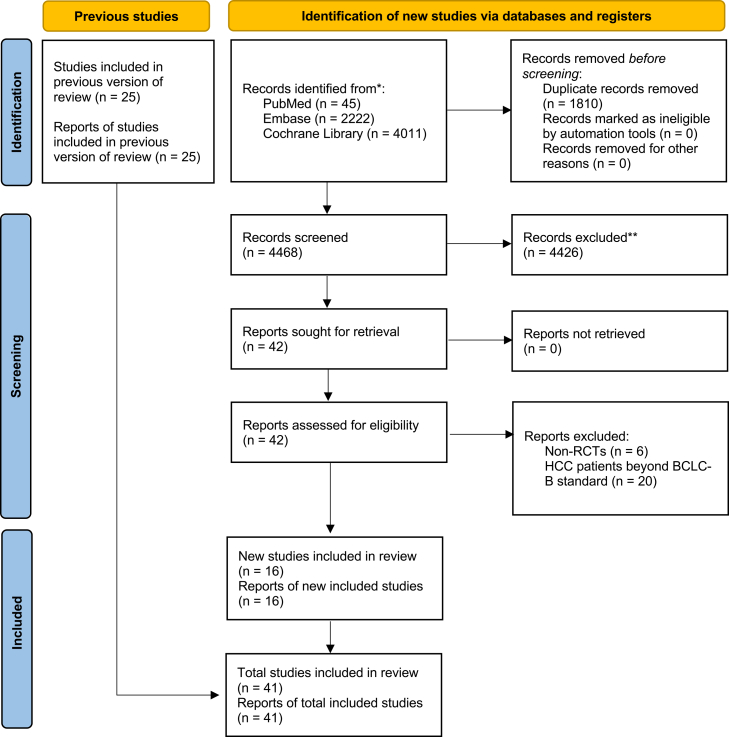
**PRISMA 2020 flow diagram for the screening of published RCTs.** The process of screening studies. HCC: hepatocellular carcinoma.

A total of 41 RCTs involving 5804 patients were finally included for meta-analysis. The processes of retrieval, screening, and inclusion are illustrated in Fig. 1, and the included RCTs were listed in the Supplementary material. Baseline characteristics of the studies were listed in Table 1. The treatment modalities evaluated included cryoablation (CA), irreversible electroporation (IRE), laser ablation (LA), microwave ablation (MWA), percutaneous ethanol injection (PEI), radiofrequency ablation (RFA), no-touch radiofrequency ablation (NT-RFA), RFA combined with percutaneous iodine-125 seed implantation (RFA + ^125^I), radiotherapy (RT), surgical resection (SR), transcatheter arterial chemoembolization (TACE), drug-eluting microsphere transcatheter arterial chemoembolization (DEM-TACE), and several combination therapies (including PEI + TACE, TACE + RFA, TACE + RT, and TACE + SR). Ten studies included patients with tumors no greater than 3 cm, while other 31 studies included patients with tumors greater than 3 cm. Notably, one study divided patients into two subgroups according to whether the largest tumor diameter was ≤3 cm or >3 cm, and reported OS and RFS separately.^24^ Accordingly, this study was considered as two independent datasets in the analyses of OS and RFS, but as a single study in the analysis of complications. The proportion of Child–Pugh class A patients ranged from 37.8% to 100%. No obvious violation of transitivity was observed based on available covariates. Considering the risk of bias, thirty-one studies (75.61%) were judged to have a low risk of bias, six studies (14.63%) had some concerns regarding risk of bias, and four studies (9.76%) were assessed as having a high risk of bias. Details of the RoB 2.0 assessment are provided in Supplementary Fig. S3 and Appendix 3 of the Supplementary material. The most common sources of bias were the lack of a prespecified protocol and trial registration, and a high proportion of patients lost to follow-up.

**Table 1:** Baseline characteristics and outcomes of the included studies.

Authors	Year	Country	Maximal tumor size (cm)	Treatments	Sample size	Age (mean ± SD or median, range)	Gender (M/F)	Child-Pugh (A/B)	OS (HR, 95%CI)^a^	RFS (HR, 95%CI)^a^	Complications (RR, 95%CI)^b^
Abdelaziz et al.	2014	Egypt	5	MWA	66	53.6 ± 5	48/18	25/41	0.62 (0.24, 1.59)	NA	0.57 (0.12, 2.80)
				RFA	45	56.8 ± 7.3	31/14	24/21			
Brunello et al.	2008	Italy	3	RFA	70	69 ± 7.7	43/27	39/31	0.82 (0.48, 1.41)	NA	0.92 (0.42, 1.98)
				PEI	69	70.3 ± 8.1	49/20	39/30			
Bush et al.	2023	USA	6.5	RT	35	61.7 ± 8.51	27/8	15/20^c^	0.88 (0.46, 1.67)	0.28 (0.12, 0.62)	NA
				TACE	39	59.6 ± 9.15	26/13	13/26^c^			
Chen et al.^d^	2006	China	3	RFA	37	51.9 ± 11.2	56/15^e^	NA	1.04 (0.30, 3.62)	1.81 (0.91, 3.64)	0.29 (0.1, 0.91)^e^
				SR	42	49.4 ± 10.9	75/15^e^	NA			
			6	RFA	34	51.9 ± 11.2		NA	0.86 (0.45, 1.65)	0.69 (0.38, 1.25)	
				SR	48	49.4 ± 10.9		NA			
Chen et al.	2014	China	3	RFA+^125^I	68	50.79 ± 6.79	56/12	NA	0.50 (0.31, 0.81)	0.51 (0.32, 0.82)	NA
				RFA	68	48.91 ± 7.30	53/15	NA			
Chong et al.	2020	China	5	MWA	47	63.0 (50.0, 80.0)	30/17	39/7	0.79 (0.54, 1.15)	NA	0.99 (0.06, 15.37)
				RFA	46	64.5 (42.0, 85.0)	38/8	40/6			
Costanzo et al.	2015	Italy	5	RFA	70	NA	53/17	63/7	1.51 (0.86, 2.63)	0.96 (0.49, 1.88)	0.88 (0.53, 1.47)
				LA	70	NA	47/23	67/3			
Fang et al.	2014	China	4	SR	60	53.5 ± 11.0	46/14	43/17	0.89 (0.61, 1.30)	0.94 (0.44, 2.02)	2.53 (0.61, 10.49)
				RFA	60	51.3 ± 8.1	42/18	32/23			
Fang et al.	2023	China	NA	TACE + SR	82	NA	68/14	82/0	0.36 (0.19, 0.68)	0.45 (0.29, 0.71)	NA
				SR	82	NA	73/9	82/0			
Feng et al.	2012	China	4	SR	84	47 (18, 76)	75/9	43/41	0.77 (0.37, 1.61)	0.59 (0.34, 1.03)	1.42 (0.65, 3.09)
				RFA	84	51 (24, 83)	79/5	39/45			
Féray et al.	2023	France	9	TACE	64	71.5 ± 9.4	55/9	57/7	1.23 (0.83, 1.82)	0.78 (0.50, 1.20)	NA
				TACE + RT	56	69.2 ± 8.8	48/8	48/8			
Ferrari et al.	2007	Italy	4	RFA	40	70.53 ± 5.3	28/12	28/12	0.77 (0.13, 4.45)	NA	NA
				LA	41	68.27 ± 6.4	28/13	31/10			
Giorgio et al.	2011	Italy	3	PEI	143	72 ± 6	102/41	75/68	1.23 (0.72, 2.17)	NA	1.35 (0.12, 14.69)
				RFA	142	70 ± 2	105/37	70/72			
Gjoreski et al.	2021	North Macedonia	NA	TACE	28	67.93 ± 7.40	20/8	15/12	1.00 (0.95, 1.05)	NA	NA
				DEM-TACE	32	68.81 ± 7.0	21/11	9/21			
Huang et al.	2005	China	3	PEI	38	63 ± 10.9	19/19	35/3	4.62 (1.58, 13.52)	1.38 (0.77, 2.48)	2.33 (0.12, 43.65)
				SR	38	59 ± 11.4	27/11	38/0			
Huang et al.	2010	China	5	RFA	115	56.57 ± 14.3	79/36	110/5	2.29 (1.12, 4.68)	1.55 (1.05, 2.30)	NA
				SR	115	55.91 ± 12.68	85/30	106/9			
Kamal et al.	2019	Egypt	3	MWA	28	55 (42, 80)	21/7	22/6	NA	1.39 (0.18, 10.55)	2.01 (0.10, 40.07)
				RFA	28	55 (42, 80)	22/6	22/6			
Koda et al.	2001	Japan	3	TACE + PEI	26	66.2 ± 8.0	14/12	19/5	0.74 (0.29, 1.89)	0.48 (0.23, 1.00)	2.01 (0.10, 39.94)
				PEI	26	66.4 ± 6.6	18/8	14/8			
Lee et al.	2018	Korea	4	SR	29	55.6 ± 7.9	23/6	29/0	0.85 (0.33, 2.20)	0.48 (0.22, 1.06)	1.17 (0.56, 2.42)
				RFA	34	56.1 ± 7.4	24/10	31/3			
Lencioni et al.	2003	Italy	5	RFA	52	67 ± 6	36/16	45/7	0.20 (0.02, 1.69)	0.48 (0.27, 0.85)	NA
				PEI	50	69 ± 7.4	30/20	35/15			
Lin et al.	2005	China	3	RFA	62	61 ± 10	40/22	46/16	0.42 (0.21, 0.98)	0.31 (0.18, 0.85)	2.33 (0.12, 44.15)
				PEI	62	60 ± 8	39/23	47/15			
Liu et al.	2016	China	5	TACE + RFA	100	49 (30, 76)	94/6	98/2	0.68 (0.44, 1.05)	0.69 (0.47, 1.03)	0.73 (0.37, 1.41)
				SR	100	52 (31, 80)	86/14	96/4			
Mizuki et al.	2010	Japan	4	TACE + PEI	13	65.8 ± 7.3	9/4	NA	0.62 (0.27, 1.46)	0.59 (0.20, 1.73)	NA
				PEI	14	63.6 ± 6.2	7/7	NA			
Ng et al.	2017	China	5	SR	109	55 (31, 82)	89/20	107/2	0.96 (0.71, 1.28)	0.78 (0.57, 1.08)	1.24 (0.76, 2.02)
				RFA	109	57 (23, 78)	86/23	104/5			
Orlacchio et al.	2014	Italy	4	RFA	15	71.5 ± 4.6	11/4	9/6	NA	1.79 (0.53, 6.10)	1.83 (0.46, 7.22)
				LA	15	73.5 ± 6.7	10/5	10/5			
Park et al.	2021	Korea	2.5	RFA	58	68.4 ± 8.9	48/10	58/0	NA	1.23 (0.85, 1.79)	0.94 (0.33, 2.61)
				NT-RFA	58	68.2 ± 7.9	45/13	58/0			
Shibata et al.	2002	Japan	4	RFA	36	63.6 (44, 83)	26/10	21/15	NA	NA	0.55 (0.06, 4.66)
				MWA	36	62.5 (52, 74)	24/12	19/17			
Shibata et al.	2009	Japan	3	TACE + RFA	46	67.2 ± 8.9	31/15	32/14	0.89 (0.36, 2.18)	0.92 (0.27, 3.06)	0.93 (0.06, 14.48)
				RFA	43	69.8 ± 8.0	33/10	33/10			
Song et al.	2024	China	5	SR	75	53.3 ± 10.4	63/12	74/1	0.79 (0.43, 1.45)	0.75 (0.48, 1.16)	1.55 (0.74, 3.26)
				RFA	75	53.7 ± 12.0	65/10	72/3			
Sugimoto et al.	2025	Japan	4	RFA	117	74.1 ± 9.0	77/40	111/6	1.00 (0.97, 1.03)	1.18 (0.79, 1.77)	1.00 (0.76, 1.32)
				MWA	119	74 ± 9.9	81/38	112/7			
Suh et al.	2021	Korea	2.5	RFA	38	62.5 ± 7.7	27/11	38/0	NA	1.28 (0.39, 4.24)	1.35 (0.13, 14.28)
				NT-RFA	38	66.1 ± 11.8	27/11	38/0			
Takayama et al.	2022	Japan	3	SR	150	68 (63, 74)	112/38	139/11	NA	0.92 (0.67, 1.25)	2.84 (0.16, 50.94)
				RFA	151	69 (63, 74)	108/43	149/2			
Vietti et al.	2018	France and Switzerland	4	MWA	71	68 (60, 72)	59/12	57/14	0.93 (0.47, 1.84)	0.72 (0.44, 1.18)	NA
				RFA	73	65 (59, 73)	62/11	53/20			
Vogl et al.	2024	Germany	5	RFA	25	62.7 ± 10.8	19/6	NA	1.14 (0.42, 3.08)	2.21 (1.07, 4.56)	NA
				MWA	25	63.2 ± 10.3	19/6	NA			
Wang et al.	2015	China	4	CA	180	53.9 ± 9.6	140/40	121/59	0.95 (0.82, 1.10)	0.95 (0.65, 1.40)	1.07 (0.37, 3.12)
				RFA	180	53.3 ± 8.9	150/30	109/71			
Wei et al.	2023	China	5	RT + SR	30	49 (30, 70)	22/8	28/2	0.62 (0.22, 1.76)	0.71 (0.32, 1.57)	2.60 (0.15, 46.18)
				SR	30	52 (36, 70)	23/7	28/2			
Xi et al.	2024	China	5	RT	83	56.0 (50.0, 64.0)	74/9	83/0	0.91 (0.37, 2.22)	0.76 (0.50, 1.15)	0.94 (0.74, 1.19)
				RFA	83	58.0 (51.0, 64.0)	78/5	83/0			
Yu et al.	2017	China	5	MWA	203	NA	NA	NA	0.98 (0.50, 1.91)	0.76 (0.54, 1.06)	1.15 (0.37, 3.56)
				RFA	200	NA	NA	NA			
Zhang et al.	2021	China	7	TACE + RFA	94	53.3 ± 11.1	75/19	90/4	0.55 (0.39, 0.78)	0.66 (0.49, 0.89)	NA
				RFA	95	55.3 ± 13.3	71/24	90/5			
Zhang et al.	2022	China	4	IRE	78	58.88 ± 7.95	61/17	NA	NA	1.00 (0.97, 1.03)	0.86 (0.31, 2.35)
				RFA	74	57.29 ± 8.40	59/15	NA			
Zhang et al.	2025	China	5	TACE + RFA	105	53 ± 12	100/5	NA	1.34 (0.81, 2.23)	1.05 (0.76, 1.45)	0.79 (0.52, 1.21)
				SR	105	51 ± 11	94/11	NA			

CA: Cryoablation; CI: Confidence interval; DEM-TACE: Drug-eluting microsphere transcatheter arterial chemoembolization; HR: Hazard ratio; IRE: Irreversible electroporation; LA: Laser ablation; MWA: Microwave ablation; NA: Not applicable; NT-RFA: No-touch radiofrequency ablation; OS: Overall survival; PEI: Percutaneous ethanol injection; RFA: Radiofrequency ablation; RFS: Recurrence-free survival; RR: Risk ratio; RT: Radiotherapy; SD: Standard deviation; SR: Surgical resection; TACE: Transcatheter arterial chemoembolization.

a When OS or RFS were not explicitly reported, we estimated the HR and corresponding 95%CI according to the Kaplan–Meier curve from the publications.

b For groups containing 0 event, we did continuity correction.

c The number of Child-Pugh A/B patients in this study was calculated according to the mean and standard deviation of Child-Pugh scores provided by this study, based on the assumption that the Child-Pugh score in this study approximately follows normal distribution.

d This study comprises 2 subgroups. For one subgroup, the tumor size was ≤3 cm, and for another, the tumor size was >3 cm, but ≤5 cm. In the analysis of OS and RFS, this study was divided into two studies according to the tumor size; for the analysis of complications, the two subgroups were combined.

e In this study, subgroup-specific data on sex distribution and complications were not available for the two tumor-size subgroups. Accordingly, the overall sex distribution reported in the original trial is presented for each subgroup, whereas the complication RR was calculated using the combined trial-level data and was not double-counted in the complications analysis.

### Primary outcomes

#### Overall survival

**Fig. 2:**
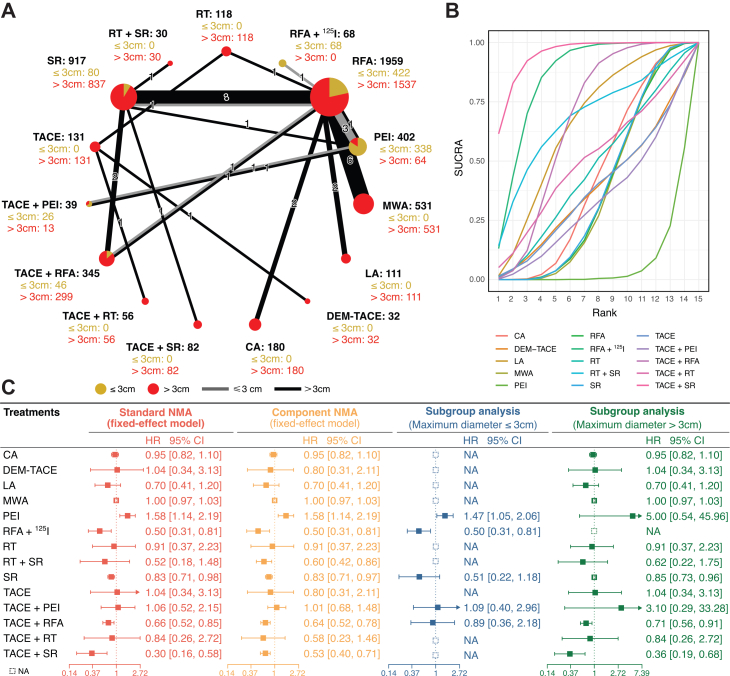
**The comparison of OS among the treatments.** (A)Network plot; (B)SUCRA plot of the network meta-analysis; (C)Forest plots. CA, cryoablation; CI, confidence interval; DEM-TACE, drug-eluting microsphere transcatheter arterial chemoembolization; HR, hazard ratio; LA, laser ablation; MWA, microwave ablation; NMA, network meta-analysis; PEI, percutaneous ethanol injection; RFA, radiofrequency ablation; RT, radiotherapy; SR, surgical resection; SUCRA, surface under the cumulative ranking curve; TACE, transcatheter arterial chemoembolization.

Thirty-four RCTs involving 5001 patients were identified for overall survival (Fig. 2A). Notably, the RCT published by Chen et al. in 2006 was treated as two studies according to the size of tumor. Judging from the SUCRA (Fig. 2B), TACE + SR, RFA + ^125^I, TACE + RFA, MWA, and SR were among the best treatments. Compared with RFA, TACE + SR (HR 0.30, 95% CI 0.16–0.58, high certainty), RFA + ^125^I (HR 0.50, 95% CI 0.31–0.81, high certainty), TACE + RFA (HR 0.66, 95% CI 0.52–0.85, moderate certainty), and SR (HR 0.83, 95% CI 0.71–0.98, high certainty) were significantly better in standard NMA model (Fig. 2C, Supplementary Fig. S2). In the component NMA model, TACE + SR (HR 0.53, 95% CI 0.40–0.71), RFA + ^125^I (HR 0.50, 95% CI 0.31–0.81), TACE + RFA (HR 0.64, 95% CI 0.52–0.78), and SR (HR 0.83, 95% CI 0.71–0.97) remained significantly better than RFA. Incremental effects (Supplementary Table S2) indicated that ^125^I implantation (iHR 0.50, 95% CI 0.31–0.81), DEM-TACE (iHR 0.64, 95% CI 0.52–0.79), and TACE (iHR 0.64, 95% CI 0.52–0.78) may significantly enhance the therapeutic effects of other treatments. No significant heterogeneity or inconsistency was observed in either model (Supplementary Fig. S4 and Table S3), and no evidence of publication bias was detected (Supplementary Fig. S5A). In the subgroup analysis, most effect sizes remained constant between the groups (Fig. 2C). No significant difference was observed between the results of network meta-regression and standard NMA (Fig. 2C), and the ratio of patients with Child–Pugh class A liver function was not a significant covariate (Supplementary Table S4).

#### Recurrence-free survival

**Fig. 3:**
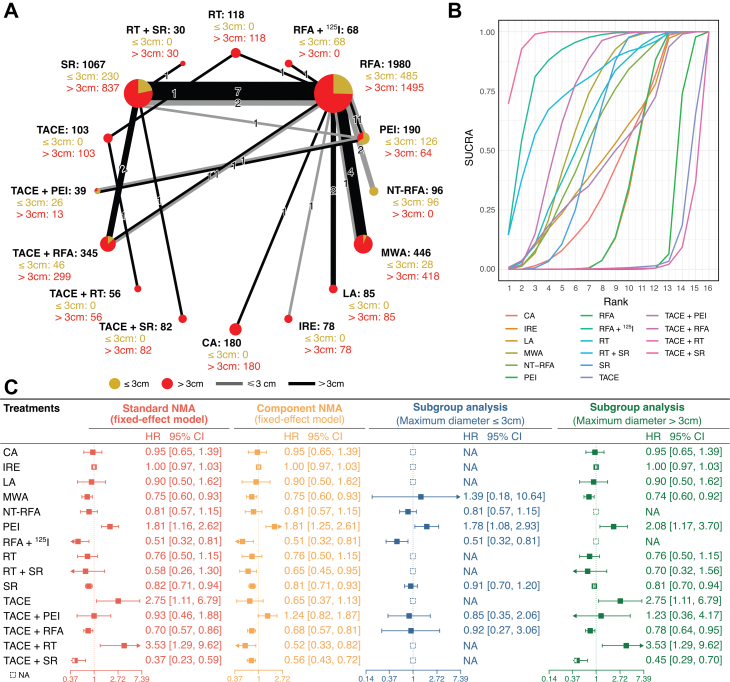
**The comparison of RFS among the treatments.** (A)Network plot; (B)SUCRA plot of the network meta-analysis; (C)Forest plots. CA, cryoablation; CI, confidence interval; HR, hazard ratio; IRE, irreversible electroporation; LA, laser ablation; MWA, microwave ablation; NMA, network meta-analysis; NT-RFA, no-touch radiofrequency ablation; PEI, percutaneous ethanol injection; RFA, radiofrequency ablation; RT, radiotherapy; SR, surgical resection; SUCRA, surface under the cumulative ranking curve; TACE, transcatheter arterial chemoembolization.

Thirty-four RCTs involving 4963 patients were identified for recurrence-free survival (Fig. 3A). The two subgroups of the RCT published by Chen et al. in 2006 were also treated as two studies. According to the SUCRA (Fig. 3B), TACE + SR, MWA, TACE + RFA, and SR were among the best treatments. Compared with RFA, TACE + SR (HR 0.37, 95% CI 0.23–0.59, high certainty), RFA + ^125^I (HR 0.51, 95% CI 0.32–0.81, high certainty), TACE + RFA (HR = 0.70, 95% CI 0.57–0.86, high certainty), MWA (HR 0.75, 95% CI 0.60–0.93, high certainty), and SR (HR 0.82, 95% CI 0.71–0.94, high certainty) had significantly better effects in elongating RFS, while TACE alone has worse RFS (HR 2.75, 95% CI 1.11–6.79, moderate certainty), according to the standard NMA model (Fig. 3C, Supplementary Fig. S2). In the component NMA model, TACE + SR (HR 0.56, 95% CI 0.43–0.72), RFA + ^125^I (HR 0.51, 95% CI 0.32–0.81), TACE + RFA (HR 0.68, 95% CI 0.57–0.81), MWA (HR 0.75, 95% CI 0.60–0.93), and SR (HR 0.81, 95% CI 0.71–0.93) remained significantly better than RFA. The incremental effects (Supplementary Table S2) indicated that the addition of ^125^I implantation (iHR 0.51, 95% CI 0.32–0.81) and TACE (iHR 0.68, 95% CI 0.57–0.81) may significantly improve the prognosis of other treatments, while PEI (iHR 1.91, 95% CI 0.99–3.68) may lead to a worse prognosis of other treatments, though narrowly missing statistical significance. No significant heterogeneity or inconsistency was observed in either model (Supplementary Fig. S4 and Table S3), nor was evidence of publication bias detected (Supplementary Fig. S5B). The subgroup analysis revealed that MWA (HR 1.39, 95% CI 0.18–10.64) and TACE + RFA (HR 0.92, 95% CI 0.27–3.06) were not significantly better than RFA in patients with tumors no greater than 3 cm (Fig. 3C). No significant difference was observed between the results of network meta-regression and standard NMA (Fig. 3C). Interestingly, the proportion of patients with Child–Pugh class A liver function was negatively associated with the hazard ratio for recurrence-free survival in network meta-regression model (*r* −0.16, 95% CI −0.28 to −0.04). This may indicate that studies enrolling a higher proportion of Child–Pugh A patients tended to show better RFS outcomes (Supplementary Table S4).

### Secondary outcome

**Fig. 4:**
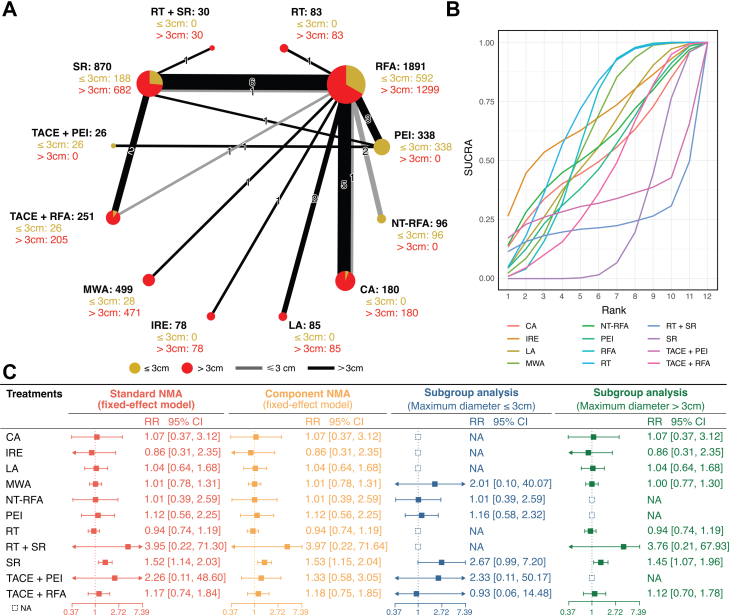
**The comparison of complications among the treatments.** (A)Network plot; (B)SUCRA plot of the network meta-analysis; (C)Forest plots. CA, cryoablation; CI, confidence interval; RR, relative risk; IRE, irreversible electroporation; LA, laser ablation; MWA, microwave ablation; NMA, network meta-analysis; NT-RFA, no-touch radiofrequency ablation; PEI, percutaneous ethanol injection; RFA, radiofrequency ablation; RT, radiotherapy; SR, surgical resection; SUCRA, surface under the cumulative ranking curve; TACE, transcatheter arterial chemoembolization.

Twenty-nine RCTs involving 4427 patients were identified for the incidence of complications (Fig. 4A). The two subgroups of the RCT published by Chen et al. in 2006 were also treated as two studies. Judging from the SUCRA (Fig. 4B), IRE, RT, RFA, MWA were among the safest treatments, while SR (RR, 1.52, 95%CI, 1.14–2.03, high certainty) may have higher incidence of complications. Compared with RFA, only SR showed a significantly higher incidence of complications in the standard NMA model, and this finding remained significant in the component NMA, subgroup analyses, and meta-regression models (Fig. 4C). No other significant differences in the incidence of complications were observed. A moderate inconsistency was detected in the loop connecting RT, SR, and RFA, whereas no substantial heterogeneity or other inconsistency was found in either model (Supplementary Fig. S4 and Table S3). In addition, no evidence of publication bias was identified (Supplementary Fig. S5C).

### Weighted correlation analysis at study level

We conducted two weighted correlation analyses at study level between hazard ratios (HRs) for overall survival (OS) or recurrence-free survival (RFS) and the risk ratio (RR) of complications respectively, to examine whether greater therapeutic benefit was associated with a higher incidence of complications. No significant correlation was found in either regression (HR_OS_ ∼ RR_Complications_: slope −0.02, 95% CI −1.54 to 1.49; HR_RFS_ ∼ RR_Complications_: slope −0.18, 95% CI −2.81 to 2.45; Supplementary Fig. S6).

## Discussion

This study is an updated systematic review focusing on the efficacy and safety of several therapies and their combinations for SHCC, incorporating 16 additional RCTs. Notably, only RCTs were included, and the synergistic or antagonistic effects of different regimens were evaluated using component network meta-analysis (CNMA). Because this study specifically targeted SHCC, for which the primary therapeutic aim is complete tumor ablation, generalized recurrence-free survival is of particular importance.^1^ Additionally, the meta-regression analysis between OS/RFS and RR of complications revealed no statistically significant correlation, indicating that efficacy doesn’t increase at the expense of more complications.

SR remains the first-line treatment for patients with SHCC, and our study confirms its ability to achieve complete tumor eradication. Anatomical resection (e.g., segmental or lobar resection) aims to remove the primary lesion together with potential microscopic satellite foci, and most procedures are performed with curative intent for SHCC.^1^^,^^25^ In our analysis, SR remained an important curative-intent option and showed favorable survival outcomes. However, SR was associated with a significantly higher incidence of complications than RFA, highlighting the need for careful patient selection and perioperative liver-function assessment.^26^ Among all currently available treatments for SHCC, SR had the highest complication rate, with post-hepatectomy liver failure (pHLF) representing a particularly critical and potentially lethal postoperative event.^27^ Notably, our meta-regression results showed that a higher proportion of Child–Pugh A patients was associated with a greater RFS benefit (*r* −0.16, 95% CI −0.28 to −0.04), suggesting the importance of preoperative liver function in patients undergoing SR for HCC. Given HCC frequently arises in livers with comorbid hepatitis and/or cirrhosis,^28^^,^^29^ better preoperative liver function implies milder inflammation and a larger functional remnant, which may contribute to fewer complications and delayed recurrence,^30^^,^^31^ consistent with evidence that preoperative liver function is an independent prognostic factor in patients undergoing SR for HCC.^32^

Remnant liver function should therefore be central to both the evaluation of surgical eligibility and the choice of procedure. European association for the study of the liver (EASL) guidelines recommend volumetric assessment, the indocyanine green retention test, and at least one laboratory-based liver function score for perioperative risk stratification.^1^^,^^33^ In patients with centrally located tumors, multiple lesions, and/or compromised liver function, SR may be inappropriate because an adequate functional remnant cannot be preserved.

In this context, locoregional therapies may serve as alternative treatment options.^34^ We compared a range of locoregional modalities, including ablation techniques (RFA, NT-RFA, MWA, CA, and LA), radiotherapy, PEI, IRE, TACE, and DEM-TACE, which differ in their mechanisms of tumor destruction but share the goal of local tumor control.^35^ Many locoregional therapies were applied in patients with tumors ≥3 cm; however, we did not observe a significant difference in efficacy between subgroups with tumors >3 cm or ≤3 cm, supporting that tumor size alone is not the key factor determining the efficacy of local therapy.

Traditional RFA delivers high-frequency alternating current through an electrode placed within the tumor, thereby generating frictional heat that induces coagulative necrosis of cancer cells within a defined ablation zone. RFA is currently recommended as a first-line alternative for patients who are not eligible for SR,^24^^,^^36^^,^^37^ and our analysis also revealed that RFA achieved comparable OS but significantly fewer complications than SR in SHCC. However, its efficacy may be compromised by the heat-sink effect, which can lead to incomplete ablation of tumors adjacent to large vessels. In addition, conventional intratumoral RFA carries a reported 0.2–0.5% risk of needle-tract seeding,^38^ whereby tumor cells are disseminated along the puncture pathway. NT-RFA is designed to reduce the risk of needle-tract seeding, as the electrodes are placed in the peritumoral liver parenchyma rather than puncturing the tumor itself, thereby creating an ablative margin that encompasses the entire lesion.^39^ In this study, NT-RFA showed a trend toward improved RFS (HR 0.81, 95% CI 0.57 to 1.15).

In contrast to RFA, microwave ablation (MWA) uses electromagnetic waves to induce dielectric heating of tissue water molecules, thereby achieving more rapid and homogeneous intratumoral heating.^40^^,^^41^ This mechanism allows MWA to create larger and more predictable ablation zones and to be less susceptible to the heat-sink effect near large vessels, resulting in more complete tumor necrosis in prespecified lesions.^40^ Our results supported that MWA was associated with superior RFS compared with RFA (HR 0.75, 95%CI 0.60–0.93). LA and CA induce tumor necrosis by laser energy and low temperature, respectively, while no statistically significant differences were observed between these treatments and RFA.^42^^,^^43^

The ablation range of thermal techniques is not precisely confined and may injure surrounding critical structures (e.g., bile ducts, major vessels and the gallbladder). Irreversible electroporation (IRE), a non-thermal ablation modality introduced in the mid-2000s, induces apoptotic cell death while largely preserving adjacent bile ducts and vessels.^44^ In our study, IRE achieved OS, RFS and morbidity rates comparable to those of RFA, and may be particularly^45^ attractive for tumors near major vascular or biliary structures, where thermal ablation is constrained.

Alternatively, radiotherapy may serve as a complement when SR and ablation are not indicated.^46^ Although current evidence for RT remains limited, the four included RCTs suggested efficacy and safety comparable to RFA or SR. Stereotactic body radiotherapy (SBRT) is generally regarded as the preferred RT modality for HCC, although the total dose must be limited to avoid radiation-induced liver disease.^46^, ^47^, ^48^

Among radiation-related strategies, RFA + ^125^I showed consistent improvement in both OS and RFS compared with RFA.^49^ This may be because iodine-125 seed implantation provides continuous low-dose irradiation around the ablation zone, potentially eliminating residual microscopic disease that survives thermal ablation. Unlike external-beam RT, this approach delivers highly localized radiation and may therefore complement RFA without substantially increasing injury to non-tumorous liver parenchyma. However, the number of trials remains limited, and further validation is needed before this strategy can be generalized.

Some studies have also investigated PEI, but this technique is no longer recommended as a standard option in contemporary clinical practice.^1^ In fact, our results indicated that PEI showed the worst OS and RFS compared with other local treatments, so we may not recommend it as the first-line option for local treatment in SHCC. Accordingly, most current guidelines state that PEI should only be considered when thermal ablation is unavailable or contraindicated, such as in settings with limited healthcare resources or inadequate technical conditions.^50^

Additionally, an increasing number of randomized controlled trials have focused on combination strategies for SHCC, in which TACE appears to have context-dependent effects. TACE delivers chemotherapeutic agents directly into the tumor-feeding hepatic arteries, followed by arterial embolization that induces ischemic and chemotherapeutic tumor necrosis.^51^, ^52^, ^53^ DEM-TACE was developed to improve sustained local drug delivery and reduce systemic exposure, but our analysis did not show a statistically significant efficacy difference between conventional TACE and DEM-TACE. Notably, TACE alone was associated with worse RFS than RFA, whereas TACE-containing combinations, particularly TACE + RFA and TACE + SR, showed favorable survival outcomes. This contrast suggests that TACE may be insufficient as a curative-intent monotherapy for SHCC, but may complement ablation or resection by reducing tumor arterial perfusion, decreasing tumor burden, and treating microscopic residual disease or satellite lesions that are not fully eliminated by local therapy alone.^54^^,^^55^ Therefore, the favorable results of TACE + RFA and TACE + SR, which exceeded the effects expected from the additive contribution of individual treatment components, should be interpreted as CNMA-derived signals of potential synergistic effects, rather than definitive proof of biological synergy.

Interestingly, the combination between RT and TACE seems to be antagonistic in OS and RFS. From a biological perspective, the primary mechanism of TACE is to induce profound ischemia and hypoxia by embolizing the tumor-feeding arteries. However, the radiosensitivity of tumor tissue may also be reduced because the low oxygen environment may relieve radiation-induced DNA damage and make tumor cells more radioresistant.^56^ In addition, TACE has been shown to upregulate hypoxia-inducible and pro-angiogenic pathways such as HIF-1α and VEGF in the tumor microenvironment, which may promote neovascularization, facilitate distant recurrence and further contribute to radioresistance.^57^ Furthermore, both TACE and RT directly target the liver, and their combination can exert cumulative hepatotoxicity. TACE-induced ischemic injury and altered portal flow, together with the cumulative radiation dose to non-tumorous liver parenchyma, increase the risk of radiation-induced liver disease and hepatic decompensation.^58^ Once liver function deteriorates, subsequent local or systemic treatments become limited, which may blunt any survival benefit from improved local control. These mechanistic considerations are consistent with the discrepancy between our NMA and CNMA results, in which TACE-RT combinations appeared antagonistic. Other intra-arterial modalities such as hepatic arterial infusion chemotherapy (HAIC) have shown promising results in patients with advanced HCC or portal vein tumor thrombosis, but their role in SHCC remains poorly defined and was beyond the scope of the present analysis.^59^

This study had several limitations. The impact of tumor size and number on clinical decision-making remains incompletely understood, despite our use of meta-regression analyses. Second, bias exists in the design of some RCTs included in this study, indicated by the RoB scores. Thus, additional RCTs with rigorous design and conduct specifically targeting SHCC are warranted to enable direct comparisons between treatment modalities. Finally, future trials that stratify patients by clinicopathological and molecular characteristics are needed to inform precision treatment strategies in this field.

In conclusion, this study shows that SR remains the foundational curative option for SHCC, but TACE + SR exhibits superior efficacy. Meanwhile, comprehensive preoperative assessment of liver function and precise perioperative management are critical to reduce possible complications. When SR is not indicated, most locoregional treatments could provide comparable therapeutic efficacy and safety, among which RFA + ^125^I shows slightly better efficacy over other ablation methods. Combination strategies generally provide greater benefit than monotherapies, and our findings support the use of RFA + ^125^I seed implantation, TACE + SR, and TACE plus RFA in appropriately selected patients, whereas the PEI and combination of TACE and RT should be adopted with caution.

## Contributors

Peiyan Sun: study design, data analysis, data access and verification, and writing the manuscript; Jianlin Wu: data collection and analysis, data access and verification, and writing the manuscript; Dong Cai: data collection and analysis, data access and verification, and writing the manuscript; Tianrun Lv: data collection and analysis, and writing the manuscript; Hailin Tang: data analysis, polish the manuscript; Yanwen Jin: data analysis, polish the manuscript; Fuyu Li: study design, data access and verification, and polish the manuscript; Haijie Hu: study design, data access and verification, and polish the manuscript.

## Data sharing statement

The meta-analysis was performed based on previously-published studies and the original data was extracted from these studies and can be provided if required. All data generated or analyzed during this study is included in this article.

## Declaration of interests

There is no conflict of interest.
