# Supplementary material for: Efficacy and safety of multiple treatments for small hepatocellular carcinoma: an updated systematic review and component network meta analysis
Source: eClinicalMedicine. 2026 Jun 23;97:104037. doi: 10.1016/j.eclinm.2026.104037 (PMC13316238; doi:10.1016/j.eclinm.2026.104037)
Supplement: Supplementary material [file mmc1.pdf]

Efficacy and Safety of Multiple Treatments for Small Hepatocellular Carcinoma: An Updated Systematic Review and Component Network Meta Analysis

Supplementary Materials

Peiyan Sun[1,#], Jianlin Wu[1,#], Dong Cai[1,#], Tianrun Lv[1], Yanwen Jin[1], Hailin Tang[2], Fuyu Li[1,*], Haijie Hu[1,*]

March 2026

[1] Division of Biliary Surgery, Department of General Surgery, West China Hospital, Sichuan University, Chengdu, Sichuan Province, China

[2] State Key Laboratory of Oncology in South China, Sun Yat-Sen University Cancer Center, Guangzhou, China

[#] These authors contributed equally to this study.

[*] Corresponding authors

Contents

1 Supplementary Figures **3**

1.1 Supplementary Figure 1: League table and GRADE certainty of the outcomes . . . 3

1.2 Supplementary Figure 2: Forest plots for standard network meta analysis (fixed-effect model) and network meta regression (fixed effect model) . . . 6

1.3 Supplementary Figure 3: RoB 2.0 assessment of the randomized controlled trials included in this study . . . 7

1.4 Supplementary Figure 4: The inconsistency of the standard network meta analysis . . . 8

1.5 Supplementary Figure 5: Funnel plots of the outcomes . . . 9

1.6 Supplementary Figure 6: Correlation analysis between overall survival or recurrence-free survival (lnHR) and complications (lnRR) at study levels . . . 10

2 Supplementary Tables **11**

2.1 Supplementary Table 1: Treatments included in this study for small hepatocellular carcinoma . . . 11

2.2 Supplementary Table 2: Incremental effects of the treatment components . . . 12

2.3 Supplementary Table 3: Overall heterogeneity of the outcomes . . . 13

2.4 Supplementary Table 4: Network Meta-regression for the outcomes . . . 14

2.5 Supplementary Table 5: GRADE assessment of the comparisons according to the NMA result . . . 15

3 Appendix 1: Search Strategy **22**

3.1 PubMed . . . 22

3.2 Embase . . . 22

3.3 Cochrane . . . 22

4 Appendix 2: Supplementary Methods **23**

5 **Appendix 3: RoB 2.0 assessment of the RCTs** **24**
5.1 Abdelaziz2014 . . . 24
5.2 Brunello2008 . . . 27
5.3 Bush2023 . . . 30
5.4 Chen2006 . . . 33
5.5 Chen2014 . . . 36
5.6 Chong2020 . . . 39
5.7 Costanzo2015 . . . 41
5.8 Fang2014 . . . 43
5.9 Fang2023 . . . 45
5.10 Feng2012 . . . 47
5.11 Féray2023 . . . 49
5.12 Ferrari2007 . . . 51
5.13 Giorgio2011 . . . 54
5.14 Gjoreski2021 . . . 56
5.15 Huang2005 . . . 58
5.16 Huang2010 . . . 61
5.17 Kamal2019 . . . 64
5.18 Koda2001 . . . 66
5.19 Lee2018 . . . 69
5.20 Lencioni2003 . . . 71
5.21 Lin2005 . . . 74
5.22 Liu2016 . . . 77
5.23 Mizuki2010 . . . 80
5.24 Ng2017 . . . 83
5.25 Orlacchio2014 . . . 85
5.26 Park2021 . . . 88
5.27 Shibata2002 . . . 90
5.28 Shibata2009 . . . 93
5.29 Song2024 . . . 95
5.30 Sugimoto2025 . . . 97
5.31 Suh2021 . . . 99
5.32 Takayama2022 . . . 101
5.33 Vietti2018 . . . 103
5.34 Vogl2024 . . . 105
5.35 Wang2015 . . . 107
5.36 Wei2023 . . . 109
5.37 Xi2024 . . . 111
5.38 Yu2017 . . . 113
5.39 Zhang2021 . . . 115
5.40 Zhang2022 . . . 117
5.41 Zhang2025 . . . 119

6 **The list of included RCTs** **121**

1 Supplementary Figures

A

**CA**	NA	NA	NA	NA	0.95 (0.82, 1.10)	NA	NA	NA	NA	NA	NA	NA	NA	NA
0.91 (0.30, 2.78)	**DEM-TACE**	NA	NA	NA	NA	NA	NA	NA	NA	1.00 (0.95, 1.05)	NA	NA	NA	NA
1.34 (0.78, 2.33)	1.47 (0.43, 5.01)	**LA**	NA	NA	0.70 (0.41, 1.20)	NA	NA	NA	NA	NA	NA	NA	NA	NA
0.95 (0.82, 1.10)	1.04 (0.34, 3.14)	0.71 (0.42, 1.20)	**MWA**	NA	1.00 (0.97, 1.03)	NA	NA	NA	NA	NA	NA	NA	NA	NA
**0.60 (0.42, 0.86)**	0.66 (0.21, 2.08)	**0.45 (0.24, 0.83)**	**0.63 (0.46, 0.88)**	**PEI**	**1.44 (1.03, 2.03)**	NA	NA	NA	**4.62 (1.58, 13.52)**	NA	1.49 (0.80, 2.79)	NA	NA	NA
0.95 (0.82, 1.10)	1.04 (0.34, 3.13)	0.70 (0.41, 1.20)	1.00 (0.97, 1.03)	**1.58 (1.14, 2.19)**	**RFA**	**1.99 (1.24, 3.20)**	1.10 (0.45, 2.69)	NA	1.13 (0.95, 1.34)	NA	NA	**1.71 (1.24, 2.36)**	NA	NA
**1.89 (1.15, 3.10)**	2.07 (0.62, 6.88)	1.40 (0.69, 2.86)	**1.99 (1.24, 3.19)**	**3.15 (1.77, 5.59)**	**1.99 (1.24, 3.20)**	**RFA + ^{125}I**	NA	NA	NA	NA	NA	NA	NA	NA
1.04 (0.42, 2.58)	1.14 (0.60, 2.18)	0.77 (0.27, 2.19)	1.10 (0.45, 2.69)	1.74 (0.67, 4.50)	1.10 (0.45, 2.69)	0.55 (0.20, 1.52)	**RT**	NA	NA	0.88 (0.46, 1.67)	NA	NA	NA	NA
1.83 (0.63, 5.31)	2.01 (0.44, 9.23)	1.36 (0.42, 4.43)	1.93 (0.67, 5.53)	3.06 (1.02, 9.18)	1.93 (0.67, 5.54)	0.97 (0.31, 3.08)	1.76 (0.44, 7.01)	**RT + SR**	0.62 (0.22, 1.76)	NA	NA	NA	NA	NA
1.14 (0.91, 1.41)	1.24 (0.41, 3.80)	0.84 (0.49, 1.47)	1.20 (1.02, 1.40)	**1.89 (1.33, 2.70)**	**1.20 (1.02, 1.40)**	**0.60 (0.37, 0.99)**	1.09 (0.44, 2.71)	0.62 (0.22, 1.76)	**SR**	NA	NA	1.10 (0.79, 1.53)	NA	2.78 (1.47, 5.25)
0.91 (0.30, 2.78)	1.00 (0.95, 1.05)	0.68 (0.20, 2.31)	0.96 (0.32, 2.90)	1.52 (0.48, 4.81)	0.96 (0.32, 2.91)	0.48 (0.15, 1.61)	0.88 (0.46, 1.67)	0.50 (0.11, 2.29)	0.80 (0.26, 2.45)	**TACE**	NA	NA	1.23 (0.83, 1.82)	NA
0.89 (0.43, 1.84)	0.98 (0.26, 3.63)	0.66 (0.27, 1.61)	0.94 (0.46, 1.91)	1.49 (0.80, 2.79)	0.94 (0.46, 1.91)	0.47 (0.20, 1.11)	0.86 (0.27, 2.68)	0.49 (0.14, 1.73)	0.79 (0.38, 1.62)	0.98 (0.26, 3.63)	**TACE + PEI**	NA	NA	NA
**1.43 (1.07, 1.90)**	1.56 (0.50, 4.84)	1.06 (0.59, 1.90)	**1.50 (1.18, 1.92)**	**2.38 (1.59, 3.56)**	**1.51 (1.18, 1.92)**	0.76 (0.44, 1.29)	1.37 (0.54, 3.47)	0.78 (0.27, 2.27)	**1.26 (0.98, 1.60)**	1.56 (0.50, 4.84)	1.60 (0.76, 3.37)	**TACE + RFA**	NA	NA
1.12 (0.34, 3.66)	1.23 (0.83, 1.83)	0.84 (0.23, 3.02)	1.18 (0.37, 3.82)	1.87 (0.56, 6.32)	1.19 (0.37, 3.82)	0.59 (0.17, 2.10)	1.08 (0.51, 2.29)	0.61 (0.13, 2.96)	0.99 (0.30, 3.23)	1.23 (0.83, 1.82)	1.26 (0.32, 4.94)	0.79 (0.24, 2.60)	**TACE + RT**	NA
**3.15 (1.61, 6.18)**	3.45 (0.96, 12.49)	**2.35 (1.01, 5.46)**	**3.32 (1.72, 6.41)**	**5.26 (2.54, 10.91)**	**3.33 (1.73, 6.42)**	1.67 (0.74, 3.75)	3.03 (1.00, 9.20)	1.72 (0.51, 5.84)	**2.78 (1.47, 5.25)**	3.45 (0.96, 12.48)	**3.53 (1.35, 9.25)**	**2.21 (1.12, 4.38)**	2.81 (0.73, 10.76)	**TACE + SR**

Supplementary Figure 1: League table and GRADE certainty of the outcomes

The left lower cells represent the NMA result between two treatments labelled on the terminals of the columns and the rows, and the right upper cells represent the direct comparison between the corresponding treatments. A: Overall survival (HR and 95% confidence intervals); B: Recurrence-free survival (HR and 95% confidence intervals); C: Complications (RR and 95% confidence intervals); ^{125}I: Iodine-125 seed implantation; CA: Cryoablation; DEM-TACE: Drug-eluting microsphere transcatheter arterial chemoembolization; IRE: Irreversible electroporation; LA: Laser ablation; MWA: Microwave ablation; NT-RFA: No-touch radiofrequency ablation; PEI: Percutaneous ethanol injection; RFA: Radiofrequency ablation; RT: Radiotherapy; SR: Surgical resection; TACE: Transcatheter arterial chemoembolization.

Legend:

- High certainty
- Moderate certainty
- Low certainty
- Very low certainty

B

**CA**	NA	NA	NA	NA	NA	0.95 (0.65, 1.39)	NA	NA	NA	NA	NA	NA	NA	NA	NA
0.95 (0.65, 1.40)	**IRE**	NA	NA	NA	NA	1.00 (0.97, 1.03)	NA	NA	NA	NA	NA	NA	NA	NA	NA
1.06 (0.52, 2.13)	1.11 (0.62, 2.00)	**LA**	NA	NA	NA	0.90 (0.50, 1.62)	NA	NA	NA	NA	NA	NA	NA	NA	NA
1.27 (0.82, 1.98)	**1.34 (1.08, 1.67)**	1.21 (0.64, 2.25)	**MWA**	NA	NA	0.75 (0.60, 0.93)	NA	NA	NA	NA	NA	NA	NA	NA	NA
1.18 (0.70, 1.99)	1.24 (0.87, 1.77)	1.11 (0.56, 2.21)	0.92 (0.61, 1.40)	**NT-RFA**	NA	0.81 (0.57, 1.15)	NA	NA	NA	NA	NA	NA	NA	NA	NA
**0.52 (0.31, 0.89)**	**0.55 (0.38, 0.80)**	**0.50 (0.25, 0.99)**	**0.41 (0.27, 0.63)**	**0.44 (0.27, 0.74)**	**PEI**	2.43 (1.53, 3.86)	NA	NA	NA	1.38 (0.77, 2.48)	NA	1.95 (1.07, 3.57)	NA	NA	NA
0.95 (0.65, 1.39)	1.00 (0.97, 1.03)	0.90 (0.50, 1.62)	**0.75 (0.60, 0.93)**	0.81 (0.57, 1.15)	**1.81 (1.26, 2.62)**	**RFA**	1.97 (1.23, 3.16)	1.32 (0.87, 2.00)	NA	1.25 (1.07, 1.46)	NA	NA	1.49 (1.11, 1.99)	NA	NA
**1.87 (1.02, 3.44)**	**1.97 (1.23, 3.16)**	1.77 (0.83, 3.76)	1.47 (0.87, 2.47)	1.59 (0.88, 2.87)	**3.57 (1.97, 6.49)**	**1.97 (1.23, 3.16)**	**RFA + ^{125}I**	NA	NA	NA	NA	NA	NA	NA	NA
1.25 (0.71, 2.20)	1.32 (0.87, 2.00)	1.18 (0.58, 2.43)	0.98 (0.61, 1.57)	1.06 (0.61, 1.84)	**2.39 (1.37, 4.16)**	1.32 (0.87, 2.00)	0.67 (0.36, 1.25)	**RT**	NA	NA	0.28 (0.12, 0.62)	NA	NA	NA	NA
1.64 (0.67, 4.00)	1.72 (0.77, 3.86)	1.55 (0.57, 4.20)	1.28 (0.56, 2.96)	1.39 (0.57, 3.36)	**3.12 (1.30, 7.52)**	1.72 (0.77, 3.86)	0.87 (0.34, 2.23)	1.31 (0.53, 3.25)	**RT + SR**	0.71 (0.32, 1.57)	NA	NA	NA	NA	NA
1.16 (0.77, 1.75)	**1.22 (1.06, 1.41)**	1.10 (0.60, 2.01)	0.91 (0.70, 1.18)	0.99 (0.67, 1.45)	**2.22 (1.53, 3.22)**	**1.22 (1.06, 1.41)**	0.62 (0.38, 1.02)	0.93 (0.60, 1.44)	0.71 (0.32, 1.57)	**SR**	NA	NA	1.13 (0.88, 1.45)	NA	2.22 (1.42, 3.48)
**0.35 (0.13, 0.92)**	**0.36 (0.15, 0.90)**	**0.33 (0.11, 0.96)**	**0.27 (0.11, 0.69)**	**0.29 (0.11, 0.77)**	0.66 (0.25, 1.75)	**0.36 (0.15, 0.90)**	**0.18 (0.07, 0.51)**	**0.28 (0.12, 0.62)**	**0.21 (0.06, 0.71)**	**0.30 (0.12, 0.74)**	**TACE**	NA	NA	0.78 (0.50, 1.21)	NA
1.02 (0.46, 2.28)	1.08 (0.53, 2.18)	0.97 (0.39, 2.42)	0.80 (0.38, 1.68)	0.87 (0.39, 1.92)	**1.95 (1.07, 3.57)**	1.08 (0.53, 2.18)	0.55 (0.23, 1.28)	0.82 (0.36, 1.86)	0.63 (0.22, 1.81)	0.88 (0.43, 1.79)	2.96 (0.94, 9.32)	**TACE + PEI**	NA	NA	NA
1.35 (0.88, 2.09)	**1.42 (1.16, 1.75)**	1.28 (0.69, 2.39)	1.06 (0.79, 1.43)	1.15 (0.76, 1.73)	**2.58 (1.72, 3.89)**	**1.42 (1.16, 1.75)**	0.72 (0.43, 1.21)	1.08 (0.68, 1.72)	0.83 (0.36, 1.88)	1.17 (0.96, 1.42)	**3.92 (1.55, 9.89)**	1.32 (0.64, 2.75)	**TACE + RFA**	NA	NA
**0.27 (0.09, 0.79)**	**0.28 (0.10, 0.77)**	**0.25 (0.08, 0.82)**	**0.21 (0.08, 0.59)**	**0.23 (0.08, 0.66)**	0.51 (0.18, 1.50)	**0.28 (0.10, 0.77)**	**0.14 (0.05, 0.44)**	**0.22 (0.09, 0.54)**	**0.16 (0.05, 0.60)**	**0.23 (0.08, 0.64)**	0.78 (0.50, 1.21)	**0.26 (0.08, 0.90)**	**0.20 (0.07, 0.55)**	**TACE + RT**	NA
**2.58 (1.41, 4.73)**	**2.72 (1.70, 4.35)**	**2.44 (1.15, 5.18)**	**2.03 (1.21, 3.40)**	**2.19 (1.22, 3.95)**	**4.93 (2.75, 8.82)**	**2.72 (1.70, 4.34)**	1.38 (0.71, 2.68)	2.06 (1.10, 3.87)	1.58 (0.63, 3.93)	**2.22 (1.42, 3.48)**	**7.47 (2.70, 20.68)**	2.52 (1.09, 5.84)	**1.91 (1.17, 3.11)**	**9.58 (3.16, 29.01)**	**TACE + SR**

Supplementary Figure 1: League table and GRADE certainty of the outcomes *(continued)*

The left lower cells represent the NMA result between two treatments labelled on the terminals of the columns and the rows, and the right upper cells represent the direct comparison between the corresponding treatments. A: Overall survival (HR and 95% confidence intervals); B: Recurrence-free survival (HR and 95% confidence intervals); C: Complications (RR and 95% confidence intervals); ^{125}I: Iodine-125 seed implantation; CA: Cryoablation; DEM-TACE: Drug-eluting microsphere transcatheter arterial chemoembolization; IRE: Irreversible electroporation; LA: Laser ablation; MWA: Microwave ablation; NT-RFA: No-touch radiofrequency ablation; PEI: Percutaneous ethanol injection; RFA: Radiofrequency ablation; RT: Radiotherapy; SR: Surgical resection; TACE: Transcatheter arterial chemoembolization.

Legend:
- High certainty
- Moderate certainty
- Low certainty
- Very low certainty

C

**CA**	NA	NA	NA	NA	NA	1.07 (0.37, 3.12)	NA	NA	NA	NA	NA
1.25 (0.29, 5.43)	**IRE**	NA	NA	NA	NA	0.86 (0.31, 2.35)	NA	NA	NA	NA	NA
1.03 (0.32, 3.34)	0.83 (0.27, 2.53)	**LA**	NA	NA	NA	1.04 (0.64, 1.68)	NA	NA	NA	NA	NA
1.06 (0.35, 3.20)	0.85 (0.30, 2.41)	1.03 (0.60, 1.78)	**MWA**	NA	NA	1.01 (0.78, 1.31)	NA	NA	NA	NA	NA
1.06 (0.25, 4.42)	0.85 (0.21, 3.38)	1.03 (0.36, 2.96)	1.00 (0.38, 2.65)	**NT-RFA**	NA	1.01 (0.39, 2.59)	NA	NA	NA	NA	NA
0.95 (0.27, 3.40)	0.76 (0.22, 2.59)	0.92 (0.40, 2.14)	0.90 (0.43, 1.87)	0.90 (0.28, 2.89)	**PEI**	1.05 (0.52, 2.14)	NA	NA	2.33 (0.12, 43.65)	0.50 (0.03, 9.87)	NA
1.07 (0.37, 3.12)	0.86 (0.31, 2.35)	1.04 (0.64, 1.68)	1.01 (0.78, 1.31)	1.01 (0.39, 2.59)	1.12 (0.56, 2.25)	**RFA**	1.07 (0.84, 1.35)	NA	**0.65 (0.48, 0.87)**	NA	1.07 (0.07, 16.57)
1.14 (0.38, 3.42)	0.92 (0.33, 2.58)	1.11 (0.65, 1.89)	1.08 (0.76, 1.53)	1.08 (0.41, 2.85)	1.20 (0.58, 2.50)	1.07 (0.84, 1.35)	**RT**	NA	NA	NA	NA
0.27 (0.01, 5.91)	0.22 (0.01, 4.64)	0.26 (0.01, 4.92)	0.25 (0.01, 4.65)	0.26 (0.01, 5.35)	0.28 (0.01, 5.56)	0.25 (0.01, 4.57)	0.24 (0.01, 4.31)	**RT+SR**	2.60 (0.15, 46.18)	NA	NA
0.70 (0.23, 2.13)	0.56 (0.20, 1.61)	0.68 (0.39, 1.19)	**0.66 (0.45, 0.98)**	0.66 (0.25, 1.77)	0.74 (0.35, 1.55)	**0.66 (0.49, 0.88)**	**0.61 (0.42, 0.89)**	2.60 (0.15, 46.18)	**SR**	NA	1.30 (0.91, 1.86)
0.47 (0.02, 12.17)	0.38 (0.02, 9.57)	0.46 (0.02, 10.22)	0.45 (0.02, 9.67)	0.45 (0.02, 11.03)	0.50 (0.03, 9.87)	0.44 (0.02, 9.50)	0.41 (0.02, 8.96)	1.75 (0.03, 118.25)	0.67 (0.03, 14.63)	**TACE+PEI**	NA
0.92 (0.29, 2.93)	0.73 (0.24, 2.22)	0.89 (0.46, 1.72)	0.86 (0.51, 1.45)	0.86 (0.30, 2.46)	0.96 (0.42, 2.19)	0.86 (0.54, 1.35)	0.80 (0.48, 1.34)	3.38 (0.19, 61.50)	1.30 (0.91, 1.86)	1.94 (0.09, 42.97)	**TACE+RFA**

Legend:
- High certainty
- Moderate certainty
- Low certainty
- Very low certainty

Supplementary Figure 1: League table and GRADE certainty of the outcomes ***(continued)***

The left lower cells represent the NMA result between two treatments labelled on the terminals of the columns and the rows, and the right upper cells represent the direct comparison between the corresponding treatments. A: Overall survival (HR and 95% confidence intervals); B: Recurrence-free survival (HR and 95% confidence intervals); C: Complications (RR and 95% confidence intervals); ^{125}I: Iodine-125 seed implantation; CA: Cryoablation; DEM-TACE: Drug-eluting microsphere transcatheter arterial chemoembolization; IRE: Irreversible electroporation; LA: Laser ablation; MWA: Microwave ablation; NT-RFA: No-touch radiofrequency ablation; PEI: Percutaneous ethanol injection; RFA: Radiofrequency ablation; RT: Radiotherapy; SR: Surgical resection; TACE: Transcatheter arterial chemoembolization.

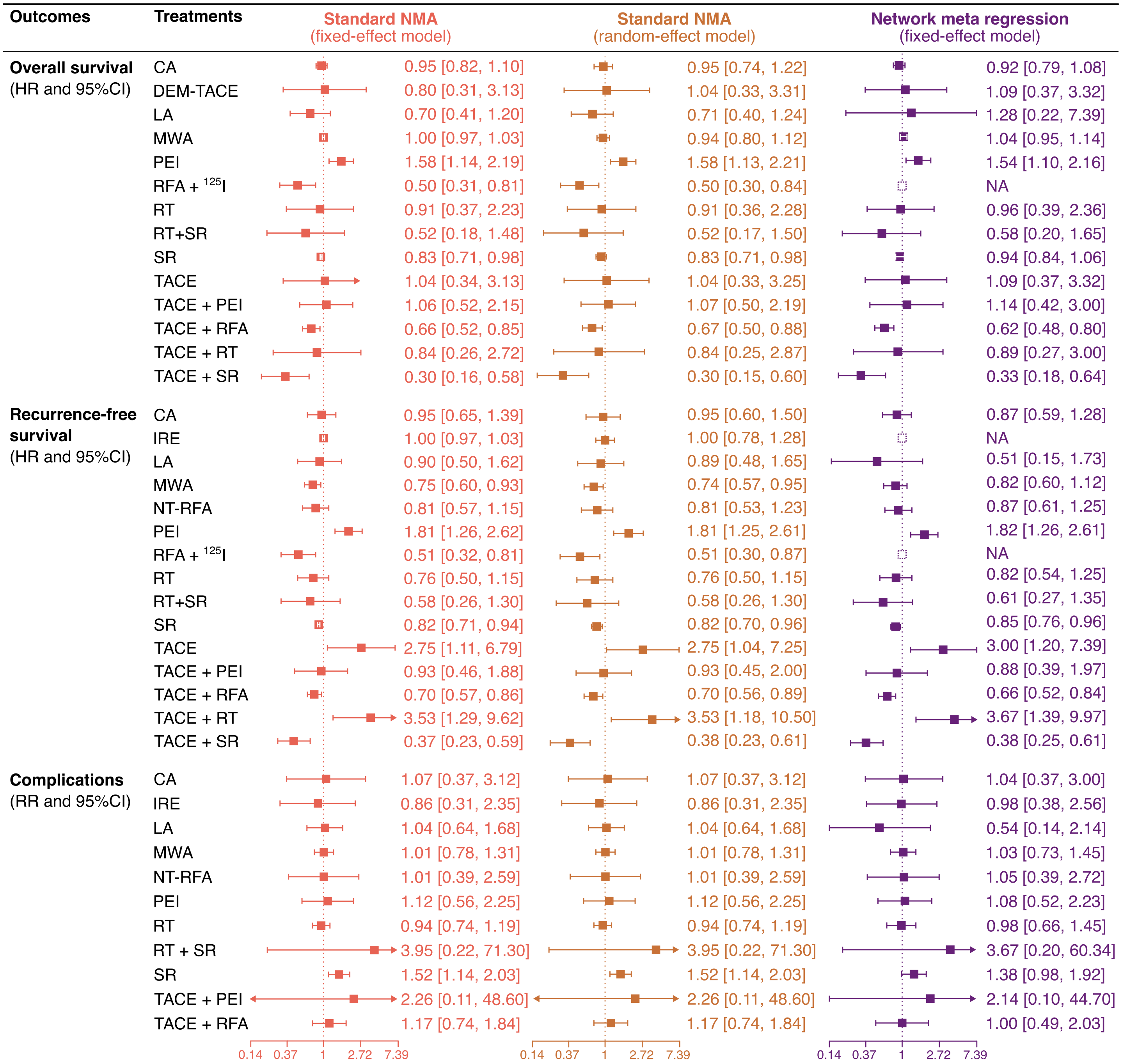

Supplementary Figure 2: Forest plots for standard network meta analysis (fixed-effect model) and network meta regression (fixed effect model)

RFA was set as reference; results of standard network meta analysis (fixed-effect model) were also reported in panel C of Figures 2, 3, and 4; NMA: Network meta analysis; ^{125}I: Iodine-125 seed implantation; CA: Cryoablation; DEM-TACE: Drug-eluting microsphere transcatheter arterial chemoembolization; IRE: Irreversible electroporation; LA: Laser ablation; MWA: Microwave ablation; NT-RFA: No-touch radiofrequency ablation; PEI: Percutaneous ethanol injection; RFA: Radiofrequency ablation; RT: Radiotherapy; SR: Surgical resection; TACE: Transcatheter arterial chemoembolization.

	Treatment1	Treatment2	D1	D2	D3	D4	D5	Overall
**Abdelaziz2014**	MWA	RFA	!	+	−	+	!	−
**Brunello2008**	RFA	PEI	+	+	+	+	+	+
**Bush2023**	RT	TACE	+	!	+	+	+	!
**Chen2006**	RFA	SR	+	!	+	+	+	!
**Chen2014**	RFA + ^{125}I	RFA	+	+	+	+	+	+
**Chong2020**	MWA	RFA	+	+	+	+	+	+
**Costanzo2015**	RFA	LA	+	+	+	+	+	+
**Fang2014**	SR	RFA	−	+	+	+	!	−
**Fang2023**	TACE + SR	SR	+	+	+	+	+	+
**Feng2012**	SR	RFA	+	+	+	+	+	+
**Féray2023**	TACE	TACE + RT	+	+	+	+	+	+
**Ferrari2007**	RFA	LA	+	+	+	+	+	+
**Giorgio2011**	PEI	RFA	+	+	−	!	+	−
**Gjoreski2021**	TACE	DEM-TACE	+	!	+	+	!	!
**Huang2005**	PEI	SR	+	+	+	+	+	+
**Huang2010**	RFA	SR	+	+	+	+	+	+
**Kamal2019**	MWA	RFA	+	+	+	+	!	!
**Koda2001**	PEI + TACE	PEI	+	+	+	+	+	+
**Lee2018**	SR	RFA	+	+	+	+	+	+
**Lencioni2003**	RFA	PEI	+	+	+	+	+	+
**Lin2005**	RFA	PEI	+	+	+	+	+	+
**Liu2016**	TACE + RFA	SR	+	+	+	+	+	+
**Mizuki2010**	TACE	PEI	+	+	+	+	+	+
**Ng2017**	SR	RFA	+	+	+	+	+	+
**Orlacchio**	LA	RFA	+	+	+	+	!	!
**Park2021**	RFA	NT-RFA	+	+	+	+	+	+
**Shibata2002**	RFA	MWA	+	+	+	+	+	+
**Shibata2009**	TACE + RFA	RFA	−	+	+	+	!	−
**Song2024**	SR	RFA	+	+	+	+	+	+
**Sugimoto2025**	RFA	MWA	+	+	+	+	+	+
**Suh2021**	RFA	NT-RFA	+	+	+	+	+	+
**Takayama2022**	SR	RFA	+	+	+	+	+	+
**Vietti2018**	MWA	RFA	+	!	+	+	+	!
**Vogl2024**	MWA	RFA	+	+	+	+	+	+
**Wang2015**	CA	RFA	+	+	+	+	+	+
**Wei2023**	RT	SR	+	+	+	+	+	+
**Xi2024**	MWA	RFA	+	+	+	+	+	+
**Yu2017**	RT	RFA	+	+	+	+	+	+
**Zhang2021**	TACE + RFA	RFA	+	+	+	+	+	+
**Zhang2022**	IRE	RFA	+	+	+	+	+	+
**Zhang2025**	TACE + RFA	SR	+	+	+	+	+	+

\+ Low   ! Some concerns   − High

Supplementary Figure 3: RoB 2.0 assessment of the randomized controlled trials included in this study

D1: Bias arising from the randomization process; D2: Bias due to deviations from intended interventions; D3: Bias due to missing outcome data; D4: Bias in measurement of the outcome; D5: Bias in selection of the reported result; ^{125}I: Iodine-125 seed implantation; CA: Cryoablation; DEM-TACE: Drug-eluting microsphere transcatheter arterial chemoembolization; IRE: Irreversible electroporation; LA: Laser ablation; MWA: Microwave ablation; NT-RFA: No-touch radiofrequency ablation; PEI: Percutaneous ethanol injection; RFA: Radiofrequency ablation; RT: Radiotherapy; SR: Surgical resection; TACE: Transcatheter arterial chemoembolization.

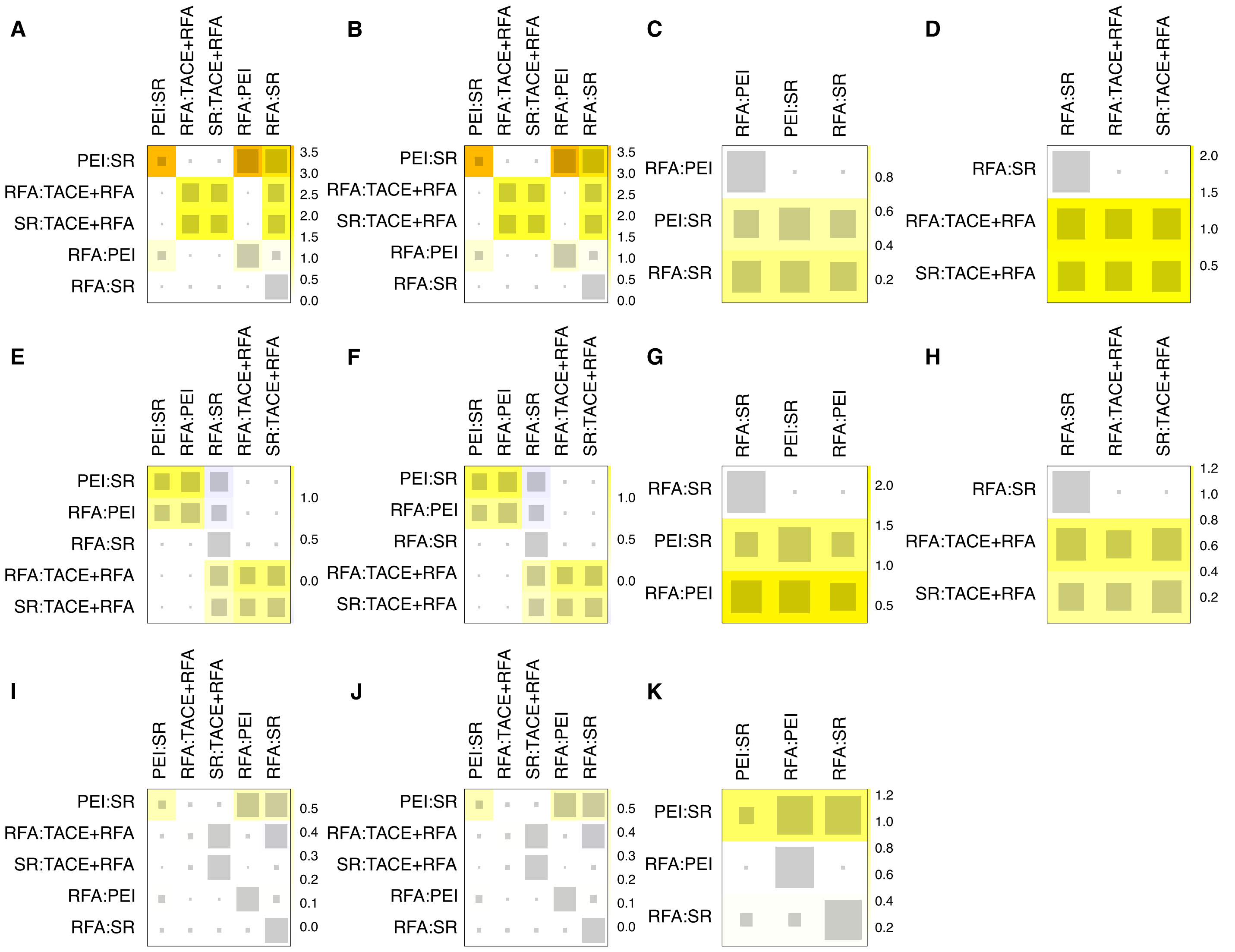

Supplementary Figure 4: The inconsistency of the standard network meta analysis

The size of the square represents the weight of the comparison in the overall comparison; and the background color of the square represents the degrees of inconsistency and heterogeneity. The colour scale is shown on the right. A $\sim$ D: Overall survival; E $\sim$ H: Recurrence-free survival; I $\sim$ K: Complications; A, E, I: Standard network meta analysis with fixed-effect model; B, F, J: Standard network meta analysis with random-effect model; C, G, K: Network meta analysis in subgroup with tumor size $\leqslant$ 3cm; D, H: Network meta analysis in subgroup with tumor size $>$ 3cm. Subgroup analysis for complications in subgroup with tumor size $>$ 3cm is not available because no loop exists in the network. PEI: Percutaneous ethanol injection; RFA: Radiofrequency ablation; SR: Surgical resection; TACE: Transcatheter arterial chemoembolization.

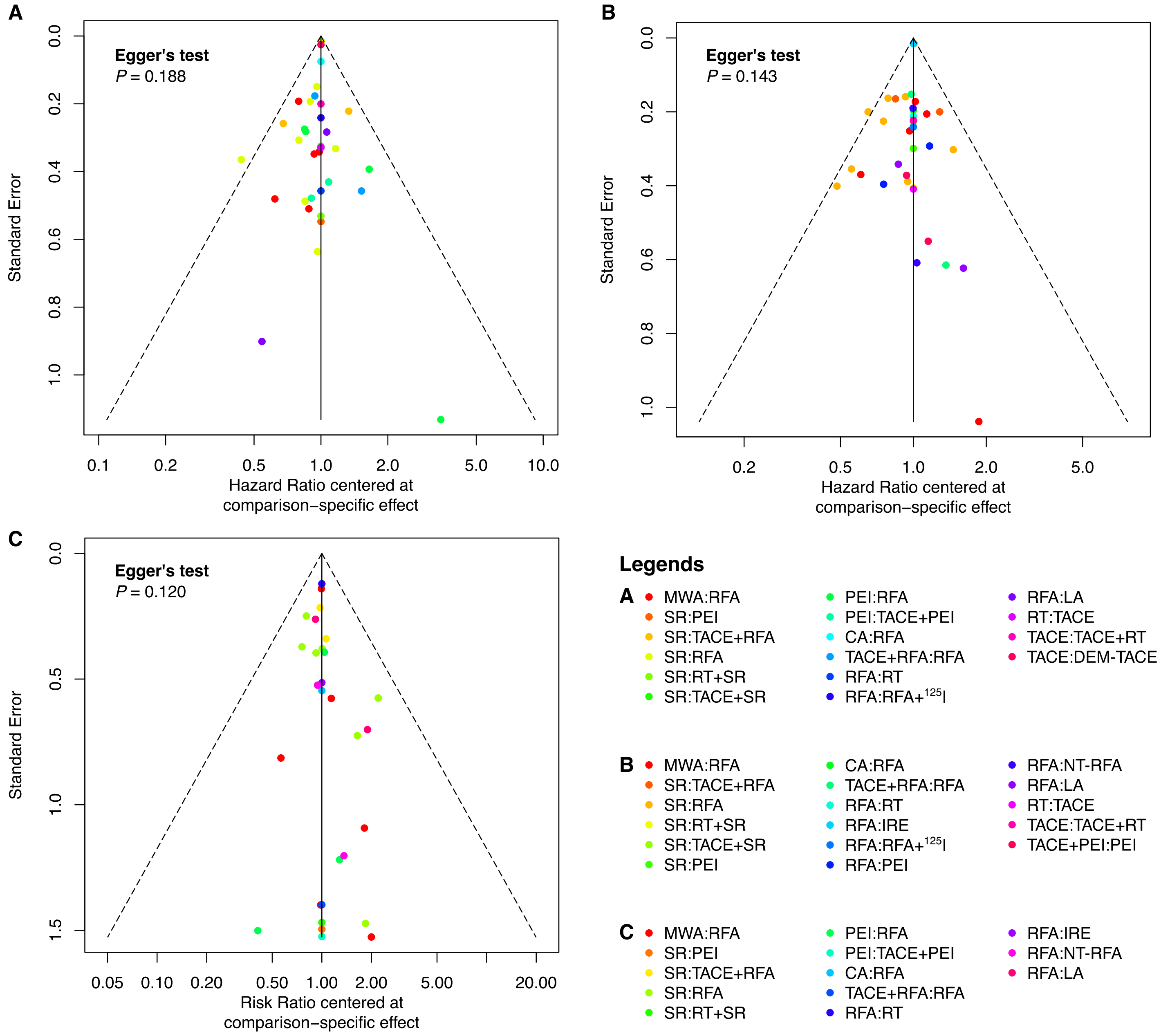

Supplementary Figure 5: Funnel plots of the outcomes

A: The funnel plots and Egger's test of overall survival; B: The funnel plots and Egger's test of recurrence-free survival; C: The funnel plots and Egger's test of complications; ^{125}I: Iodine-125 seed implantation; CA: Cryoablation; DEM-TACE: Drug-eluting microsphere transcatheter arterial chemoembolization; IRE: Irreversible electroporation; LA: Laser ablation; MWA: Microwave ablation; NT-RFA: No-touch radiofrequency ablation; PEI: Percutaneous ethanol injection; RFA: Radiofrequency ablation; RT: Radiotherapy; SR: Surgical resection; TACE: Transcatheter arterial chemoembolization.

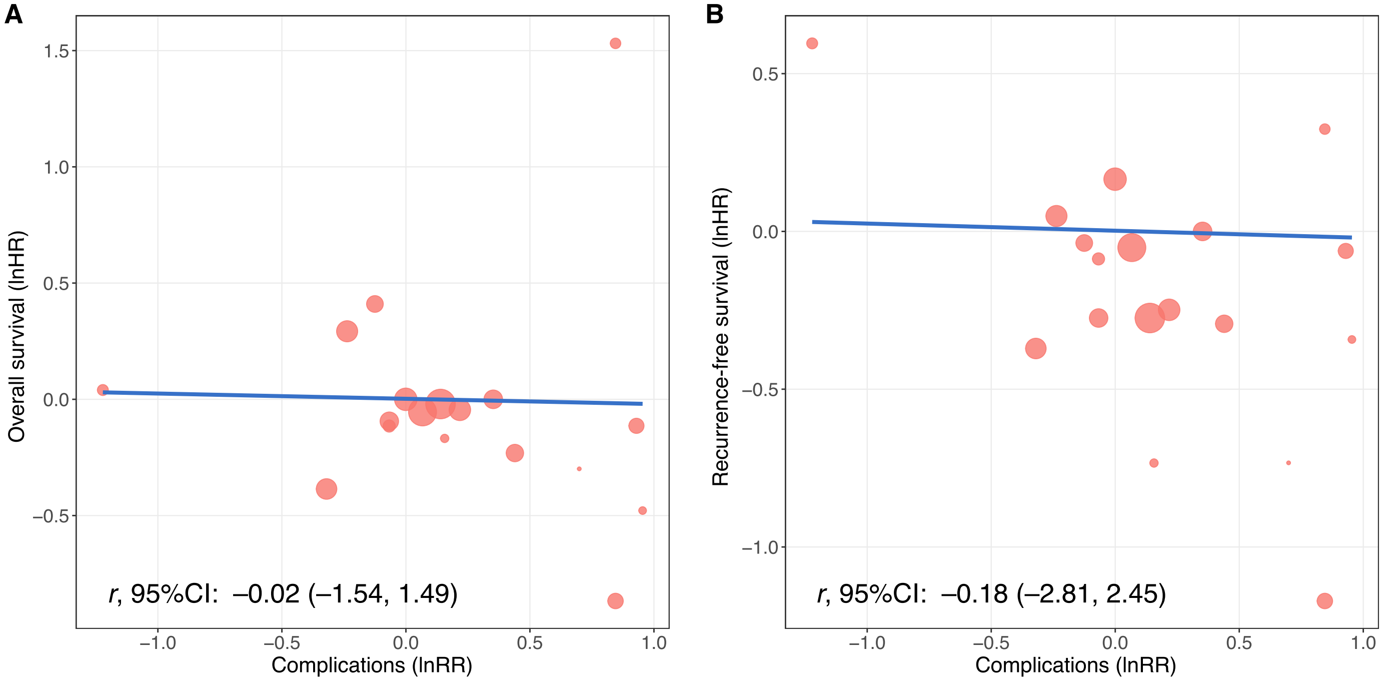

Supplementary Figure 6: Correlation analysis between overall survival or recurrence-free survival (lnHR) and complications (lnRR) at study levels

A: Correlation analysis between overall survival (lnHR) and complications (lnRR) at study levels; B: Correlation analysis between recurrence-free survival (lnHR) and complications (lnRR) at study levels

2 Supplementary Tables

Supplementary Table 1: Treatments included in this study for small hepatocellular carcinoma

Treatment	Abbreviation	Biological rationale
^{125}I	^{125}I	Implanting ^{125}I emitting low-dose γ-rays into the tumor, damaging DNA of tumor cells and inducing cell death.
Cryoablation	CA	Argon-induced rapid freezing creates destructive intracellular ice crystals, followed by helium-mediated thawing. This extreme freeze-thaw cycle causes irreversible cellular damage and ischemic tumor necrosis.
Drug-eluting microsphere transcatheter arterial chemoembolization	DEM-TACE	Hepatic arterial delivery of drug-eluting microspheres induces synergistic ischemic and cytotoxic tumor cell death via targeted vessel embolization and sustained chemotherapy release.
Irreversible electroporation	IRE	High-voltage direct current pulses create lethal nanopores in the cell membrane, causing loss of osmotic balance and apoptosis.
Laser ablation	LA	High-energy laser light is absorbed by tumor tissues and converted into thermal energy, causing highly precise coagulative necrosis.
Microwave ablation	MWA	Electromagnetic waves cause polar molecules (like water) to vibrate rapidly, generating high temperature to kill tumor cells.
No-touch radiofrequency ablation	NT-RFA	Electrodes are placed in normal tissue around the tumor, causing centripetal (outside-in) heating that kills tumor cells.
Percutaneous ethanol injection	PEI	Direct injection of absolute ethanol into the tumor causes rapid dehydration, protein coagulation, and chemical necrosis.
Radiofrequency ablation	RFA	High-frequency alternating current causes ionic agitation and frictional heating, generating high temperature to kill tumor cells.
Radiotherapy	RT	High-energy external radiation beams precisely target the tumor, causing severe DNA damage and apoptosis.
Surgical resection	SR	Physical removal of the tumor and a surrounding margin of normal tissue to completely eliminate the tumor burden and prevent metastasis.
Transcatheter arterial chemoembolization	TACE	Transcatheter hepatic arterial delivery of embolic agents and chemotherapy drugs induces tumor death via synergistic ischemic necrosis and cytotoxicity.

Supplementary Table 2: Incremental effects of the treatment components

Treatments	Overall survival		Recurrence-free survival		Complications	
	**iHR (95% CI)**	***P***	**iHR (95% CI)**	***P***	**iRR (95% CI)**	***P***
^{125}I	0.50 (0.31, 0.81)	0.004	0.51 (0.32, 0.81)	0.005	NA	NA
CA	0.75 (0.29, 1.97)	0.564	1.00 (0.52, 1.95)	0.993	2.97 (0.14, 64.60)	0.489
DEM-TACE	0.64 (0.52, 0.79)	< 0.001	NA	NA	NA	NA
IRE	NA	NA	1.06 (0.61, 1.82)	0.844	2.38 (0.11, 50.71)	0.579
LA	0.56 (0.19, 1.67)	0.297	0.95 (0.43, 2.11)	0.899	2.88 (0.15, 53.78)	0.479
MWA	0.79 (0.31, 2.06)	0.633	0.79 (0.44, 1.41)	0.424	2.80 (0.15, 50.80)	0.487
NT-RFA	NA	NA	0.85 (0.45, 1.63)	0.630	2.80 (0.13, 58.40)	0.507
PEI	1.26 (0.46, 3.43)	0.658	1.91 (0.99, 3.68)	0.053	3.12 (0.16, 60.87)	0.452
RFA	0.79 (0.31, 2.06)	0.636	1.06 (0.61, 1.82)	0.844	2.78 (0.15, 49.86)	0.488
RT	0.72 (0.52, 1.00)	0.049	0.80 (0.57, 1.14)	0.215	2.60 (0.15, 46.18)	0.516
SR	0.66 (0.25, 1.73)	0.396	0.86 (0.49, 1.51)	0.593	4.24 (0.23, 77.34)	0.329
TACE	0.64 (0.52, 0.78)	< 0.001	0.68 (0.57, 0.81)	< 0.001	1.18 (0.75, 1.85)	0.465
RFA + ^{125}I	0.40 (0.14, 1.16)	0.092	0.54 (0.26, 1.10)	0.090	NA	NA
RT + SR	0.48 (0.16, 1.46)	0.193	0.69 (0.30, 1.57)	0.376	11.03 (0.03, 3529.49)	0.415
TACE + PEI	0.80 (0.28, 2.26)	0.676	1.31 (0.65, 2.61)	0.448	3.69 (0.18, 74.55)	0.394
TACE + RFA	0.51 (0.19, 1.36)	0.177	0.72 (0.40, 1.29)	0.271	3.28 (0.18, 61.04)	0.425
TACE + RT	0.46 (0.31, 0.70)	< 0.001	0.55 (0.37, 0.82)	0.004	NA	NA
TACE + SR	0.42 (0.15, 1.15)	0.092	0.59 (0.32, 1.08)	0.088	NA	NA

^{125}I: Iodine-125 seed implantation; CA: Cryoablation; DEM-TACE: Drug-eluting microsphere transcatheter arterial chemoembolization; iHR: incremental hazard ratio; iRR: incremental risk ratio; IRE: Irreversible electroporation; LA: Laser ablation; MWA: Microwave ablation; NT-RFA: No-touch radiofrequency ablation; PEI: Percutaneous ethanol injection; RFA: Radiofrequency ablation; RT: Radiotherapy; SR: Surgical resection; TACE: Transcatheter arterial chemoembolization.

Supplementary Table 3: Overall heterogeneity of the outcomes

Outcomes	Model		Q	df	I^2 (%)
Overall Survival	Standard NMA (fixed-effect model)		25.48	21	17.58
	Standard NMA (random-effect model)		25.48	21	17.58
	Additive NMA		30.34	25	17.60
	Subgroup analysis	$\leqslant$ 3cm	3.09	3	2.91
		$>$ 3cm	17.40	14	19.54
Recurrence-free Survival	Standard NMA (fixed-effect model)		27.80	20	28.06
	Standard NMA (random-effect model)		27.80	20	28.06
	Additive NMA		49.98	24	51.98
	Subgroup analysis	$\leqslant$ 3cm	5.61	3	46.52
		$>$ 3cm	21.25	12	43.53
Complications	Standard NMA (fixed-effect model)		8.92	19	0.00
	Standard NMA (random-effect model)		8.92	19	0.00
	Additive NMA		9.05	20	0.00
	Subgroup analysis	$>$ 3cm	1.99	5	39.89
		$\leqslant$ 3cm	5.36	11	0.00

NMA: Network meta analysis

Supplementary Table 4: Network Meta-regression for the outcomes

Outcomes	Regression coefficient (Mean and 95%CI)	I^2 (%)
Overall Survival	-0.09 $(-0.27, 0.09)$	0.15
Recurrence-free Survival	-0.16 $(-0.28, -0.04)$	0.00
Complications	-0.08 $(-0.62, 0.46)$	0.00

Supplementary Table 5: GRADE assessment of the comparisons according to the NMA result

Comparisons	No. of studies	Within-study bias	Reporting bias	Indirectness	Imprecision	Heterogeneity	Incoherence	Confidence rating
**Overall Survival**								
CA:RFA	1	–	–	–	↓	–	–	Moderate
DEM-TACE:TACE	1	↓	–	–	↓	–	–	Low
LA:RFA	2	–	–	–	↓	–	–	Moderate
MWA:RFA	6	–	–	–	↓	–	–	Moderate
PEI:RFA	4	↓	–	–	–	–	↓	Low
PEI:SR	1	–	–	–	–	–	↓	Moderate
PEI:TACE+PEI	2	–	–	–	↓	–	–	Moderate
RFA:RFA+^{125}I	1	–	–	–	–	–	–	High
RFA:RT	1	–	–	–	↓↓	–	–	Low
RFA:SR	8	–	–	–	–	–	–	High
RFA:TACE+RFA	2	–	–	–	–	↓	–	Moderate
RT:TACE	1	↓	–	–	↓↓	–	–	Very low
RT+SR:SR	1	–	–	–	↓↓	–	–	Low
SR:TACE+RFA	2	–	–	–	–	–	–	High
SR:TACE+SR	1	–	–	–	–	–	–	High
TACE:TACE+RT	1	–	–	–	↓	–	–	Moderate
CA:DEM-TACE	0	↓	–	–	↓↓	–	–	Very low
CA:LA	0	–	–	–	↓↓	–	–	Low
CA:MWA	0	–	–	–	↓	–	–	Moderate
CA:PEI	0	–	–	–	–	–	–	High
CA:RFA+^{125}I	0	–	–	–	–	↓	–	Moderate
CA:RT	0	–	–	–	↓↓	–	–	Low
CA:RT+SR	0	–	–	–	↓↓	–	–	Low
CA:SR	0	–	–	–	↓	–	–	Moderate
CA:TACE	0	–	–	–	↓↓	–	–	Low
CA:TACE+PEI	0	–	–	–	↓↓	–	–	Low
CA:TACE+RFA	0	–	–	–	–	–	–	High
CA:TACE+RT	0	–	–	–	↓↓	–	–	Low
CA:TACE+SR	0	–	–	–	–	–	–	High
DEM-TACE:LA	0	↓	–	–	↓↓	–	–	Very low
DEM-TACE:MWA	0	↓	–	–	↓↓	–	–	Very low
DEM-TACE:PEI	0	↓	–	–	↓↓	–	–	Very low
DEM-TACE:RFA	0	↓	–	–	↓↓	–	–	Very low
DEM-TACE:RFA+^{125}I	0	↓	–	–	↓↓	–	–	Very low
DEM-TACE:RT	0	↓	–	–	↓↓	–	–	Very low
DEM-TACE:RT+SR	0	–	–	–	↓↓	–	–	Low
DEM-TACE:SR	0	↓	–	–	↓↓	–	–	Very low
DEM-TACE:TACE+PEI	0	↓	–	–	↓↓	–	–	Very low
DEM-TACE:TACE+RFA	0	↓	–	–	↓↓	–	–	Very low
DEM-TACE:TACE+RT	0	↓	–	–	↓	–	–	Moderate
DEM-TACE:TACE+SR	0	–	–	–	↓	↓	–	Low
LA:MWA	0	–	–	–	↓↓	–	–	Low
LA:PEI	0	–	–	–	–	–	–	High
LA:RFA+^{125}I	0	–	–	–	↓↓	–	–	Low
LA:RT	0	–	–	–	↓↓	–	–	Low

Supplementary Table 5: GRADE assessment of the comparisons according to the NMA result *(continued)*

Comparisons	No. of studies	Within-study bias	Reporting bias	Indirectness	Imprecision	Heterogeneity	Incoherence	Confidence rating
LA:RT+SR	0	–	–	–	↓↓	–	–	Low
LA:SR	0	–	–	–	↓↓	–	–	Low
LA:TACE	0	–	–	–	↓↓	–	–	Low
LA:TACE+PEI	0	–	–	–	↓↓	–	–	Low
LA:TACE+RFA	0	–	–	–	↓↓	–	–	Low
LA:TACE+RT	0	–	–	–	↓↓	–	–	Low
LA:TACE+SR	0	–	–	–	↓	–	–	Moderate
MWA:PEI	0	–	–	–	–	–	–	High
MWA:RFA+^{125}I	0	–	–	–	–	–	–	High
MWA:RT	0	–	–	–	↓↓	–	–	Low
MWA:RT+SR	0	–	–	–	↓↓	–	–	Low
MWA:SR	0	–	–	–	↓	↓	–	Low
MWA:TACE	0	–	–	–	↓↓	–	–	Low
MWA:TACE+PEI	0	–	–	–	↓↓	–	–	Low
MWA:TACE+RFA	0	–	–	–	–	–	–	High
MWA:TACE+RT	0	–	–	–	↓↓	–	–	Low
MWA:TACE+SR	0	–	–	–	–	–	–	High
PEI:RFA+^{125}I	0	–	–	–	–	–	–	High
PEI:RT	0	–	–	–	↓↓	–	–	Low
PEI:RT+SR	0	–	–	–	↓	–	–	Moderate
PEI:TACE	0	↓	–	–	↓↓	–	–	Very low
PEI:TACE+RFA	0	–	–	–	–	–	–	High
PEI:TACE+RT	0	–	–	–	↓↓	–	–	Low
PEI:TACE+SR	0	–	–	–	–	–	–	High
RFA:RT+SR	0	–	–	–	↓↓	–	–	Low
RFA:TACE	0	↓	–	–	↓↓	–	–	Very low
RFA:TACE+PEI	0	–	–	–	↓↓	–	–	Low
RFA:TACE+RT	0	–	–	–	↓↓	–	–	Low
RFA:TACE+SR	0	–	–	–	–	–	–	High
RFA+^{125}I:RT	0	–	–	–	↓↓	–	–	Low
RFA+^{125}I:RT+SR	0	–	–	–	↓↓	–	–	Low
RFA+^{125}I:SR	0	–	–	–	–	–	–	Low
RFA+^{125}I:TACE	0	–	–	–	↓↓	–	–	Low
RFA+^{125}I:TACE+PEI	0	–	–	–	↓	–	–	Moderate
RFA+^{125}I:TACE+RFA	0	–	–	–	↓	–	–	Moderate
RFA+^{125}I:TACE+RT	0	–	–	–	↓↓	–	–	Low
RFA+^{125}I:TACE+SR	0	–	–	–	↓↓	–	–	Low
RT:RT+SR	0	–	–	–	↓↓	–	–	Low
RT:SR	0	–	–	–	↓↓	–	–	Low
RT:TACE+PEI	0	–	–	–	↓↓	–	–	Low
RT:TACE+RFA	0	–	–	–	↓↓	–	–	Low
RT:TACE+RT	0	↓	–	–	↓↓	–	–	Very low
RT:TACE+SR	0	–	–	–	↓	↓	–	Low
RT+SR:TACE	0	–	–	–	↓↓	–	–	Low
RT+SR:TACE+PEI	0	–	–	–	↓↓	–	–	Low
RT+SR:TACE+RFA	0	–	–	–	↓↓	–	–	Low

Supplementary Table 5: GRADE assessment of the comparisons according to the NMA result *(continued)*

Comparisons	No. of studies	Within-study bias	Reporting bias	Indirect-ness	Impre-cision	Hetero-geneity	Incohe-rence	Confiden-ce rating
RT+SR:TACE+RT	0	–	–	–	↓↓	–	–	Low
RT+SR:TACE+SR	0	–	–	–	↓↓	–	–	Low
SR:TACE	0	–	–	–	↓↓	–	–	Low
SR:TACE+PEI	0	–	–	–	↓↓	–	–	Low
SR:TACE+RT	0	–	–	–	↓↓	–	–	Low
TACE:TACE+PEI	0	–	–	–	↓↓	–	–	Low
TACE:TACE+RFA	0	–	–	–	↓↓	–	–	Low
TACE:TACE+SR	0	–	–	–	↓	↓	–	Low
TACE+PEI:TACE+RFA	0	–	–	–	↓↓	–	–	Low
TACE+PEI:TACE+RT	0	–	–	–	↓↓	–	–	Low
TACE+PEI:TACE+SR	0	–	–	–	–	–	–	High
TACE+RFA:TACE+RT	0	–	–	–	↓↓	–	–	Low
TACE+RFA:TACE+SR	0	–	–	–	–	↓	–	Moderate
TACE+RT:TACE+SR	0	–	–	–	↓↓	–	–	Low
**Recurrence-free Survival**								
CA:RFA	1	–	–	–	↓↓	–	–	Low
IRE:RFA	1	–	–	–	↓	–	–	Moderate
LA:RFA	2	–	–	–	↓↓	–	–	Low
MWA:RFA	5	–	–	–	–	–	–	High
NT-RFA:RFA	2	–	–	–	↓	–	–	Moderate
PEI:RFA	2	–	–	–	–	–	↓	Moderate
PEI:SR	1	–	–	–	–	–	↓	Moderate
PEI:TACE+PEI	2	–	–	–	–	–	–	High
RFA:RFA+^{125}I	1	–	–	–	–	–	–	High
RFA:RT	1	–	–	–	↓	–	–	Moderate
RFA:SR	9	–	–	–	–	–	–	High
RFA:TACE+RFA	2	–	–	–	–	–	–	High
RT:TACE	1	↓	–	–	–	–	–	Moderate
RT+SR:SR	1	–	–	–	↓↓	–	–	Low
SR:TACE+RFA	2	–	–	–	↓	–	–	Moderate
SR:TACE+SR	1	–	–	–	–	–	–	High
TACE:TACE+RT	1	–	–	–	↓↓	–	–	Low
CA:IRE	0	–	–	–	↓↓	–	–	Low
CA:LA	0	–	–	–	↓↓	–	–	Low
CA:MWA	0	–	–	–	↓↓	–	–	Low
CA:NT-RFA	0	–	–	–	↓↓	–	–	Low
CA:PEI	0	–	–	–	–	–	–	High
CA:RFA+^{125}I	0	–	–	–	–	–	–	High
CA:RT	0	–	–	–	↓↓	–	–	Low
CA:RT+SR	0	–	–	–	↓↓	–	–	Low
CA:SR	0	–	–	–	↓↓	–	–	Low
CA:TACE	0	–	–	–	–	–	–	High
CA:TACE+PEI	0	–	–	–	↓↓	–	–	Low
CA:TACE+RFA	0	–	–	–	↓↓	–	–	Low
CA:TACE+RT	0	–	–	–	–	–	–	High
CA:TACE+SR	0	–	–	–	–	–	–	High

Supplementary Table 5: **GRADE assessment of the comparisons according to the NMA result** *(continued)*

Comparisons	No. of studies	Within-study bias	Reporting bias	Indirectness	Imprecision	Heterogeneity	Incoherence	Confidence rating
IRE:LA	0	–	–	–	↓↓	–	–	Low
IRE:MWA	0	–	–	–	–	–	–	High
IRE:NT-RFA	0	–	–	–	↓	–	–	Moderate
IRE:PEI	0	–	–	–	–	–	–	High
IRE:RFA+^{125}I	0	–	–	–	–	–	–	High
IRE:RT	0	–	–	–	↓	–	–	Moderate
IRE:RT+SR	0	–	–	–	↓↓	–	–	Low
IRE:SR	0	–	–	–	–	–	–	High
IRE:TACE	0	–	–	–	–	–	–	High
IRE:TACE+PEI	0	–	–	–	↓↓	–	–	Low
IRE:TACE+RFA	0	–	–	–	–	–	–	High
IRE:TACE+RT	0	–	–	–	–	–	–	High
IRE:TACE+SR	0	–	–	–	–	–	–	High
LA:MWA	0	–	–	–	↓↓	–	–	Low
LA:NT-RFA	0	–	–	–	↓↓	–	–	Low
LA:PEI	0	–	–	–	–	–	–	High
LA:RFA+^{125}I	0	–	–	–	↓	–	–	Moderate
LA:RT	0	–	–	–	↓↓	–	–	Low
LA:RT+SR	0	–	–	–	↓↓	–	–	Low
LA:SR	0	–	–	–	↓↓	–	–	Low
LA:TACE	0	–	–	–	–	–	–	High
LA:TACE+PEI	0	–	–	–	↓↓	–	–	Low
LA:TACE+RFA	0	–	–	–	↓↓	–	–	Low
LA:TACE+RT	0	–	–	–	–	–	–	High
LA:TACE+SR	0	–	–	–	–	–	–	High
MWA:NT-RFA	0	–	–	–	↓↓	–	–	Low
MWA:PEI	0	–	–	–	–	–	–	High
MWA:RFA+^{125}I	0	–	–	–	↓	–	–	Moderate
MWA:RT	0	–	–	–	↓↓	–	–	Low
MWA:RT+SR	0	–	–	–	↓↓	–	–	Low
MWA:SR	0	–	–	–	↓	–	–	Moderate
MWA:TACE	0	–	–	–	–	–	–	High
MWA:TACE+PEI	0	–	–	–	↓↓	–	–	Low
MWA:TACE+RFA	0	–	–	–	↓↓	–	–	Low
MWA:TACE+RT	0	–	–	–	–	–	–	High
MWA:TACE+SR	0	–	–	–	–	↓	–	Moderate
NT-RFA:PEI	0	–	–	–	–	–	–	High
NT-RFA:RFA+^{125}I	0	–	–	–	↓	–	–	Moderate
NT-RFA:RT	0	–	–	–	↓↓	–	–	Low
NT-RFA:RT+SR	0	–	–	–	↓↓	–	–	Low
NT-RFA:SR	0	–	–	–	↓	–	–	Moderate
NT-RFA:TACE	0	–	–	–	–	–	–	High
NT-RFA:TACE+PEI	0	–	–	–	↓↓	–	–	Low
NT-RFA:TACE+RFA	0	–	–	–	↓↓	–	–	Low
NT-RFA:TACE+RT	0	–	–	–	–	–	–	High
NT-RFA:TACE+SR	0	–	–	–	–	↓	–	Moderate

Supplementary Table 5: GRADE assessment of the comparisons according to the NMA result *(continued)*

Comparisons	No. of studies	Within-study bias	Reporting bias	Indirectness	Imprecision	Heterogeneity	Incoherence	Confidence rating
PEI:RFA+^{125}I	0	–	–	–	–	–	–	High
PEI:RT	0	–	–	–	–	–	–	High
PEI:RT+SR	0	–	–	–	–	–	–	High
PEI:TACE	0	–	–	–	↓↓	–	–	Low
PEI:TACE+RFA	0	–	–	–	–	–	–	High
PEI:TACE+RT	0	–	–	–	↓↓	–	–	Low
PEI:TACE+SR	0	–	–	–	–	–	–	High
RFA:RT+SR	0	–	–	–	↓↓	–	–	Low
RFA:TACE	0	↓	–	–	–	–	–	Moderate
RFA:TACE+PEI	0	–	–	–	↓↓	–	–	Low
RFA:TACE+RT	0	–	–	–	–	–	–	High
RFA:TACE+SR	0	–	–	–	–	–	–	High
RFA+^{125}I:RT	0	–	–	–	↓↓	–	–	Low
RFA+^{125}I:RT+SR	0	–	–	–	↓↓	–	–	Low
RFA+^{125}I:SR	0	–	–	–	↓	–	–	Moderate
RFA+^{125}I:TACE	0	–	–	–	–	–	–	High
RFA+^{125}I:TACE+PEI	0	–	–	–	↓	–	–	Moderate
RFA+^{125}I:TACE+RFA	0	–	–	–	↓	–	–	Moderate
RFA+^{125}I:TACE+RT	0	–	–	–	–	–	–	High
RFA+^{125}I:TACE+SR	0	–	–	–	↓↓	–	–	Low
RT:RT+SR	0	–	–	–	↓↓	–	–	Low
RT:SR	0	–	–	–	↓	–	–	Moderate
RT:TACE+PEI	0	–	–	–	↓↓	–	–	Low
RT:TACE+RFA	0	–	–	–	↓↓	–	–	Low
RT:TACE+RT	0	↓	–	–	–	–	–	Moderate
RT:TACE+SR	0	–	–	–	↓	–	–	Moderate
RT+SR:TACE	0	–	–	–	–	–	–	High
RT+SR:TACE+PEI	0	–	–	–	↓↓	–	–	Low
RT+SR:TACE+RFA	0	–	–	–	↓↓	–	–	Low
RT+SR:TACE+RT	0	–	–	–	–	–	–	High
RT+SR:TACE+SR	0	–	–	–	↓↓	–	–	Low
SR:TACE	0	–	–	–	–	–	–	High
SR:TACE+PEI	0	–	–	–	↓↓	–	–	Low
SR:TACE+RT	0	–	–	–	–	–	–	High
TACE:TACE+PEI	0	–	–	–	↓	–	–	Moderate
TACE:TACE+RFA	0	–	–	–	–	–	–	High
TACE:TACE+SR	0	–	–	–	–	–	–	High
TACE+PEI:TACE+RFA	0	–	–	–	↓↓	–	–	Low
TACE+PEI:TACE+RT	0	–	–	–	–	–	–	High
TACE+PEI:TACE+SR	0	–	–	–	↓	–	–	Moderate
TACE+RFA:TACE+RT	0	–	–	–	–	–	–	High
TACE+RFA:TACE+SR	0	–	–	–	–	–	–	High
TACE+RT:TACE+SR	0	–	–	–	–	–	–	High
**Complications**								
CA:RFA	1	–	–	–	↓↓	–	–	Low
IRE:RFA	1	–	–	–	↓↓	–	–	Low

Supplementary Table 5: GRADE assessment of the comparisons according to the NMA result *(continued)*

Comparisons	No. of studies	Within-study bias	Reporting bias	Indirectness	Imprecision	Heterogeneity	Incoherence	Confidence rating
LA:RFA	2	–	–	–	↓↓	–	–	Low
MWA:RFA	6	–	–	–	↓↓	–	–	Low
NT-RFA:RFA	2	–	–	–	↓↓	–	–	Low
PEI:RFA	3	–	–	–	↓↓	–	–	Low
PEI:SR	1	–	–	–	↓↓	–	–	Low
PEI:TACE+PEI	1	–	–	–	↓↓	–	–	Low
RFA:RT	1	–	–	–	↓↓	–	–	Low
RFA:SR	8	–	–	–	–	–	–	High
RFA:TACE+RFA	1	–	–	–	↓↓	–	–	Low
RT+SR:SR	1	–	–	–	↓↓	–	–	Low
SR:TACE+RFA	2	–	–	–	↓	–	–	Moderate
CA:IRE	0	–	–	–	↓↓	–	–	Low
CA:LA	0	–	–	–	↓↓	–	–	Low
CA:MWA	0	–	–	–	↓↓	–	–	Low
CA:NT-RFA	0	–	–	–	↓↓	–	–	Low
CA:PEI	0	–	–	–	↓↓	–	–	Low
CA:RT	0	–	–	–	↓↓	–	–	Low
CA:RT+SR	0	–	–	–	↓↓	–	–	Low
CA:SR	0	–	–	–	↓↓	–	–	Low
CA:TACE+PEI	0	–	–	–	↓↓	–	–	Low
CA:TACE+RFA	0	–	–	–	↓↓	–	–	Low
IRE:LA	0	–	–	–	↓↓	–	–	Low
IRE:MWA	0	–	–	–	↓↓	–	–	Low
IRE:NT-RFA	0	–	–	–	↓↓	–	–	Low
IRE:PEI	0	–	–	–	↓↓	–	–	Low
IRE:RT	0	–	–	–	↓↓	–	–	Low
IRE:RT+SR	0	–	–	–	↓↓	–	–	Low
IRE:SR	0	–	–	–	↓↓	–	–	Low
IRE:TACE+PEI	0	–	–	–	↓↓	–	–	Low
IRE:TACE+RFA	0	–	–	–	↓↓	–	–	Low
LA:MWA	0	–	–	–	↓↓	–	–	Low
LA:NT-RFA	0	–	–	–	↓↓	–	–	Low
LA:PEI	0	–	–	–	↓↓	–	–	Low
LA:RT	0	–	–	–	↓↓	–	–	Low
LA:RT+SR	0	–	–	–	↓↓	–	–	Low
LA:SR	0	–	–	–	↓	↓	–	Low
LA:TACE+PEI	0	–	–	–	↓↓	–	–	Low
LA:TACE+RFA	0	–	–	–	↓↓	–	–	Low
MWA:NT-RFA	0	–	–	–	↓↓	–	–	Low
MWA:PEI	0	–	–	–	↓↓	–	–	Low
MWA:RT	0	–	–	–	↓↓	–	–	Low
MWA:RT+SR	0	–	–	–	↓↓	–	–	Low
MWA:SR	0	–	–	–	–	–	–	High
MWA:TACE+PEI	0	–	–	–	↓↓	–	–	Low
MWA:TACE+RFA	0	–	–	–	↓↓	–	–	Low
NT-RFA:PEI	0	–	–	–	↓↓	–	–	Low

Supplementary Table 5: GRADE assessment of the comparisons according to the NMA result *(continued)*

Comparisons	No. of studies	Within-study bias	Reporting bias	Indirectness	Imprecision	Heterogeneity	Incoherence	Confidence rating
NT-RFA:RT	0	–	–	–	ⓘⓘ	–	–	Low
NT-RFA:RT+SR	0	–	–	–	ⓘⓘ	–	–	Low
NT-RFA:SR	0	–	–	–	ⓘⓘ	–	–	Low
NT-RFA:TACE+PEI	0	–	–	–	ⓘⓘ	–	–	Low
NT-RFA:TACE+RFA	0	–	–	–	ⓘⓘ	–	–	Low
PEI:RT	0	–	–	–	ⓘⓘ	–	–	Low
PEI:RT+SR	0	–	–	–	ⓘⓘ	–	–	Low
PEI:TACE+RFA	0	–	–	–	ⓘⓘ	–	–	Low
RFA:RT+SR	0	–	–	–	ⓘⓘ	–	–	Low
RFA:TACE+PEI	0	–	–	–	ⓘⓘ	–	–	Low
RT:RT+SR	0	–	–	–	ⓘⓘ	–	–	Low
RT:SR	0	–	–	–	–	–	–	High
RT:TACE+PEI	0	–	–	–	ⓘⓘ	–	–	Low
RT:TACE+RFA	0	–	–	–	ⓘⓘ	–	–	Low
RT+SR:TACE+PEI	0	–	–	–	ⓘⓘ	–	–	Low
RT+SR:TACE+RFA	0	–	–	–	ⓘⓘ	–	–	Low
SR:TACE+PEI	0	–	–	–	ⓘⓘ	–	–	Low
TACE+PEI:TACE+RFA	0	–	–	–	ⓘⓘ	–	–	Low

^{125}I: Iodine-125 seed implantation; CA: Cryoablation; DEM-TACE: Drug-eluting microsphere transcatheter arterial chemoembolization; IRE: Irreversible electroporation; LA: Laser ablation; MWA: Microwave ablation; NMA: Network meta analysis; NT-RFA: No-touch radiofrequency ablation; PEI: Percutaneous ethanol injection; RFA: Radiofrequency ablation; RT: Radiotherapy; SR: Surgical resection; TACE: Transcatheter arterial chemoembolization.

Appendix 1: Search Strategy

PubMed

#	Content
#1	"Carcinoma, Hepatocellular"[Mesh] OR "Hepatocellular carcinoma" [Title/Abstract] OR "HCC" [Title/Abstract] OR "liver cancer" [Title/Abstract] OR "liver neoplasm" [Title/Abstract]
#2	"randomized controlled trial"[Publication Type] OR "randomised controlled trial"[Title/Abstract] OR "RCT"[Title/Abstract] OR "random allocation"[MeSH]
#3	#1 AND #2

Embase

#	Content
#1	'hepatocellular carcinoma'/exp OR 'hepatocellular carcinoma' OR 'hepatocellular carcinoma':ti,ab OR 'hcc':ti,ab OR 'liver cancer':ti,ab OR 'liver neoplasm':ti,ab
#2	'randomized controlled trial'/exp OR 'randomized controlled trial' OR 'randomised controlled trial':ti,ab OR 'rct':ti,ab OR 'random allocation'/exp OR 'random allocation':ti,ab
#3	#1 AND #2

Cochrane

#	Content	No. of studies
#1	MeSH descriptor: [Carcinoma, Hepatocellular] explode all trees	2884
#2	(hepatocellular carcinoma):ti,ab,kw	6959
#3	(HCC):ti,ab,kw	4916
#4	(liver cancer):ti,ab,kw	14844
#5	(liver neoplasm):ti,ab,kw	2818
#6	#1 OR #2 OR #3 OR #4 OR #5	19782
#7	(randomized controlled trials):ti,ab,kw	101413
#8	(randomized):ti,ab,kw	1278210
#9	(randomised):ti,ab,kw	1278210
#10	(RCT):ti,ab,kw	48113
#11	(random allocation):ti,ab,kw	32461
#12	#7 OR #8 OR #9 OR #10 OR #11	1295450
#13	#6 AND #12	4011

Appendix 2: Supplementary Methods

Component network meta analysis Component network meta analysis (component NMA) was used to decompose multi-component locoregional treatments into their constituent components and to estimate the contribution of each component within the treatment network. In contrast to a standard network meta analysis (standard NMA), in which each single intervention or combination regimen is treated as a distinct node, component NMA is designed for situations where some interventions share common components and the aim is to estimate the effect attributable to those components rather than only the effect of the observed treatment regimens themselves. Under the additive assumption, the effect of a treatment combination is assumed to equal the sum of the effects of its individual components on the linear predictor scale; accordingly, common components cancel out in relative comparisons. In matrix form, the component NMA can be written as

$$\hat{\boldsymbol{\theta}} = \boldsymbol{X}\boldsymbol{\beta} + \boldsymbol{\varepsilon}, \quad \boldsymbol{\varepsilon} \sim \mathcal{N}(0, \boldsymbol{\Sigma}),$$

where $\hat{\boldsymbol{\theta}}$ denotes the vector of observed study-specific relative treatment effects, $\boldsymbol{X} = \boldsymbol{BC}$ is the design matrix combining the treatment-comparison structure and the component structure, $\boldsymbol{\beta}$ is the vector of component effects, and $\boldsymbol{\Sigma}$ is the variance-covariance matrix. Component effects are estimated using weighted least squares regression in `R` package `netmeta`. For ratio measures such as odds ratios, additivity is assumed on the log scale, so that the log-effect of a combination equals the sum of the log-effects of its components; equivalently, the effect of a combination on the ratio scale is the product of the component-specific effects.

To aid interpretation, we compared the estimates from standard NMA and component NMA for the same treatment combinations. In practical terms, the standard NMA may be viewed as the observed (empirical) effect of a combination as it appears in the available evidence network, whereas the component NMA may be viewed as the effect expected under pure additivity of components. Thus, if the estimate from standard NMA indicates a more favorable effect than that predicted by component NMA, this suggests that the combined treatment may perform better than expected from the sum of its parts, which is consistent with a synergistic effect. Conversely, if the estimate from standard NMA is less favorable than the corresponding component NMA estimate, this suggests that the observed combination effect is weaker than expected under additivity, which is consistent with a possible antagonistic effect.

Reference:

1. Kukendrarajah K, Ahmad M, Carrington M, Ioannou A, Taylor J, Razvi Y, Papageorgiou N, Mead GE, Nevis IF, D'Ascenzo F, Wilton SB, Lambiase PD, Morillo CA, Kwong JS, Providencia R. External electrical and pharmacological cardioversion for atrial fibrillation, atrial flutter or atrial tachycardias: a network meta-analysis. Cochrane Database Syst Rev. 2024 Jun 3;**6**(6):CD013255. doi: 10.1002/14651858.CD013255.pub2. PMID: 38828867; PMCID: PMC11145740.

2. Veroniki AA, Seitidis G, Nikolakopoulos S, Ballester M, Beltran J, Heijmans M, Mavridis D. Modeling Multicomponent Interventions in Network Meta-Analysis. Methods Mol Biol. 2022;**2345**:245-261. doi: 10.1007/978-1-0716-1566-9_15. PMID: 34550595.

Appendix 3: RoB 2.0 assessment of the RCTs

Abdelaziz2014

Title: Efficacy and survival analysis of percutaneous radiofrequency versus microwave ablation for hepatocellular carcinoma: an egyptian multidisciplinary clinic experience

Authors: Ashraf Abdelaziz, Tamer Elbaz, Hend Ibrahim Shousha, *et al.* **Year:** 2014

Interventions: Radiofrequency ablation (RFA) vs. Microwave ablation (MWA)

Registration number: NA

**1**	**Bias arising from the randomisation process**	
1.1	Was the allocation sequence random?<br>***Note:*** *The authors explicitly state that simple randomization was performed using the "flipping coin" method, which is a random process.*	Y
1.2	Was the allocation sequence concealed until participants were enrolled and assigned to interventions?<br>***Note:*** *The publication provides no information regarding whether or how allocation sequence concealment was implemented prior to assignment.*	NI
1.3	Did baseline differences between intervention groups suggest a problem with the randomisation process?<br>***Note:*** *A statistically significant baseline difference in age was observed between the groups (RFA group older, p=0.01). While the authors performed a univariate analysis and concluded this difference did not impact outcomes, its presence may indicate a chance imbalance or a potential issue with the randomisation implementation, albeit a minor one given the comparability of other baseline characteristics.*	PN
**Risk-of-bias judgment**		Some concerns
**2**	**Bias due to deviations from intended interventions**	
2.1	Were participants aware of their assigned intervention during the trial?<br>***Note:*** *The nature of the percutaneous ablation procedures means patients would be aware of the intervention being performed, though the technical differences between RFA and MWA might not be known to them.*	Y
2.2	Were carers and people delivering the interventions aware of participants' assigned intervention during the trial?<br>***Note:*** *The clinicians performing the procedures would necessarily be aware of the assigned intervention (RFA or MWA) as they are required to operate the different proprietary devices.*	Y
2.3	If Y/PY/NI to 2.1 or 2.2: Were there deviations from the intended intervention that arose because of the trial context?<br>***Note:*** *Considering the small amount of patients who changed the treatment, we suppose no deviation exists from the intended intervention.*	PN
2.4	If Y/PY/NI to 2.3: Were these deviations likely to have affected the outcome?	NA
2.5	If Y/PY to 2.4: Were these deviations from intended intervention balanced between groups?	NA
2.6	Was an appropriate analysis used to estimate the effect of assignment to intervention?<br>***Note:*** *The analysis appears to be based on the intention-to-treat principle, as outcomes (e.g., complications, recurrence) are reported per number of tumours/patients randomised, not per protocol.*	Y

Abdelaziz2014 *(continued)*

2.7	If N/PN/NI to 2.6: Was there potential for a substantial impact (on the result) of the failure to analyse participants in the group to which they were randomised?	NA
**Risk-of-bias judgment**		Low
**3**	**Bias due to missing outcome data**	
3.1	Were data for this outcome available for all, or nearly all, participants randomised?<br>***Note:*** *A significant proportion of participants (58/111, 52.2%) dropped out during follow-up, meaning outcome data were not available for all randomised participants.*	N
3.2	If N/PN/NI to 3.1: Is there evidence that the result was not biased by missing outcome data?<br>***Note:*** *The authors attribute the dropouts to the absence of a national registry and difficulty contacting patients, but they provide no comparative analysis of the baseline characteristics of completers versus dropouts to rule out systematic differences. Therefore, the potential for bias cannot be dismissed.*	PN
3.3	If N/PN to 3.2: Could missingness in the outcome depend on its true value?<br>***Note:*** *It is plausible that patients with poorer outcomes (e.g., disease progression, death) or those who felt well and did not seek further follow-up were more likely to be among the dropouts.*	Y
3.4	If Y/PY/NI to 3.3: Is it likely that missingness in the outcome depended on its true value?<br>***Note:*** *Given the high dropout rate and the lack of evidence to the contrary, it is likely that the missingness was not random and was related to the outcome, potentially leading to an overestimation of survival rates.*	Y
**Risk-of-bias judgment**		High
**4**	**Bias in measurement of the outcome**	
4.1	Was the method of measuring the outcome inappropriate?<br>***Note:*** *The primary outcome of complete ablation was assessed via contrast-enhanced CT imaging, which is a standard and appropriate objective radiological method for this endpoint.*	N
4.2	Could measurement or ascertainment of the outcome have differed between intervention groups?<br>***Note:*** *The same objective method (CT imaging) with predefined criteria (lack of arterial enhancement) was used uniformly across both intervention groups.*	N
4.3	If N/PN/NI to 4.1 and 4.2: Were outcome assessors aware of the intervention received by study participants?<br>***Note:*** *The radiologists interpreting the CT scans were likely aware of the treatment assignment, as this was not a blinded study and the procedures are distinct.*	Y
4.4	If Y/PY/NI to 4.3: Could assessment of the outcome have been influenced by knowledge of intervention received?<br>***Note:*** *Although the assessment is based on objective imaging, knowledge of the treatment group could theoretically introduce conscious or unconscious bias in the interpretation of ambiguous cases.*	PY
4.5	If Y/PY/NI to 4.4: Is it likely that assessment of the outcome was influenced by knowledge of intervention received?<br>***Note:*** *The outcome (contrast enhancement) is a fundamentally objective radiological finding. Therefore, while the potential for bias exists in theory, it is unlikely to have substantially influenced the results in practice.*	PN
**Risk-of-bias judgment**		Low
**5**	**Bias in selection of the reported result**	

**Abdelaziz2014** *(continued)*		
5.1	Were the data that produced this result analysed in accordance with a prespecified analysis plan that was finalised before unblinded outcome data were available for analysis?<br>***Note:*** *There is no mention of a pre-registered protocol or a statistical analysis plan finalized prior to data collection and analysis in the manuscript.*	N
Is the numerical result being assessed likely to have been selected, on the basis of the results, from:		
5.2	Multiple eligible outcome measurements (eg, scales, definitions, time points) within the outcome domain?<br>***Note:*** *The outcomes reported (e.g., complete ablation rate, local recurrence, overall survival) are standard and conventional endpoints for studies of this nature. There is no evidence in the manuscript to suggest that multiple alternative definitions or measurements for these outcomes were considered and that the reported ones were selectively chosen based on the results.*	PY
5.3	Multiple eligible analyses of the data?<br>***Note:*** *The manuscript presents a primary set of analyses using standard statistical methods (e.g., t-test, Chi-square, Kaplan-Meier). There is no indication that multiple alternative analyses were performed from which these specific results were selected. The analyses reported appear to be the only ones conducted.*	PY
**Risk-of-bias judgment**		Some concerns
**Overall bias**		High

Brunello2008

**Title:** Radiofrequency ablation versus ethanol injection for early hepatocellular carcinoma: A randomized controlled trial			
**Authors:** Franco Brunello, Andrea Veltri, Patrizia Carucci, *et al.*.		**Year:** 2008	
**Interventions:** Radiofrequency ablation (RFA) vs Percutaneous ethanol injection (PEI)			
**Registration number:** NCT00355212			
**1**	**Bias arising from the randomisation process**		
1.1	Was the allocation sequence random?<br>***Note:*** *The paper states that patients were randomized using "a computerized random number generator with random block sizes" and that the sequence was produced by the epidemiology unit. This is adequate evidence of a random sequence generation method.*		Y
1.2	Was the allocation sequence concealed until participants were enrolled and assigned to interventions?<br>***Note:*** *The study reports that operators received allocations in "sealed in closed, sequentially numbered envelopes" and that the sequence could not be predicted by operators. This supports allocation concealment.*		Y
1.3	Did baseline differences between intervention groups suggest a problem with the randomisation process?<br>***Note:*** *Most baseline characteristics were reported as not showing relevant differences between groups, although the RFA arm had somewhat fewer males and fewer tumors $\leqslant$ 20 mm. These imbalances do not clearly indicate a failure of randomization, especially in a moderate-sized trial. The authors themselves describe no major baseline imbalance.*		PN
**Risk-of-bias judgment**			Low
**2**	**Bias due to deviations from intended interventions**		
2.1	Were participants aware of their assigned intervention during the trial?<br>***Note:*** *This was a procedural trial comparing RFA with PEI, and the interventions are practically different. The paper also notes that blinded assessment of CT was considered infeasible because the two techniques produced different radiologic signs. That makes it very likely that participants knew which treatment they received.*		PY
2.2	Were carers and people delivering the interventions aware of participants' assigned intervention during the trial?<br>***Note:*** *The procedures were physically different, were delivered by experienced operators, and even involved different devices and technique-specific decisions. Blinding of treating clinicians was not possible here.*		Y
2.3	If Y/PY/NI to 2.1 or 2.2: Were there deviations from the intended intervention that arose because of the trial context?<br>***Note:*** *There is no clear evidence that knowledge of assignment caused departures from the assigned strategy beyond what was built into the protocol. Re-treatment by the same allocated method was planned, and additional management after treatment failure was also prespecified. The small number of protocol violations appears limited and not obviously driven by trial-context bias.*		PN
2.4	If Y/PY/NI to 2.3: Were these deviations likely to have affected the outcome?		NA
2.5	If Y/PY to 2.4: Were these deviations from intended intervention balanced between groups?		NA
2.6	Was an appropriate analysis used to estimate the effect of assignment to intervention?<br>***Note:*** *The paper explicitly states that an "intention-to-treat" analysis was carried out for the primary end-point. For an ITT estimand, that is the correct general approach.*		PY

Brunello2008 *(continued)*

2.7	If N/PN/NI to 2.6: Was there potential for a substantial impact (on the result) of the failure to analyse participants in the group to which they were randomised?	NA
**Risk-of-bias judgment**		Low
**3**	**Bias due to missing outcome data**	
3.1	Were data for this outcome available for all, or nearly all, participants randomised?<br>***Note:*** *Patients lost to follow-up accounted for less than 20%.*	PY
3.2	If N/PN/NI to 3.1: Is there evidence that the result was not biased by missing outcome data?	NA
3.3	If N/PN to 3.2: Could missingness in the outcome depend on its true value?	NA
3.4	If Y/PY/NI to 3.3: Is it likely that missingness in the outcome depended on its true value?	NA
**Risk-of-bias judgment**		Low
**4**	**Bias in measurement of the outcome**	
4.1	Was the method of measuring the outcome inappropriate?<br>***Note:*** *The primary outcome, CR at 1 year, was defined radiologically using CT/MR detection of a non-enhanced area of necrosis/scar at baseline lesion sites. This is an appropriate method for this kind of interventional oncology study.*	N
4.2	Could measurement or ascertainment of the outcome have differed between intervention groups?<br>***Note:*** *The same imaging-based framework was used for both groups, and follow-up imaging was part of the study protocol. While the radiologic appearance of treated lesions differed by technique, the criterion for CR was applied to both groups.*	PN
4.3	If N/PN/NI to 4.1 and 4.2: Were outcome assessors aware of the intervention received by study participants?<br>***Note:*** *The paper explicitly says that a blinded CT evaluation was considered not feasible because the two techniques produced different radiological signs. Therefore, assessors were effectively aware of intervention assignment.*	Y
4.4	If Y/PY/NI to 4.3: Could assessment of the outcome have been influenced by knowledge of intervention received?<br>***Note:*** *All the outcomes (survival or complications) included in this systematic review were relatively objective, so we deem the assessment couldn't be influenced by knowledge of intervention received.*	PN
4.5	If Y/PY/NI to 4.4: Is it likely that assessment of the outcome was influenced by knowledge of intervention received?	NA
**Risk-of-bias judgment**		Low
**5**	**Bias in selection of the reported result**	
5.1	Were the data that produced this result analysed in accordance with a prespecified analysis plan that was finalised before unblinded outcome data were available for analysis?<br>***Note:*** *This study was registered by the Clinical Trials.gov of the US National Institutes of Health (NCT00355212), where a prespecified protocol was provided.*	Y
Is the numerical result being assessed likely to have been selected, on the basis of the results, from:		
5.2	Multiple eligible outcome measurements (eg, scales, definitions, time points) within the outcome domain?<br>***Note:*** *For the primary endpoint, the paper is fairly specific: complete response at 1 year after treatment. Although there was also an early CR endpoint, that was listed as a secondary endpoint, not an alternative primary measurement.*	N

**Brunello2008** *(continued)*		
5.3	Multiple eligible analyses of the data?<br>***Note:*** *The paper reports both ITT and per-protocol analyses for response outcomes. Although both analyses were present in this study, the ITT result is correctly emphasized for the primary endpoint, and per-protocol just provides another analysis for this study.*	N
**Risk-of-bias judgment**		Low
**Overall bias**		Low

Bush2023

Title: Proton beam radiotherapy versus transarterial chemoembolization for hepatocellular carcinoma: Results of a randomized clinical trial
Authors: David A. Bush, Michael Volk, Jason C. Smith, *et al.* **Year:** 2023
Interventions: Transcatheter arterial chemoembolization (TACE) vs. Proton beam radiotherapy (RT)
Registration number: NCT00857805

1 Bias arising from the randomisation process

1.1 Was the allocation sequence random? — Y

Note: *The authors explicitly state that a "random number method" was used for sequence generation, confirming randomness.*

1.2 Was the allocation sequence concealed until participants were enrolled and assigned to interventions? — PY

Note: *The manuscript provides no description of allocation concealment mechanisms (e.g., sealed envelopes, central system). However, the sequence was generated by a study statistician, and the baseline characteristics are well-balanced (Table 1). There is no evidence to suggest that the investigators enrolling participants could have foreseen or influenced the upcoming assignment. Therefore, while concealment cannot be confirmed, there is a probable low risk of bias from this domain.*

1.3 Did baseline differences between intervention groups suggest a problem with the randomisation process? — N

Note: *Table 1 demonstrates no statistically significant differences in baseline characteristics between the two intervention groups, indicating the randomisation process was likely performed correctly.*

Risk-of-bias judgment — Low

2 Bias due to deviations from intended interventions

2.1 Were participants aware of their assigned intervention during the trial? — Y

Note: *The fundamentally different nature of the percutaneous procedures (PBT vs. TACE) made blinding of participants impossible.*

2.2 Were carers and people delivering the interventions aware of participants' assigned intervention during the trial? — Y

Note: *The clinicians administering the interventions (radiation oncologists for PBT, interventional radiologists for TACE) must have been aware of the treatment assignment to perform their respective procedures.*

2.3 If Y/PY/NI to 2.1 or 2.2: Were there deviations from the intended intervention that arose because of the trial context? — PY

Note: *A protocol deviation occurred: the TACE procedure was changed mid-study from a conventional regimen to using drug-eluting beads (DEB-TACE) due to a national shortage of ethiodol.*

2.4 If Y/PY/NI to 2.3: Were these deviations likely to have affected the outcome? — PN

Note: *While a protocol deviation occurred, the switch was from one standard form of TACE to another (DEB-TACE). DEB-TACE is an established and comparable standard-of-care treatment for HCC, not an experimental or known-to-be-inferior alternative. Therefore, it is probably not likely that this change introduced a significant bias affecting the comparative outcomes between the TACE and PBT arms.*

2.5 If Y/PY to 2.4: Were these deviations from intended intervention balanced between groups? — NA

Bush2023 *(continued)*

2.6	Was an appropriate analysis used to estimate the effect of assignment to intervention?<br>***Note:*** *An intention-to-treat (ITT) analysis was appropriately used, as all randomized patients who commenced treatment were included in the analysis of the primary outcome.*	Y
2.7	If N/PN/NI to 2.6: Was there potential for a substantial impact (on the result) of the failure to analyse participants in the group to which they were randomised?	NA
**Risk-of-bias judgment**		Some concerns
**3**	**Bias due to missing outcome data**	
3.1	Were data for this outcome available for all, or nearly all, participants randomised?<br>***Note:*** *Figure 1 (CONSORT flow diagram) indicates that there were "lost to follow-up (n=0)" for both arms after allocation. All 74 subjects who received treatment were analysed, indicating complete outcome data for the randomized participants who commenced treatment.*	Y
3.2	If N/PN/NI to 3.1: Is there evidence that the result was not biased by missing outcome data?	NA
3.3	If N/PN to 3.2: Could missingness in the outcome depend on its true value?	NA
3.4	If Y/PY/NI to 3.3: Is it likely that missingness in the outcome depended on its true value?	NA
**Risk-of-bias judgment**		Low
**4**	**Bias in measurement of the outcome**	
4.1	Was the method of measuring the outcome inappropriate?<br>***Note:*** *Overall survival (primary outcome) is an objective, robust endpoint. Secondary outcomes like progression-free survival and local control were assessed using standard radiographic criteria (CT/MRI) based on the resolution of arterial enhancement, which is appropriate and standardized.*	N
4.2	Could measurement or ascertainment of the outcome have differed between intervention groups?<br>***Note:*** *The same objective methods for assessing outcomes (imaging protocols and clinical evaluation schedules) were applied uniformly to both groups at the same intervals (every 3 months).*	N
4.3	If N/PN/NI to 4.1 and 4.2: Were outcome assessors aware of the intervention received by study participants?<br>***Note:*** *The study was not blinded. Radiologists and clinicians assessing imaging and clinical outcomes would likely have been aware of the treatment assignment.*	Y
4.4	If Y/PY/NI to 4.3: Could assessment of the outcome have been influenced by knowledge of intervention received?<br>***Note:*** *While assessors were not blinded, the key outcomes are highly objective. Overall survival is not subject to assessment bias. Local control and progression were based on predefined, objective radiographic criteria (presence/absence of arterial enhancement), minimizing the room for subjective interpretation. Toxicity was graded using the standardized Common Terminology Criteria for Adverse Events (CTCAE). Given the interventional nature of the treatments where placebo effects are minimal, it is probably not likely that knowledge of the assignment influenced the assessment of these objective endpoints.*	PN
4.5	If Y/PY/NI to 4.4: Is it likely that assessment of the outcome was influenced by knowledge of intervention received?	NA
**Risk-of-bias judgment**		Low
**5**	**Bias in selection of the reported result**	
5.1	Were the data that produced this result analysed in accordance with a prespecified analysis plan that was finalised before unblinded outcome data were available for analysis?	Y

Bush2023 *(continued)*

	Note: *The statistical analysis of this study is in accordance to the previously registered protocol (NCT00857805).*	
	Is the numerical result being assessed likely to have been selected, on the basis of the results, from:	
5.2	Multiple eligible outcome measurements (eg, scales, definitions, time points) within the outcome domain?	N
	Note: *The outcomes reported (Overall Survival, Progression-Free Survival, Local Control, Toxicity, Cost) are standard, pre-specified endpoints for this type of trial. There is no evidence to suggest multiple alternative definitions or measurements for these outcomes were available and selected from.*	
5.3	Multiple eligible analyses of the data?	N
	Note: *The manuscript presents primary analyses using standard methods (Kaplan-Meier, log-rank test, Cox regression). There is no indication that multiple alternative analyses were performed for the primary or key secondary outcomes from which these results were selected. The multivariable analyses adjust for expected prognostic factors.*	
**Risk-of-bias judgment**		Low
**Overall bias**		Some concerns

Chen2006

**Title:** A Prospective Randomized Trial Comparing Percutaneous Local Ablative Therapy and Partial Hepatectomy for Small Hepatocellular Carcinoma		
**Authors:** Min-Shan Chen, Jin-Qing Li, Yun Zheng, *et al.*.		**Year:** 2006
**Interventions:** Radiofrequency ablation (RFA) vs Surgical resection (SR)		
**Registration number:** NA		
**1**	**Bias arising from the randomisation process**	
1.1	Was the allocation sequence random?<br>***Note:*** *The authors state that "randomization was done by using random numbers generated from a computer," confirming a random sequence.*	Y
1.2	Was the allocation sequence concealed until participants were enrolled and assigned to interventions?<br>***Note:*** *The manuscript states randomization was done "in a central registry for this study." This suggests a centralized process, which probably ensured allocation concealment, but the specific mechanism (e.g., sealed envelopes, phone line) is not described in detail.*	PY
1.3	Did baseline differences between intervention groups suggest a problem with the randomisation process?<br>***Note:*** *Table 1 shows a statistically significant difference in serum ALT levels between the two groups (p=0.046). All other baseline characteristics were comparable. This single imbalance may be a chance finding but could indicate a minor issue with the randomisation process.*	PN
**Risk-of-bias judgment**		Low
**2**	**Bias due to deviations from intended interventions**	
2.1	Were participants aware of their assigned intervention during the trial?<br>***Note:*** *The nature of the interventions (percutaneous ablation vs. major surgery) is fundamentally different and impossible to blind from the patients.*	Y
2.2	Were carers and people delivering the interventions aware of participants' assigned intervention during the trial?<br>***Note:*** *The surgeons and interventional radiologists performing the procedures must have been aware of the treatment assignment to administer the correct therapy.*	Y
2.3	If Y/PY/NI to 2.1 or 2.2: Were there deviations from the intended intervention that arose because of the trial context?<br>***Note:*** *A significant protocol deviation occurred: 19 patients (21%) randomized to the PLAT group withdrew consent and instead received surgical resection. This deviation arose specifically because of the trial context (patient preference after randomization).*	PY
2.4	If Y/PY/NI to 2.3: Were these deviations likely to have affected the outcome?<br>***Note:*** *The authors performed both Intention-to-Treat (ITT) and per-protocol analyses, mitigating this risk.*	PN
2.5	If Y/PY to 2.4: Were these deviations from intended intervention balanced between groups?	NA
2.6	Was an appropriate analysis used to estimate the effect of assignment to intervention?<br>***Note:*** *The authors performed both an Intention-to-Treat (ITT) analysis (including the 19 crossover patients in the PLAT group) and a per-protocol analysis. The primary conclusions are based on the ITT analysis, which is the appropriate method to estimate the effect of assignment.*	Y
2.7	If N/PN/NI to 2.6: Was there potential for a substantial impact (on the result) of the failure to analyse participants in the group to which they were randomised?	NA

Chen2006 *(continued)*

**Risk-of-bias judgment**		Some concerns
**3**	**Bias due to missing outcome data**	
3.1	Were data for this outcome available for all, or nearly all, participants randomised?<br>***Note:*** *The flow chart (Figure 1) and text indicate that all 180 randomized patients were included in the analysis ("all analyses were done on the intention-to-treat basis"). There is no mention of patients being lost to follow-up.*	Y
3.2	If N/PN/NI to 3.1: Is there evidence that the result was not biased by missing outcome data?	NA
3.3	If N/PN to 3.2: Could missingness in the outcome depend on its true value?	NA
3.4	If Y/PY/NI to 3.3: Is it likely that missingness in the outcome depended on its true value?	NA
**Risk-of-bias judgment**		Low
**4**	**Bias in measurement of the outcome**	
4.1	Was the method of measuring the outcome inappropriate?<br>***Note:*** *The primary outcomes (overall survival and disease-free survival) were objective. Survival is a robust endpoint, and recurrence was assessed using standard radiographic criteria (CT/MRI enhancement).*	N
4.2	Could measurement or ascertainment of the outcome have differed between intervention groups?<br>***Note:*** *The same methods for assessing outcomes (imaging protocols and schedules) were applied uniformly to both groups.*	N
4.3	If N/PN/NI to 4.1 and 4.2: Were outcome assessors aware of the intervention received by study participants?<br>***Note:*** *The study was not blinded. Radiologists assessing CT/MRI scans would likely have been aware of the treatment assignment, as the surgical changes or ablation zone characteristics might be apparent.*	Y
4.4	If Y/PY/NI to 4.3: Could assessment of the outcome have been influenced by knowledge of intervention received?<br>***Note:*** *Although the assessors were not blinded, the key outcomes are based on objective evidence (death, radiographic recurrence). The interventional/surgical nature of the treatments minimizes placebo effects. Therefore, it is probably not likely that knowledge of the assignment significantly influenced the assessment of these objective endpoints.*	PN
4.5	If Y/PY/NI to 4.4: Is it likely that assessment of the outcome was influenced by knowledge of intervention received?	NA
**Risk-of-bias judgment**		Low
**5**	**Bias in selection of the reported result**	
5.1	Were the data that produced this result analysed in accordance with a prespecified analysis plan that was finalised before unblinded outcome data were available for analysis?<br>***Note:*** *The trial was initiated in 1999, before common practice of public trial registration. There is no mention of a protocol or statistical analysis plan being published or registered prior to the study start. The sample size calculation is described, but a full pre-specified analysis plan is not evident. However, due to the precise description of Methods and honest presentation of drop-out, we tended to believe that there is precise prespecified analysis protocol, and the researches strictly followed it.*	PY

Is the numerical result being assessed likely to have been selected, on the basis of the results, from:

**Chen2006** *(continued)*		
5.2	Multiple eligible outcome measurements (eg, scales, definitions, time points) within the outcome domain?<br>***Note:*** *The outcomes reported (overall survival, disease-free survival) are standard and conventional endpoints for this type of trial. There is no evidence to suggest that multiple alternative definitions or measurements for these outcomes were available and that the reported ones were selectively chosen.*	N
5.3	Multiple eligible analyses of the data?<br>***Note:*** *The manuscript presents both ITT and per-protocol analyses, which is a standard and expected approach for a trial with cross-over. The results are consistent across both analyses. There is no indication that multiple alternative analyses were performed and that these specific results were selected from among them.*	N
**Risk-of-bias judgment**		Low
**Overall bias**		Some concerns

Chen2014

**Title:** Increased survival in hepatocellular carcinoma with iodine-125 implantation plus radiofrequency ablation: A prospective randomized controlled trial	
**Authors:** Kaiyun Chen, Guihua Chen, Hanning Wang, *et al.*.	**Year:** 2014
**Interventions:** Radiofrequency ablation (RFA) + Percutaneous iodine-125 (^{125}I) implantation vs RFA	
**Registration number:** NCT01717729	

**1**	**Bias arising from the randomisation process**	
1.1	Was the allocation sequence random?<br>***Note:*** *The authors state that allocation was done "with a computer-generated list using a randomly permuted block design," confirming a random sequence.*	Y
1.2	Was the allocation sequence concealed until participants were enrolled and assigned to interventions?<br>***Note:*** *The manuscript states that "each patient's treatment group assignment was concealed from the doctor prior to the treatment." This was managed through a central telephone allocation system at the data centre. This describes a robust process that probably ensured concealment.*	PY
1.3	Did baseline differences between intervention groups suggest a problem with the randomisation process?<br>***Note:*** *Table 1 demonstrates no statistically significant differences in baseline characteristics between the two intervention groups (RFA-*^{125}I *vs. RFA-only), indicating the randomisation process was performed correctly.*	N
**Risk-of-bias judgment**		Low
**2**	**Bias due to deviations from intended interventions**	
2.1	Were participants aware of their assigned intervention during the trial?<br>***Note:*** *The nature of the interventions is different (one involves seed implantation, the other does not). Furthermore, the manuscript states that bandages were applied to all patients to maintain blinding, implying that without this measure, patients would be aware, confirming that they were not inherently blinded to the treatment.*	Y
2.2	Were carers and people delivering the interventions aware of participants' assigned intervention during the trial?<br>***Note:*** *The surgeons and radiation oncologists performing the procedures (RFA and seed implantation) must have been aware of the treatment assignment to administer the correct therapy.*	Y
2.3	If Y/PY/NI to 2.1 or 2.2: Were there deviations from the intended intervention that arose because of the trial context?<br>***Note:*** *The manuscript does not report any protocol deviations that occurred after assignment due to the trial context. The procedures appear to have been delivered as intended.*	N
2.4	If Y/PY/NI to 2.3: Were these deviations likely to have affected the outcome?	NA
2.5	If Y/PY to 2.4: Were these deviations from intended intervention balanced between groups?	NA
2.6	Was an appropriate analysis used to estimate the effect of assignment to intervention?<br>***Note:*** *The analysis was performed on an intention-to-treat (ITT) basis, as confirmed by the flow chart (Figure 2) and the statement "Intention-to-Treat Analysis".*	Y
2.7	If N/PN/NI to 2.6: Was there potential for a substantial impact (on the result) of the failure to analyse participants in the group to which they were randomised?	NA
**Risk-of-bias judgment**		Low
**3**	**Bias due to missing outcome data**	

Chen2014 *(continued)*

3.1	Were data for this outcome available for all, or nearly all, participants randomised?<br>***Note:*** *The flow chart (Figure 2) explicitly states "lost to follow-up n = 0" for both groups, indicating complete outcome data for all randomised participants.*	Y
3.2	If N/PN/NI to 3.1: Is there evidence that the result was not biased by missing outcome data?	NA
3.3	If N/PN to 3.2: Could missingness in the outcome depend on its true value?	NA
3.4	If Y/PY/NI to 3.3: Is it likely that missingness in the outcome depended on its true value?	NA
**Risk-of-bias judgment**		Low
**4**	**Bias in measurement of the outcome**	
4.1	Was the method of measuring the outcome inappropriate?<br>***Note:*** *The primary outcome (recurrence) and key secondary outcome (overall survival) were assessed using objective and standard methods: CT/MRI imaging based on predefined radiographic criteria (enhancement) for recurrence, and death for survival.*	N
4.2	Could measurement or ascertainment of the outcome have differed between intervention groups?<br>***Note:*** *The same objective methods for assessing outcomes (imaging protocols and schedules) were applied uniformly to both groups.*	N
4.3	If N/PN/NI to 4.1 and 4.2: Were outcome assessors aware of the intervention received by study participants?<br>***Note:*** *The study was not blinded. Radiologists assessing CT/MRI scans for recurrence would likely have been aware of the treatment assignment, as the presence or absence of iodine seeds would be visible on the scans.*	Y
4.4	If Y/PY/NI to 4.3: Could assessment of the outcome have been influenced by knowledge of intervention received?<br>***Note:*** *Although the assessors were not blinded, the outcomes are based on objective radiographic evidence (presence of contrast enhancement). The interventional nature of the treatments minimizes placebo effects. Therefore, it is probably not likely that knowledge of the assignment significantly influenced the assessment of these objective endpoints.*	PN
4.5	If Y/PY/NI to 4.4: Is it likely that assessment of the outcome was influenced by knowledge of intervention received?	NA
**Risk-of-bias judgment**		Low
**5**	**Bias in selection of the reported result**	
5.1	Were the data that produced this result analysed in accordance with a prespecified analysis plan that was finalised before unblinded outcome data were available for analysis?<br>***Note:*** *The trial was prospectively registered on ClinicalTrials.gov (NCT01717729). The statistical analyses for the primary (recurrence) and secondary (overall survival) outcomes reported in the manuscript align with the outcomes pre-specified in the registry entry.*	Y
Is the numerical result being assessed likely to have been selected, on the basis of the results, from:		
5.2	Multiple eligible outcome measurements (eg, scales, definitions, time points) within the outcome domain?<br>***Note:*** *The outcomes reported are the standard, pre-specified endpoints (recurrence, survival). There is no evidence to suggest that multiple alternative definitions or measurements for these outcomes were available and that the reported ones were selectively chosen.*	N
5.3	Multiple eligible analyses of the data?	N

**Chen2014** *(continued)*	
Note: *The manuscript presents primary analyses using standard methods (Kaplan-Meier, log-rank test, Cox regression) that would be expected for the pre-specified outcomes. There is no indication that multiple alternative analyses were performed and selected from.*	
**Risk-of-bias judgment**	Low
**Overall bias**	Low

Chong2020

**Title:** Prospective double-blinded randomized controlled trial of Microwave versus RadioFrequency Ablation for hepatocellular carcinoma (McRFA trial)		
**Authors:** Charing C.N. Chong, Kit F. Lee, Sunny Y.S. Cheung, *et al.*		**Year:** 2020
**Interventions:** Microwave (MWA) vs. Radiofrequency ablation (RFA)		
**Registration:** NCT01340105		
**1**	**Bias arising from the randomisation process**	
1.1	Was the allocation sequence random?<br>***Note:*** *The authors state that "randomization was made by a computer-generated random number list," which indicates a truly random allocation sequence.*	Y
1.2	Was the allocation sequence concealed until participants were enrolled and assigned to interventions?<br>***Note:*** *Although the method of sequence generation is described, there is no explicit statement regarding concealment of the allocation sequence. However, there is no evidence of selective enrollment or manipulation, so the risk is probably low.*	Y
1.3	Did baseline differences between intervention groups suggest a problem with the randomisation process?<br>***Note:*** *Baseline demographics and tumor characteristics were comparable between groups except for gender distribution (p = 0.041), which is likely due to chance and not indicative of systematic bias.*	N
**Risk-of-bias judgment**		Low
**2**	**Bias due to deviations from intended interventions**	
2.1	Were participants aware of their assigned intervention during the trial?<br>***Note:*** *The study is described as "double-blinded." Patients were blinded to the treatment allocation.*	N
2.2	Were carers and people delivering the interventions aware of participants' assigned intervention during the trial?<br>***Note:*** *The interventional radiologists and surgeons performing the procedures were necessarily aware of the treatment modality.*	Y
2.3	If Y/PY/NI to 2.1 or 2.2: Were there deviations from the intended intervention that arose because of the trial context?<br>***Note:*** *No evidence suggest a significant deviation because of the trial context.*	PN
2.4	If Y/PY/NI to 2.3: Were these deviations likely to have affected the outcome?	NA
2.5	If Y/PY to 2.4: Were these deviations from intended intervention balanced between groups?	NA
2.6	Was an appropriate analysis used to estimate the effect of assignment to intervention?<br>***Note:*** *An intention-to-treat analysis was performed, which is appropriate for estimating the effect of assignment to intervention.*	Y
2.7	If N/PN/NI to 2.6: Was there potential for a substantial impact (on the result) of the failure to analyse participants in the group to which they were randomised?	NA
**Risk-of-bias judgment**		Low
**3**	**Bias due to missing outcome data**	
3.1	Were data for this outcome available for all, or nearly all, participants randomised?<br>***Note:*** *All 93 randomised patients were included in the analysis except for three excluded due to small tumor size, which was protocol-driven and unlikely to introduce bias.*	Y

Chong2020 *(continued)*

3.2	If N/PN/NI to 3.1: Is there evidence that the result was not biased by missing outcome data?	NA
3.3	If N/PN to 3.2: Could missingness in the outcome depend on its true value?	NA
3.4	If Y/PY/NI to 3.3: Is it likely that missingness in the outcome depended on its true value?	NA
**Risk-of-bias judgment**		Low
**4**	**Bias in measurement of the outcome**	
4.1	Was the method of measuring the outcome inappropriate?<br>***Note:*** *Contrast-enhanced CT scans at predefined intervals and AFP levels are standard and appropriate methods for assessing ablation completeness and recurrence.*	N
4.2	Could measurement or ascertainment of the outcome have differed between intervention groups?<br>***Note:*** *The same imaging protocol and timing were used for both groups.*	N
4.3	If N/PN/NI to 4.1 and 4.2: Were outcome assessors aware of the intervention received by study participants?<br>***Note:*** *The radiologists interpreting the CT scans were blinded to the treatment allocation.*	N
4.4	If Y/PY/NI to 4.3: Could assessment of the outcome have been influenced by knowledge of intervention received?	NA
4.5	If Y/PY/NI to 4.4: Is it likely that assessment of the outcome was influenced by knowledge of intervention received?	NA
**Risk-of-bias judgment**		Low
**5**	**Bias in selection of the reported result**	
5.1	Were the data that produced this result analysed in accordance with a prespecified analysis plan that was finalised before unblinded outcome data were available for analysis?<br>***Note:*** *The study was registered at ClinicalTrials.gov (NCT01340105), and the statistical methods described align with standard pre-specified outcomes for such trials.*	Y
Is the numerical result being assessed likely to have been selected, on the basis of the results, from:		
5.2	Multiple eligible outcome measurements (eg, scales, definitions, time points) within the outcome domain?<br>***Note:*** *The primary and secondary outcomes are clearly defined and consistent with the trial registration and methodology section.*	N
5.3	Multiple eligible analyses of the data?<br>***Note:*** *The analysis plan appears fixed, with no indication of multiple analytical approaches being conducted or selectively reported.*	N
**Risk-of-bias judgment**		Low
**Overall bias**		Low

Costanzo2015

Title: Radiofrequency ablation versus laser ablation for the treatment of small hepatocellular carcinoma in cirrhosis: A randomized trial

Authors: Giovan Giuseppe Di Costanzo, Raffaella Tortora, Giuseppe D'Adamo, *et al.* **Year:** 2015

Interventions: Radiofrequency ablation (RFA) vs. Laser ablation (LA)

Registration: NCT01096914

**1**	**Bias arising from the randomisation process**	
1.1	Was the allocation sequence random?<br>***Note:*** *The authors state that randomization was based on "computer-generated random numbers", indicating a random allocation sequence.*	Y
1.2	Was the allocation sequence concealed until participants were enrolled and assigned to interventions?<br>***Note:*** *The computer-generated randomization method is used.*	Y
1.3	Did baseline differences between intervention groups suggest a problem with the randomisation process?<br>***Note:*** *Baseline characteristics were generally comparable. Although there were more large and high-risk nodules in the LA group, the differences were not statistically significant, and the authors attributed this to random variation.*	N
**Risk-of-bias judgment**		Low
**2**	**Bias due to deviations from intended interventions**	
2.1	Were participants aware of their assigned intervention during the trial?<br>***Note:*** *The study does not explicitly state that patients were blinded, but given the nature of the percutaneous procedures under sedation, it is unlikely that patients were aware of the specific ablation modality used.*	N
2.2	Were carers and people delivering the interventions aware of participants' assigned intervention during the trial?<br>***Note:*** *The operators performing the procedures were necessarily aware of the treatment assignment.*	Y
2.3	If Y/PY/NI to 2.1 or 2.2: Were there deviations from the intended intervention that arose because of the trial context?<br>***Note:*** *There is no information suggesting that deviations occurred due to the trial context. The procedures were performed per standard protocols.*	PN
2.4	If Y/PY/NI to 2.3: Were these deviations likely to have affected the outcome?	NA
2.5	If Y/PY to 2.4: Were these deviations from intended intervention balanced between groups?	NA
2.6	Was an appropriate analysis used to estimate the effect of assignment to intervention?<br>***Note:*** *An intention-to-treat analysis was performed, which is appropriate for estimating the effect of assignment to intervention.*	Y
2.7	If N/PN/NI to 2.6: Was there potential for a substantial impact (on the result) of the failure to analyse participants in the group to which they were randomised?	NA
**Risk-of-bias judgment**		Low
**3**	**Bias due to missing outcome data**	
3.1	Were data for this outcome available for all, or nearly all, participants randomised?	Y

	***Note:** All 140 randomised patients were included in the analysis. Three patients in the LA group did not receive repeat ablation (for valid clinical reasons), but they were included in the intention-to-treat analysis.*	
3.2	If N/PN/NI to 3.1: Is there evidence that the result was not biased by missing outcome data?	NA
3.3	If N/PN to 3.2: Could missingness in the outcome depend on its true value?	NA
3.4	If Y/PY/NI to 3.3: Is it likely that missingness in the outcome depended on its true value?	NA
**Risk-of-bias judgment**		Low
**4**	**Bias in measurement of the outcome**	
4.1	Was the method of measuring the outcome inappropriate?	N
	***Note:** Contrast-enhanced CT or MRI at 4 weeks post-ablation is a standard and appropriate method for assessing complete tumor ablation (CTA).*	
4.2	Could measurement or ascertainment of the outcome have differed between intervention groups?	N
	***Note:** The same imaging modalities and timing were used for both groups.*	
4.3	If N/PN/NI to 4.1 and 4.2: Were outcome assessors aware of the intervention received by study participants?	N
	***Note:** The radiologists interpreting the imaging studies were blinded to the treatment allocation, as stated in the methods: "The radiologists who interpreted the post-procedure CT scans were blinded to the type of ablation".*	
4.4	If Y/PY/NI to 4.3: Could assessment of the outcome have been influenced by knowledge of intervention received?	NA
4.5	If Y/PY/NI to 4.4: Is it likely that assessment of the outcome was influenced by knowledge of intervention received?	NA
**Risk-of-bias judgment**		Low
**5**	**Bias in selection of the reported result**	
5.1	Were the data that produced this result analysed in accordance with a prespecified analysis plan that was finalised before unblinded outcome data were available for analysis?	Y
	***Note:** The study was registered at ClinicalTrials.gov (NCT01096914), and the statistical methods and outcomes align with the pre-specified non-inferiority design.*	
Is the numerical result being assessed likely to have been selected, on the basis of the results, from:		
5.2	Multiple eligible outcome measurements (eg, scales, definitions, time points) within the outcome domain?	N
	***Note:** The primary outcome (CTA) and secondary outcomes (TTLP, OS) are clearly defined and consistent with the trial registration.*	
5.3	Multiple eligible analyses of the data?	N
	***Note:** The analysis plan appears fixed, with no indication of multiple analytical approaches being conducted or selectively reported.*	
**Risk-of-bias judgment**		Low
**Overall bias**		Low

Fang2014

Title: Comparison of long-term effectiveness and complications of radiofrequency ablation with hepatectomy for small hepatocellular carcinoma

Authors: Yong Fang, Wei Chen, Xiao Liang, *et al.* **Year:** 2014

Interventions: Radiofrequency ablation (RFA) vs. Surgical resection (SR)

Registration: NA

**1**	**Bias arising from the randomisation process**	
1.1	Was the allocation sequence random?<br>***Note:*** *The authors state that patients were "randomized into either percutaneous RFA therapy or hepatectomy group". However, the method of randomisation (e.g., computer-generated) is not explicitly described.*	PY
1.2	Was the allocation sequence concealed until participants were enrolled and assigned to interventions?<br>***Note:*** *There is no mention of allocation concealment (e.g., sealed opaque envelopes, central system). The lack of detail suggests concealment was probably not ensured.*	PN
1.3	Did baseline differences between intervention groups suggest a problem with the randomisation process?<br>***Note:*** *Baseline characteristics were mostly similar, but a significant imbalance existed in Child–Pugh classification (p=0.021), with more Class C patients in the RFA group. This may represent a chance imbalance rather than a systematic flaw in randomisation.*	PN
**Risk-of-bias judgment**		High
**2**	**Bias due to deviations from intended interventions**	
2.1	Were participants aware of their assigned intervention during the trial?<br>***Note:*** *The nature of the interventions (RFA vs. surgery) makes blinding of participants impossible.*	Y
2.2	Were carers and people delivering the interventions aware of participants' assigned intervention during the trial?<br>***Note:*** *Clinicians performing RFA or surgery were unavoidably aware of the treatment assignment.*	Y
2.3	If Y/PY/NI to 2.1 or 2.2: Were there deviations from the intended intervention that arose because of the trial context?<br>***Note:*** *No specific deviations due to the trial context are reported. The protocol appears to have been followed.*	PN
2.4	If Y/PY/NI to 2.3: Were these deviations likely to have affected the outcome?	NA
2.5	If Y/PY to 2.4: Were these deviations from intended intervention balanced between groups?	NA
2.6	Was an appropriate analysis used to estimate the effect of assignment to intervention?<br>***Note:*** *The authors used intention-to-treat analysis, as all randomised patients were included in the efficacy and safety analyses.*	Y
2.7	If N/PN/NI to 2.6: Was there potential for a substantial impact (on the result) of the failure to analyse participants in the group to which they were randomised?	NA
**Risk-of-bias judgment**		Low
**3**	**Bias due to missing outcome data**	
3.1	Were data for this outcome available for all, or nearly all, participants randomised?<br>***Note:*** *All 120 randomised patients were included in the analysis, with no loss to follow-up mentioned.*	Y

Fang2014 *(continued)*

3.2	If N/PN/NI to 3.1: Is there evidence that the result was not biased by missing outcome data?	NA
3.3	If N/PN to 3.2: Could missingness in the outcome depend on its true value?	NA
3.4	If Y/PY/NI to 3.3: Is it likely that missingness in the outcome depended on its true value?	NA
**Risk-of-bias judgment**		Low
**4**	**Bias in measurement of the outcome**	
4.1	Was the method of measuring the outcome inappropriate?	N
	Note: *Overall survival, disease-free survival, and complication rates were measured using standard and appropriate clinical and radiological methods.*	
4.2	Could measurement or ascertainment of the outcome have differed between intervention groups?	PN
	Note: *Although the trial was open-label, the primary outcomes (survival, recurrence) are objective and based on imaging and clinical follow-up, reducing the likelihood of differential measurement.*	
4.3	If N/PN/NI to 4.1 and 4.2: Were outcome assessors aware of the intervention received by study participants?	Y
	Note: *The trial was open-label, so assessors were likely aware of the treatment assignment.*	
4.4	If Y/PY/NI to 4.3: Could assessment of the outcome have been influenced by knowledge of intervention received?	PN
	Note: *Given the objective nature of survival and recurrence endpoints, it is unlikely that assessors' knowledge significantly influenced the results.*	
4.5	If Y/PY/NI to 4.4: Is it likely that assessment of the outcome was influenced by knowledge of intervention received?	NA
**Risk-of-bias judgment**		Low
**5**	**Bias in selection of the reported result**	
5.1	Were the data that produced this result analysed in accordance with a prespecified analysis plan that was finalised before unblinded outcome data were available for analysis?	PN
	Note: *There is no mention of a pre-registered protocol (e.g., on ClinicalTrials.gov) or a pre-specified statistical analysis plan.*	
Is the numerical result being assessed likely to have been selected, on the basis of the results, from:		
5.2	Multiple eligible outcome measurements (eg, scales, definitions, time points) within the outcome domain?	PN
	Note: *The primary outcomes (OS, DFS, complications) are clearly defined and standard for such trials. There is no evidence of selective reporting from multiple measures.*	
5.3	Multiple eligible analyses of the data?	PN
	Note: *The statistical methods (Kaplan-Meier, log-rank test) are standard and pre-specified in the methods section. There is no indication of analysis shopping.*	
**Risk-of-bias judgment**		Some concerns
**Overall bias**		High

Fang2023

Title: Hepatectomy versus transcatheter arterial chemoembolization for resectable BCLC stage A/B hepatocellular carcinoma beyond Milan criteria: A randomized clinical trial

Authors: Chongkai Fang, Rui Luo, Ying Zhang, *et al.* **Year:** 2023

Interventions: Transcatheter arterial chemoembolization (TACE) + Surgical resection (SR) vs. SR

Registration: ChiCTR2200055618

**1**	**Bias arising from the randomisation process**	
1.1	Was the allocation sequence random?<br>***Note:*** *The authors state that patients were randomized using a "sealed envelope system", which implies the use of a random allocation sequence, although the specific method of sequence generation (e.g., computer-generated) is not explicitly detailed.*	Y
1.2	Was the allocation sequence concealed until participants were enrolled and assigned to interventions?<br>***Note:*** *The use of a "sealed envelope system" is a standard and acceptable method for allocation concealment, ensuring that the sequence was concealed until participants were enrolled and assigned.*	Y
1.3	Did baseline differences between intervention groups suggest a problem with the randomisation process?<br>***Note:*** *Baseline characteristics were generally comparable between groups, except for a significantly higher incidence of cirrhosis in the hepatectomy-alone (OP) group (48.8% vs. 23.2%, p=0.001). This imbalance in a potential prognostic factor suggests a possible issue with the randomization process, although it may be due to chance.*	PN
**Risk-of-bias judgment**		Low
**2**	**Bias due to deviations from intended interventions**	
2.1	Were participants aware of their assigned intervention during the trial?<br>***Note:*** *The study is described as "open-label", so participants were aware of their treatment assignment.*	Y
2.2	Were carers and people delivering the interventions aware of participants' assigned intervention during the trial?<br>***Note:*** *As an open-label trial, clinicians and surgeons were necessarily aware of the treatment assignment.*	Y
2.3	If Y/PY/NI to 2.1 or 2.2: Were there deviations from the intended intervention that arose because of the trial context?<br>***Note:*** *There is no information to suggest that deviations from the intended intervention occurred due to the trial context. The procedures appear to have been delivered as per protocol.*	N
2.4	If Y/PY/NI to 2.3: Were these deviations likely to have affected the outcome?	NA
2.5	If Y/PY to 2.4: Were these deviations from intended intervention balanced between groups?	NA
2.6	Was an appropriate analysis used to estimate the effect of assignment to intervention?<br>***Note:*** *An intention-to-treat analysis was performed, which is appropriate for estimating the effect of assignment to intervention.*	Y
2.7	If N/PN/NI to 2.6: Was there potential for a substantial impact (on the result) of the failure to analyse participants in the group to which they were randomised?	NA
**Risk-of-bias judgment**		Low
**3**	**Bias due to missing outcome data**	

Fang2023 *(continued)*

3.1	Were data for this outcome available for all, or nearly all, participants randomised?<br>***Note:*** *Of 172 randomized patients, 164 (82 in each group) were included in the final analysis. Eight patients were excluded (4 in each group) for reasons such as lack of pathology evidence or receiving other therapies. The proportion of missing data is small (4.65%) and unlikely to introduce significant bias.*	PY
3.2	If N/PN/NI to 3.1: Is there evidence that the result was not biased by missing outcome data?	NA
3.3	If N/PN to 3.2: Could missingness in the outcome depend on its true value?	NA
3.4	If Y/PY/NI to 3.3: Is it likely that missingness in the outcome depended on its true value?	NA
**Risk-of-bias judgment**		Low
**4**	**Bias in measurement of the outcome**	
4.1	Was the method of measuring the outcome inappropriate?<br>***Note:*** *The primary outcomes were overall survival (OS) and progression-free survival (PFS), which are objective and standard endpoints in oncology trials. Death and recurrence are robust outcomes unlikely to be influenced by measurement method.*	N
4.2	Could measurement or ascertainment of the outcome have differed between intervention groups?<br>***Note:*** *The criteria for determining death and recurrence were the same for both groups and were based on standard clinical and imaging follow-up.*	N
4.3	If N/PN/NI to 4.1 and 4.2: Were outcome assessors aware of the intervention received by study participants?<br>***Note:*** *Outcome assessors were likely aware of group allocation due to open-label design.*	PY
4.4	If Y/PY/NI to 4.3: Could assessment of the outcome have been influenced by knowledge of intervention received?<br>***Note:*** *Knowledge of intervention was unlikely to influence assessment of objective imaging-based outcomes.*	N
4.5	If Y/PY/NI to 4.4: Is it likely that assessment of the outcome was influenced by knowledge of intervention received?	NA
**Risk-of-bias judgment**		Low
**5**	**Bias in selection of the reported result**	
5.1	Were the data that produced this result analysed in accordance with a prespecified analysis plan that was finalised before unblinded outcome data were available for analysis?<br>***Note:*** *The study was registered in the Chinese Clinical Trial Registry (ChiCTR2200055618), and the primary and secondary outcomes (OS, PFS, AEs) align with the registration. The statistical analysis plan (Kaplan-Meier, log-rank test, Cox model) is standard for such trials.*	PY
Is the numerical result being assessed likely to have been selected, on the basis of the results, from:		
5.2	Multiple eligible outcome measurements (eg, scales, definitions, time points) within the outcome domain?<br>***Note:*** *The outcomes (OS, PFS) are clearly defined and consistent with the trial registration.*	N
5.3	Multiple eligible analyses of the data?<br>***Note:*** *The analysis plan appears fixed, with no indication of multiple analytical approaches being tried or selectively reported.*	N
**Risk-of-bias judgment**		Low
**Overall bias**		Low

Feng2012

**Title:** A randomized controlled trial of radiofrequency ablation and surgical resection in the treatment of small hepatocellular carcinoma		
**Authors:** Kai Feng, Jun Yan, Xiaowu Li, *et al.*		**Year:** 2012
**Interventions:** Radiofrequency ablation (RFA) vs. Surgical resection (SR)		
**Registration:** NA		
**1**	**Bias arising from the randomisation process**	
1.1	Was the allocation sequence random?<br>***Note:*** *Randomization was performed using a computer-generated sequence.*	Y
1.2	Was the allocation sequence concealed until participants were enrolled and assigned to interventions?<br>***Note:*** *Randomization was done using sealed envelopes, but the concealment process (e.g., envelope opacity, sequence safeguarding, who assigned patients) was not described.*	PY
1.3	Did baseline differences between intervention groups suggest a problem with the randomisation process?<br>***Note:*** *Baseline characteristics were similar between groups with no apparent imbalances.*	N
**Risk-of-bias judgment**		Low
**2**	**Bias due to deviations from intended interventions**	
2.1	Were participants aware of their assigned intervention during the trial?<br>***Note:*** *Participants were aware of the intervention they received as the trial was not blinded.*	Y
2.2	Were carers and people delivering the interventions aware of participants' assigned intervention during the trial?<br>***Note:*** *Healthcare providers and personnel delivering interventions were aware of group assignment.*	Y
2.3	If Y/PY/NI to 2.1 or 2.2: Were there deviations from the intended intervention that arose because of the trial context?<br>***Note:*** *There was no indication of deviations from intended intervention due to trial context.*	N
2.4	If Y/PY/NI to 2.3: Were these deviations likely to have affected the outcome?	NA
2.5	If Y/PY to 2.4: Were these deviations from intended intervention balanced between groups?	NA
2.6	Was an appropriate analysis used to estimate the effect of assignment to intervention?<br>***Note:*** *Intention-to-treat analysis was used to compare outcomes.*	Y
2.7	If N/PN/NI to 2.6: Was there potential for a substantial impact (on the result) of the failure to analyse participants in the group to which they were randomised?	NA
**Risk-of-bias judgment**		Low
**3**	**Bias due to missing outcome data**	
3.1	Were data for this outcome available for all, or nearly all, participants randomised?<br>***Note:*** *Although all 168 randomized patients were included in the ITT analysis (84 per group), 3 (RFA group) and 4 (RES group) were lost to follow-up.*	PY
3.2	If N/PN/NI to 3.1: Is there evidence that the result was not biased by missing outcome data?	NA
3.3	If N/PN to 3.2: Could missingness in the outcome depend on its true value?	NA
3.4	If Y/PY/NI to 3.3: Is it likely that missingness in the outcome depended on its true value?	NA
**Risk-of-bias judgment**		Low
**4**	**Bias in measurement of the outcome**	
4.1	Was the method of measuring the outcome inappropriate?	N

Feng2012 *(continued)*

	Note: *The outcome (local tumor progression and survival) was assessed via imaging and records, appropriate and objective methods.*	
4.2	Could measurement or ascertainment of the outcome have differed between intervention groups?	N
	Note: *Same imaging and follow-up protocol applied to both groups.*	
4.3	If N/PN/NI to 4.1 and 4.2: Were outcome assessors aware of the intervention received by study participants?	Y
	Note: *Outcome assessors were likely aware of group allocation due to open-label design.*	
4.4	If Y/PY/NI to 4.3: Could assessment of the outcome have been influenced by knowledge of intervention received?	N
	Note: *Knowledge of intervention was unlikely to influence assessment of objective imaging-based outcomes.*	
4.5	If Y/PY/NI to 4.4: Is it likely that assessment of the outcome was influenced by knowledge of intervention received?	NA
**Risk-of-bias judgment**		Low
**5**	**Bias in selection of the reported result**	
5.1	Were the data that produced this result analysed in accordance with a prespecified analysis plan that was finalised before unblinded outcome data were available for analysis?	PY
	Note: *No pre-specified protocol or analysis plan was mentioned, nor was the registration. However, considering the time of patients enrollment (from 2005 to 2008, when the registration platform was not that accessible) and the precise description of Methods of this study, we didn't suppose that there was a significant deviation from the prespecified analysis.*	
Is the numerical result being assessed likely to have been selected, on the basis of the results, from:		
5.2	Multiple eligible outcome measurements (eg, scales, definitions, time points) within the outcome domain?	N
	Note: *There was only one pre-specified measurement approach per outcome.*	
5.3	Multiple eligible analyses of the data?	N
	Note: *Only one analysis strategy was applied per outcome.*	
**Risk-of-bias judgment**		Low
**Overall bias**		Low

Féray2023

**Title:** TACE and conformal radiotherapy vs. TACE alone for hepatocellular carcinoma: A randomised controlled trial		
**Authors:** Cyrille Féray, Loic Campion, Philippe Mathurin, *et al.*		**Year:** 2023
**Interventions:** Transcatheter arterial chemoembolization (TACE) + Radiotherapy (RT) vs. TACE		
**Registration:** NCT01300143		
**1**	**Bias arising from the randomisation process**	
1.1	Was the allocation sequence random?	Y
	Note: *The authors state that randomization was performed using "a stratified permuted block (n = 4) procedure" with specific stratification factors, indicating a proper random allocation sequence.*	
1.2	Was the allocation sequence concealed until participants were enrolled and assigned to interventions?	PY
	Note: *Stratified permuted block is used, indicating the randomization process.*	
1.3	Did baseline differences between intervention groups suggest a problem with the randomisation process?	PN
	Note: *Though the baseline differed between the two groups in some figures, we tended to attribute this to multiple tests. Thus, we did't think the baseline difference was significantly difference between the two groups.*	
**Risk-of-bias judgment**		Low
**2**	**Bias due to deviations from intended interventions**	
2.1	Were participants aware of their assigned intervention during the trial?	Y
	Note: *The trial was open-label; participants were aware of whether they received TACE alone or TACE + CRT.*	
2.2	Were carers and people delivering the interventions aware of participants' assigned intervention during the trial?	Y
	Note: *As an open-label trial, clinicians and caregivers were aware of the treatment assignments.*	
2.3	If Y/PY/NI to 2.1 or 2.2: Were there deviations from the intended intervention that arose because of the trial context?	PN
	Note: *Figure 1 (flowchart) indicates that several participants did not receive the allocated intervention (e.g., 4 in each arm did not receive any treatment, 3 in TACE+CRT had incomplete treatment). However, we didn't think this may bring in significant bias.*	
2.4	If Y/PY/NI to 2.3: Were these deviations likely to have affected the outcome?	NA
2.5	If Y/PY to 2.4: Were these deviations from intended intervention balanced between groups?	NA
2.6	Was an appropriate analysis used to estimate the effect of assignment to intervention?	Y
	Note: *The authors conducted both intention-to-treat and per-protocol analyses, and used propensity score weighting to adjust for imbalances.*	
2.7	If N/PN/NI to 2.6: Was there potential for a substantial impact (on the result) of the failure to analyse participants in the group to which they were randomised?	NA
**Risk-of-bias judgment**		Low
**3**	**Bias due to missing outcome data**	
3.1	Were data for this outcome available for all, or nearly all, participants randomised?	Y
	Note: *The loss to follow-up was very low (only 2 participants in each arm were lost to follow-up), and the authors used a national mortality database to confirm dates of death.*	
3.2	If N/PN/NI to 3.1: Is there evidence that the result was not biased by missing outcome data?	NA
3.3	If N/PN to 3.2: Could missingness in the outcome depend on its true value?	NA

Féray2023 *(continued)*

3.4	If Y/PY/NI to 3.3: Is it likely that missingness in the outcome depended on its true value?	NA
**Risk-of-bias judgment**		Low
**4**	**Bias in measurement of the outcome**	
4.1	Was the method of measuring the outcome inappropriate?<br>***Note:*** *Overall survival, progression-free survival, and adverse events were measured using standard and appropriate methods (mRECIST, NCI CTCAE v4.0, national mortality database).*	N
4.2	Could measurement or ascertainment of the outcome have differed between intervention groups?<br>***Note:*** *Although the trial was open-label, the primary outcomes (survival, progression) are objective and based on imaging and registry data, reducing the likelihood of differential measurement.*	PN
4.3	If N/PN/NI to 4.1 and 4.2: Were outcome assessors aware of the intervention received by study participants?<br>***Note:*** *Local assessors were likely aware of the treatment assignment. However, a centralized blinded imaging review was also performed for a subset of patients.*	Y
4.4	If Y/PY/NI to 4.3: Could assessment of the outcome have been influenced by knowledge of intervention received?<br>***Note:*** *The use of a centralized blinded review for imaging outcomes mitigates the risk of bias, though local assessments may have been influenced.*	PN
4.5	If Y/PY/NI to 4.4: Is it likely that assessment of the outcome was influenced by knowledge of intervention received?	NA
**Risk-of-bias judgment**		Low
**5**	**Bias in selection of the reported result**	
5.1	Were the data that produced this result analysed in accordance with a prespecified analysis plan that was finalised before unblinded outcome data were available for analysis?<br>***Note:*** *The trial was registered (NCT01300143). Though some minimal difference in description (e.g., the primary endpoint was erroneously listed as "time to progression" on ClinicalTrials.gov, while the protocol specified "liver progression-free survival"), no significant difference between the protocol and the results was observed.*	PY
Is the numerical result being assessed likely to have been selected, on the basis of the results, from:		
5.2	Multiple eligible outcome measurements (eg, scales, definitions, time points) within the outcome domain?<br>***Note:*** *The authors reported both ITT and per-protocol analyses, as well as propensity score-adjusted results, but there is no strong evidence of selective reporting from multiple outcome measures.*	PN
5.3	Multiple eligible analyses of the data?<br>***Note:*** *The statistical methods appear pre-specified and standard (Kaplan-Meier, Cox regression, propensity score analysis). There is no indication of analysis shopping.*	PN
**Risk-of-bias judgment**		Low
**Overall bias**		Low

Ferrari2007

**Title:** Treatment of small HCC through radiofrequency ablation and laser ablation. Comparison of techniques and long-term results		
**Authors:** F.S. Ferrari, A. Megliola, A. Scorzelli *et al.*.		**Year:** 2007
**Interventions:** Radiofrequency ablation (RFA) vs Laser ablation (LA)		
**Registration number:** NA		
**1**	**Bias arising from the randomisation process**	
1.1	Was the allocation sequence random?	Y
	Note: *The paper states that "Patients were randomly allocated to the treatment groups by means of randomisation software." This is acceptable evidence that a random allocation sequence was generated.*	
1.2	Was the allocation sequence concealed until participants were enrolled and assigned to interventions?	PY
	Note: *No evidence indicates a disclosure of allocation sequence.*	
1.3	Did baseline differences between intervention groups suggest a problem with the randomisation process?	PN
	Note: *The authors state that the treatment groups were homogeneous with respect to HCC nodule size, clinical/laboratory conditions, Child-Pugh distribution, age, and sex ratio. The baseline table also does not show any obviously extreme imbalance. On a lenient reading, this does not suggest a serious problem with randomization.*	
**Risk-of-bias judgment**		Low
**2**	**Bias due to deviations from intended interventions**	
2.1	Were participants aware of their assigned intervention during the trial?	PY
	Note: *The study compared two visibly different interventional procedures, laser ablation (LA) and radiofrequency ablation (RFA), both performed under general anaesthesia and ultrasound guidance, but with clearly different devices and procedural characteristics. It is therefore likely that participants were aware, at least to some extent, of which intervention they received.*	
2.2	Were carers and people delivering the interventions aware of participants' assigned intervention during the trial?	Y
	Note: *The operators clearly knew which procedure they were delivering, because LA and RFA involved different equipment, applicators, and treatment protocols.*	
2.3	If Y/PY/NI to 2.1 or 2.2: Were there deviations from the intended intervention that arose because of the trial context?	PN
	Note: *There is no clear evidence that patients or clinicians deviated from the assigned intervention because of lack of blinding. Repeated treatment sessions were built into the management strategy and were part of the therapeutic protocol rather than obvious protocol-breaking departures.*	
2.4	If Y/PY/NI to 2.3: Were these deviations likely to have affected the outcome?	NA
2.5	If Y/PY to 2.4: Were these deviations from intended intervention balanced between groups?	NA
2.6	Was an appropriate analysis used to estimate the effect of assignment to intervention?	PY
	Note: *This study follows ITT principle in the report, though it's not specifically declared.*	
2.7	If N/PN/NI to 2.6: Was there potential for a substantial impact (on the result) of the failure to analyse participants in the group to which they were randomised?	NA
**Risk-of-bias judgment**		Low
**3**	**Bias due to missing outcome data**	

Ferrari2007 *(continued)*

3.1	Were data for this outcome available for all, or nearly all, participants randomised? ***Note:*** *For the early complete ablation outcome, the paper suggests that all treated nodules were assessed with post-treatment CT within 15 days, and the complete ablation result is reported for all 95 nodules. This implies outcome availability was close to complete for the initial efficacy endpoint.*	PY
3.2	If N/PN/NI to 3.1: Is there evidence that the result was not biased by missing outcome data?	NA
3.3	If N/PN to 3.2: Could missingness in the outcome depend on its true value?	NA
3.4	If Y/PY/NI to 3.3: Is it likely that missingness in the outcome depended on its true value?	NA
**Risk-of-bias judgment**		Low
**4**	**Bias in measurement of the outcome**	
4.1	Was the method of measuring the outcome inappropriate? ***Note:*** *Complete tumour ablation was assessed by triphasic CT, with clear radiologic criteria: absence of contrast uptake in the ablation zone during the early arterial phase indicated complete ablation. This is an appropriate measurement method for local treatment efficacy in HCC.*	N
4.2	Could measurement or ascertainment of the outcome have differed between intervention groups? ***Note:*** *The same CT-based assessment framework appears to have been applied to both LA and RFA groups. The imaging schedule was also described similarly for all patients.*	PN
4.3	If N/PN/NI to 4.1 and 4.2: Were outcome assessors aware of the intervention received by study participants? ***Note:*** *Outcome assessors could be aware of the intervention, because they're both surgical interventions.*	PY
4.4	If Y/PY/NI to 4.3: Could assessment of the outcome have been influenced by knowledge of intervention received? ***Note:*** *All the outcomes (survival or complications) included in this systematic review were relatively objective, so we deem the assessment couldn't be influenced by knowledge of intervention received.*	PN
4.5	If Y/PY/NI to 4.4: Is it likely that assessment of the outcome was influenced by knowledge of intervention received?	NA
**Risk-of-bias judgment**		Low
**5**	**Bias in selection of the reported result**	
5.1	Were the data that produced this result analysed in accordance with a prespecified analysis plan that was finalised before unblinded outcome data were available for analysis? ***Note:*** *This study was not registered. Considering that the recruitment of patients were finished before 2005, when registration just started to be widely applied, we don't think it's rational to downgrade this study in this field. Additionally, the Results followed the Methods part well, so we suppose the result was analyzed in accordance with prespecified analysis plan.*	PY
Is the numerical result being assessed likely to have been selected, on the basis of the results, from:		
5.2	Multiple eligible outcome measurements (eg, scales, definitions, time points) within the outcome domain? ***Note:*** *The outcomes were clearly defined. So although multiple outcomes exist, the measurements were identical.*	N
5.3	Multiple eligible analyses of the data? ***Note:*** *No evidence suggest multiple analyses of the data.*	N

Ferrari2007 *(continued)*

**Risk-of-bias judgment**	Low
**Overall bias**	Low

Giorgio2011

Title: Percutaneous Radiofrequency Ablation of Hepatocellular Carcinoma Compared to Percutaneous Ethanol Injection in Treatment of Cirrhotic Patients: An Italian Randomized Controlled Trial

Authors: A. Giorgio, A. Di Sarno, G. De Stefano, *et al.* **Year:** 2011

Interventions: Percutaneous ethanol injection (PEI) vs. Radiofrequency ablation (RFA)

Registration: NA

**1**	**Bias arising from the randomisation process**	
1.1	Was the allocation sequence random?<br>***Note:*** *The authors state that randomization was performed "prospectively with the use of a coded list compiled from a random number generator", which confirms that the allocation sequence was random.*	Y
1.2	Was the allocation sequence concealed until participants were enrolled and assigned to interventions?<br>***Note:*** *The code was "fully blinded to the field staff", which suggests concealment was attempted, but it is not explicitly stated that the sequence was concealed until after enrollment and assignment.*	PY
1.3	Did baseline differences between intervention groups suggest a problem with the randomisation process?<br>***Note:*** *Table 1 shows no statistically significant differences in baseline characteristics between the PEI and RFA groups, indicating that the randomization process was successful.*	N
**Risk-of-bias judgment**		Low
**2**	**Bias due to deviations from intended interventions**	
2.1	Were participants aware of their assigned intervention during the trial?<br>***Note:*** *This is a percutaneous intervention trial; participants would likely be aware of the type of procedure they received due to the nature of the treatment.*	Y
2.2	Were carers and people delivering the interventions aware of participants' assigned intervention during the trial?<br>***Note:*** *The same physician performed all procedures, so the intervention provider was aware of the treatment assignment.*	Y
2.3	If Y/PY/NI to 2.1 or 2.2: Were there deviations from the intended intervention that arose because of the trial context?<br>***Note:*** *The deviations might exist, but not deviate because of the trial context. All the outcomes were objective.*	PN
2.4	If Y/PY/NI to 2.3: Were these deviations likely to have affected the outcome?	NA
2.5	If Y/PY to 2.4: Were these deviations from intended intervention balanced between groups?	NA
2.6	Was an appropriate analysis used to estimate the effect of assignment to intervention?<br>***Note:*** *The authors used intention-to-treat analysis for survival outcomes, which is appropriate for estimating the effect of assignment to intervention.*	Y
2.7	If N/PN/NI to 2.6: Was there potential for a substantial impact (on the result) of the failure to analyse participants in the group to which they were randomised?	NA
**Risk-of-bias judgment**		Low
**3**	**Bias due to missing outcome data**	
3.1	Were data for this outcome available for all, or nearly all, participants randomised?<br>***Note:*** *4 patients randomized to RFA were switched to PEI due to technical feasibility and were excluded from the survival analysis.*	N

**Giorgio2011** *(continued)*		
3.2	If N/PN/NI to 3.1: Is there evidence that the result was not biased by missing outcome data?<br>***Note:*** *The exclusion was due to technical reasons (segment location), which may introduce bias, as these patients might have had worse outcomes.*	PN
3.3	If N/PN to 3.2: Could missingness in the outcome depend on its true value?<br>***Note:*** *The missingness was related to tumor location, which could be associated with prognosis.*	Y
3.4	If Y/PY/NI to 3.3: Is it likely that missingness in the outcome depended on its true value?<br>***Note:*** *Tumors in difficult locations (e.g., caudate lobe) may be associated with higher risk of recurrence or worse survival, suggesting that missingness may depend on the true outcome value.*	PN
**Risk-of-bias judgment**		High
**4**	**Bias in measurement of the outcome**	
4.1	Was the method of measuring the outcome inappropriate?<br>***Note:*** *Survival and local recurrence were measured using standard clinical and imaging follow-up (US, CT), which are appropriate for HCC.*	N
4.2	Could measurement or ascertainment of the outcome have differed between intervention groups?<br>***Note:*** *The same follow-up protocol (US every 2 months, enhanced CT at 1 month) was applied to both groups.*	PN
4.3	If N/PN/NI to 4.1 and 4.2: Were outcome assessors aware of the intervention received by study participants?<br>***Note:*** *Considering the open-label nature of this study, we supposed that the assessors were aware of the intervention received by study participants.*	PY
4.4	If Y/PY/NI to 4.3: Could assessment of the outcome have been influenced by knowledge of intervention received?<br>***Note:*** *Not specified, but since the outcomes are objective (survival, recurrence on imaging), blinding may be less critical.*	NI
4.5	If Y/PY/NI to 4.4: Is it likely that assessment of the outcome was influenced by knowledge of intervention received?<br>***Note:*** *Given the objective nature of the outcomes (mortality and radiologically confirmed recurrence), it is unlikely that knowledge of the intervention significantly influenced the assessment.*	PN
**Risk-of-bias judgment**		Some concerns
**5**	**Bias in selection of the reported result**	
5.1	Were the data that produced this result analysed in accordance with a prespecified analysis plan that was finalised before unblinded outcome data were available for analysis?<br>***Note:*** *There is no mention of a preregistered protocol or statistical analysis plan.*	N
Is the numerical result being assessed likely to have been selected, on the basis of the results, from:		
5.2	Multiple eligible outcome measurements (eg, scales, definitions, time points) within the outcome domain?<br>***Note:*** *The primary outcome (5-year survival) was clearly defined, and there is no evidence of selective reporting from multiple measurements.*	N
5.3	Multiple eligible analyses of the data?<br>***Note:*** *The authors used standard survival analysis methods (Kaplan-Meier, log-rank test, Cox regression), and there is no indication of multiple analytic approaches.*	N
**Risk-of-bias judgment**		Low
**Overall bias**		High

Gjoreski2021

**Title:** Single-center randomized trial comparing conventional chemoembolization versus doxorubicin-loaded polyethylene glycol microspheres for early- and intermediate-stage hepatocellular carcinoma		
**Authors:** Aleksandar Gjoreski, Ivona Jovanoska, Filip Risteski, *et al.*		**Year:** 2021
**Interventions:** Transcatheter arterial chemoembolization (TACE) vs. Drug-eluting microspheres TACE (DEM-TACE)		
**Registration:** NA		
**1**	**Bias arising from the randomisation process**	
1.1	Was the allocation sequence random?<br>***Note:*** *The authors state that patients were "randomized one-to-one" to undergo c-TACE or DEM-TACE, confirming a random allocation sequence.*	Y
1.2	Was the allocation sequence concealed until participants were enrolled and assigned to interventions?<br>***Note:*** *This is a randomized trial, and the authors indicated that the allocation sequence was concealed.*	PY
1.3	Did baseline differences between intervention groups suggest a problem with the randomisation process?<br>***Note:*** *Table 1 shows no statistically significant differences in baseline clinical or tumor characteristics between the c-TACE and DEM-TACE groups, indicating the randomization process was likely successful.*	N
**Risk-of-bias judgment**		Low
**2**	**Bias due to deviations from intended interventions**	
2.1	Were participants aware of their assigned intervention during the trial?<br>***Note:*** *This is a procedural intervention trial; participants would likely be aware of the type of TACE procedure they received due to the nature of the treatment and post-procedural care.*	Y
2.2	Were carers and people delivering the interventions aware of participants' assigned intervention during the trial?<br>***Note:*** *The interventional radiologists performing the procedures were necessarily aware of the treatment assignment to administer the correct technique (c-TACE or DEM-TACE).*	Y
2.3	If Y/PY/NI to 2.1 or 2.2: Were there deviations from the intended intervention that arose because of the trial context?<br>***Note:*** *The authors do not report any deviations from the intended interventions that occurred specifically due to the trial context.*	NI
2.4	If Y/PY/NI to 2.3: Were these deviations likely to have affected the outcome?	NA
2.5	If Y/PY to 2.4: Were these deviations from intended intervention balanced between groups?	NA
2.6	Was an appropriate analysis used to estimate the effect of assignment to intervention?<br>***Note:*** *The authors used Kaplan-Meier survival analysis and log-rank tests to compare groups based on their original random assignment (intention-to-treat principle), which is appropriate.*	Y
2.7	If N/PN/NI to 2.6: Was there potential for a substantial impact (on the result) of the failure to analyse participants in the group to which they were randomised?	NA
**Risk-of-bias judgment**		Some concerns
**3**	**Bias due to missing outcome data**	
3.1	Were data for this outcome available for all, or nearly all, participants randomised?<br>***Note:*** *All 60 randomized patients were included in the survival analysis, and the number of events (deaths) is reported for each group.*	Y

Gjoreski2021 *(continued)*

3.2	If N/PN/NI to 3.1: Is there evidence that the result was not biased by missing outcome data?	NA
3.3	If N/PN to 3.2: Could missingness in the outcome depend on its true value?	NA
3.4	If Y/PY/NI to 3.3: Is it likely that missingness in the outcome depended on its true value?	NA
**Risk-of-bias judgment**		Low
**4**	**Bias in measurement of the outcome**	
4.1	Was the method of measuring the outcome inappropriate?	N
	Note: *Overall survival is an objective outcome, and the method of measurement (time from randomization to death) is appropriate.*	
4.2	Could measurement or ascertainment of the outcome have differed between intervention groups?	N
	Note: *The method for ascertaining death is unlikely to differ between the two treatment groups.*	
4.3	If N/PN/NI to 4.1 and 4.2: Were outcome assessors aware of the intervention received by study participants?	NI
	Note: *The article does not specify whether the individuals assessing survival (e.g., those collecting follow-up data) were blinded to the treatment group.*	
4.4	If Y/PY/NI to 4.3: Could assessment of the outcome have been influenced by knowledge of intervention received?	PN
	Note: *The outcome (death) is objective and not subject to interpretation, making it unlikely that knowledge of the intervention influenced its assessment.*	
4.5	If Y/PY/NI to 4.4: Is it likely that assessment of the outcome was influenced by knowledge of intervention received?	NA
**Risk-of-bias judgment**		Low
**5**	**Bias in selection of the reported result**	
5.1	Were the data that produced this result analysed in accordance with a prespecified analysis plan that was finalised before unblinded outcome data were available for analysis?	N
	Note: *There is no mention of a preregistered protocol or statistical analysis plan (e.g., on a trials registry).*	
Is the numerical result being assessed likely to have been selected, on the basis of the results, from:		
5.2	Multiple eligible outcome measurements (eg, scales, definitions, time points) within the outcome domain?	N
	Note: *The primary outcome (12- and 24-month survival) was clearly defined prospectively, and there is no evidence of selective reporting from multiple measurements.*	
5.3	Multiple eligible analyses of the data?	N
	Note: *The authors used standard survival analysis methods (Kaplan-Meier, log-rank test, Cox regression), and there is no indication of multiple analytic approaches being tried.*	
**Risk-of-bias judgment**		Some concerns
**Overall bias**		Some concerns

Huang2005

Title: Percutaneous Ethanol Injection Versus Surgical Resection for the Treatment of Small Hepatocellular Carcinoma: A Prospective Study

Authors: Guantarn Huang, Pohuang Lee, Yukming Tsang *et al.*. **Year:** 2005

Interventions: Percutaneous ethanol injection (PEI) vs Surgical resection (SR)

Registration number: NA

**1**	**Bias arising from the randomisation process**	
1.1	Was the allocation sequence random?<br>***Note:*** *The paper states that treatment assignment was determined by use of "a random table." This is an acceptable random sequence generation method for a trial of this period.*	Y
1.2	Was the allocation sequence concealed until participants were enrolled and assigned to interventions?<br>***Note:*** *The article reports the use of a random table, but it does not clearly describe allocation concealment procedures such as central randomization or sealed opaque envelopes. However, given the age of the study and the prospective design, it's not rational to over-penalize this unless there were signs of manipulation.*	PY
1.3	Did baseline differences between intervention groups suggest a problem with the randomisation process?<br>***Note:*** *Baseline characteristics were broadly comparable. The paper notes that, except for gender ratio, viral markers, and number of tumors, no other parameters were statistically different. The table does show some imbalance, such as 3 Child B patients in the PEIT group and 0 in the resection group, but under a lenient interpretation this still does not strongly suggest failure of the randomization process.*	PN
**Risk-of-bias judgment**		Low
**2**	**Bias due to deviations from intended interventions**	
2.1	Were participants aware of their assigned intervention during the trial?<br>***Note:*** *Participants would clearly know whether they underwent surgical resection or percutaneous ethanol injection therapy, since these are very different procedures.*	Y
2.2	Were carers and people delivering the interventions aware of participants' assigned intervention during the trial?<br>***Note:*** *Clinicians necessarily knew which treatment they delivered because surgery and PEIT are fundamentally different interventions.*	Y
2.3	If Y/PY/NI to 2.1 or 2.2: Were there deviations from the intended intervention that arose because of the trial context?<br>***Note:*** *The paper does not report systematic departures from assigned treatment after randomization among the 76 analyzed patients. Although 6 patients were excluded before final analysis, the article explains these were handled before inclusion in the analyzed set, and replacement patients were enrolled. In a modern trial this is not ideal, but under a more tolerant reading, the study still appears to have compared the intended strategies prospectively.*	PN
2.4	If Y/PY/NI to 2.3: Were these deviations likely to have affected the outcome?	NA
2.5	If Y/PY to 2.4: Were these deviations from intended intervention balanced between groups?	NA
2.6	Was an appropriate analysis used to estimate the effect of assignment to intervention?	PY

	Note: *The paper analyzes survival using Kaplan–Meier methods and Cox regression, and the final analyzed groups are equal in size (38 vs 38). It does not explicitly use the term "intention-to-treat," but for survival comparison in this older-style randomized clinical study, the analysis appears broadly appropriate.*	
2.7	If N/PN/NI to 2.6: Was there potential for a substantial impact (on the result) of the failure to analyse participants in the group to which they were randomised?	NA
**Risk-of-bias judgment**		Low
**3**	**Bias due to missing outcome data**	
3.1	Were data for this outcome available for all, or nearly all, participants randomised?	PY
	Note: *The paper states that all enrolled patients had been followed for at least 1 year, and follow-up duration ranged from 12 to 59 months. Survival status appears to have been adequately tracked for the analyzed population.*	
3.2	If N/PN/NI to 3.1: Is there evidence that the result was not biased by missing outcome data?	NA
3.3	If N/PN to 3.2: Could missingness in the outcome depend on its true value?	NA
3.4	If Y/PY/NI to 3.3: Is it likely that missingness in the outcome depended on its true value?	NA
**Risk-of-bias judgment**		Low
**4**	**Bias in measurement of the outcome**	
4.1	Was the method of measuring the outcome inappropriate?	N
	Note: *The outcome assessed here is overall survival, which is an objective and appropriate clinical endpoint.*	
4.2	Could measurement or ascertainment of the outcome have differed between intervention groups?	PN
	Note: *Death is an objective outcome and is unlikely to have been measured differently across PEIT and resection groups.*	
4.3	If N/PN/NI to 4.1 and 4.2: Were outcome assessors aware of the intervention received by study participants?	PY
	Note: *The study does not mention blinding, and in practice the investigators were likely aware of the treatment assignment.*	
4.4	If Y/PY/NI to 4.3: Could assessment of the outcome have been influenced by knowledge of intervention received?	PN
	Note: *For overall survival, assessor knowledge is unlikely to materially influence whether death occurred.*	
4.5	If Y/PY/NI to 4.4: Is it likely that assessment of the outcome was influenced by knowledge of intervention received?	NA
**Risk-of-bias judgment**		Low
**5**	**Bias in selection of the reported result**	
5.1	Were the data that produced this result analysed in accordance with a prespecified analysis plan that was finalised before unblinded outcome data were available for analysis?	PY
	Note: *This study was not registered. Considering that the recruitment of patients were finished before 2005, when registration just started to be widely applied, we don't think it's rational to downgrade this study in this field. Additionally, the Results followed the Methods part well, so we suppose the result was analyzed in accordance with prespecified analysis plan.*	
Is the numerical result being assessed likely to have been selected, on the basis of the results, from:		

Huang2005 *(continued)*

5.2	Multiple eligible outcome measurements (eg, scales, definitions, time points) within the outcome domain?<br>***Note:*** *For survival, the outcome is relatively straightforward. The article reports survival and tumor-free survival, but these are conceptually distinct endpoints rather than many interchangeable measures of the same exact outcome.*	PN
5.3	Multiple eligible analyses of the data?<br>***Note:*** *The paper uses standard survival methods and does not appear to present many competing alternative analyses of overall survival. Although subgroup and regression analyses are reported, there is not strong evidence of selective emphasis for the main survival comparison.*	PN
**Risk-of-bias judgment**		Low
**Overall bias**		Low

Huang2010

Title: A Randomized Trial Comparing Radiofrequency Ablation and Surgical Resection for HCC Conforming to the Milan Criteria

Authors: Jiwei Huang, Lvnan Yan, Zheyu Cheng *et al.*. **Year:** 2005

Interventions: Radiofrequency ablation (RFA) vs Surgical resection (SR)

Registration number: ChiCTR-TRC-00000372

**1**	**Bias arising from the randomisation process**	
1.1	Was the allocation sequence random?<br>***Note:*** *The paper states that a statistician assigned patients to the 2 groups "by a blocking/stratification randomization method with a computer." This is adequate evidence that the allocation sequence was random.*	Y
1.2	Was the allocation sequence concealed until participants were enrolled and assigned to interventions?<br>***Note:*** *The article describes a clear concealment process: registry numbers were printed on envelopes in order, the corresponding group assignment was sealed in each envelope, physicians received the envelopes in sequence, and the randomized sequence was known only by the statistician. This is good evidence of allocation concealment.*	PY
1.3	Did baseline differences between intervention groups suggest a problem with the randomisation process?<br>***Note:*** *Most baseline characteristics were similar between groups. The authors state that baseline characteristics were not significantly different overall, although there was an imbalance in solitary tumor size, with smaller solitary tumors more common in the RFA group. Under a lenient interpretation, this isolated imbalance does not clearly indicate a failure of the randomization process.*	PN
**Risk-of-bias judgment**		Low
**2**	**Bias due to deviations from intended interventions**	
2.1	Were participants aware of their assigned intervention during the trial?<br>***Note:*** *Participants would clearly know whether they underwent radiofrequency ablation or surgical resection, since these are very different procedures in terms of invasiveness, hospitalization, and perioperative experience.*	Y
2.2	Were carers and people delivering the interventions aware of participants' assigned intervention during the trial?<br>***Note:*** *Clinicians necessarily knew which intervention they were delivering, because RFA and surgical resection are fundamentally different procedures. The paper also explicitly states that a double-blind design was not used because of the nature of the interventions.*	Y
2.3	If Y/PY/NI to 2.1 or 2.2: Were there deviations from the intended intervention that arose because of the trial context?<br>***Note:*** *7 patients assigned to RFA withdrew consent after the intervention was revealed and chose surgery instead. However, considering the large sample size, we suppose the withdrawal won't contribute significant deviation to the results.*	PN
2.4	If Y/PY/NI to 2.3: Were these deviations likely to have affected the outcome?	NA
2.5	If Y/PY to 2.4: Were these deviations from intended intervention balanced between groups?	NA
2.6	Was an appropriate analysis used to estimate the effect of assignment to intervention?	Y

Huang2010 *(continued)*

	Note: *The paper explicitly states that "All analyses were done on the intention-to-treat basis." In addition, the results section confirms that all 115 assigned to each arm were included in the intention-to-treat analysis for survival and recurrence. This is exactly the correct analysis strategy for the ITT estimand.*	
2.7	If N/PN/NI to 2.6: Was there potential for a substantial impact (on the result) of the failure to analyse participants in the group to which they were randomised?	NA
**Risk-of-bias judgment**		Low
**3**	**Bias due to missing outcome data**	
3.1	Were data for this outcome available for all, or nearly all, participants randomised?	PY
	Note: *Considering the large sample size, only 25 lost to follow-ups won't influence the results significantly.*	
3.2	If N/PN/NI to 3.1: Is there evidence that the result was not biased by missing outcome data?	NA
3.3	If N/PN to 3.2: Could missingness in the outcome depend on its true value?	NA
3.4	If Y/PY/NI to 3.3: Is it likely that missingness in the outcome depended on its true value?	NA
**Risk-of-bias judgment**		Low
**4**	**Bias in measurement of the outcome**	
4.1	Was the method of measuring the outcome inappropriate?	N
	Note: *Death is an objective outcome, and the same follow-up framework was applied to both groups. It is unlikely that ascertainment of death differed materially between groups*	
4.2	Could measurement or ascertainment of the outcome have differed between intervention groups?	PN
	Note: *Death is an objective outcome, and the same follow-up framework was applied to both groups. It is unlikely that ascertainment of death differed materially between groups.*	
4.3	If N/PN/NI to 4.1 and 4.2: Were outcome assessors aware of the intervention received by study participants?	PY
	Note: *The trial was not double-blind, and investigators were likely aware of treatment assignment during follow-up.*	
4.4	If Y/PY/NI to 4.3: Could assessment of the outcome have been influenced by knowledge of intervention received?	PN
	Note: *Because the outcome is overall survival, assessor knowledge is very unlikely to influence whether death occurred.*	
4.5	If Y/PY/NI to 4.4: Is it likely that assessment of the outcome was influenced by knowledge of intervention received?	NA
**Risk-of-bias judgment**		Low
**5**	**Bias in selection of the reported result**	
5.1	Were the data that produced this result analysed in accordance with a prespecified analysis plan that was finalised before unblinded outcome data were available for analysis?	PY
	Note: *The trial was prospectively registered, the primary and secondary endpoints were explicitly defined, the sample size calculation was based on the primary endpoint, and the statistical methods were described in advance in the report. Although the full statistical analysis plan is not separately provided, this is stronger reporting than in many older trials. Under a lenient interpretation, this supports a "probably yes" answer.*	
Is the numerical result being assessed likely to have been selected, on the basis of the results, from:		

Huang2010 *(continued)*

5.2	Multiple eligible outcome measurements (eg, scales, definitions, time points) within the outcome domain?<br>***Note:*** *For the outcome domain of overall survival, the study is straightforward. The primary endpoint was clearly defined as overall survival, rather than several interchangeable survival measures.*	PN
5.3	Multiple eligible analyses of the data?<br>***Note:*** *The main survival analysis used standard Kaplan-Meier and log-rank methods, supplemented by Cox regression. Although subgroup analyses were also reported, the primary comparison itself seems clearly prespecified and prominently reported.*	PN
**Risk-of-bias judgment**		Low
**Overall bias**		Low

Kamal2019

Title: Percutaneous radiofrequency versus microwave ablation for management of hepatocellular carcinoma: a randomized controlled trial

Authors: Ahmed Kamal, Amr Aly Abd Elmoety, Yousri Abdel Meguid Rostom, *et al.* **Year:** 2019

Interventions: Radiofrequency ablation (RFA) vs. microwave ablation (MWA)

Registration: NA

**1**	**Bias arising from the randomisation process**	
1.1	Was the allocation sequence random?<br>***Note:*** *The authors state that "patients were randomly assigned to either RFA or MWA", and the CONSORT flow diagram (Figure 1) labels the process as "Randomized," confirming a random allocation sequence.*	Y
1.2	Was the allocation sequence concealed until participants were enrolled and assigned to interventions?<br>***Note:*** *The patients were randomized, and no evidence suggested that the patients were aware of the concealment before getting enrolled in the study.*	PY
1.3	Did baseline differences between intervention groups suggest a problem with the randomisation process?<br>***Note:*** *Tables 1, 2, and 3 show no statistically significant differences in baseline demographic, clinical, laboratory, or tumor characteristics between the RFA and MWA groups, indicating the randomization process was successful.*	N
**Risk-of-bias judgment**		Low
**2**	**Bias due to deviations from intended interventions**	
2.1	Were participants aware of their assigned intervention during the trial?<br>***Note:*** *This is a percutaneous ablation trial; participants would likely be aware of the type of procedure they received due to the nature of the treatment.*	Y
2.2	Were carers and people delivering the interventions aware of participants' assigned intervention during the trial?<br>***Note:*** *The interventional radiologists performing the procedures were necessarily aware of the treatment assignment to administer the correct technique (RFA or MWA).*	Y
2.3	If Y/PY/NI to 2.1 or 2.2: Were there deviations from the intended intervention that arose because of the trial context?<br>***Note:*** *The authors do not report any deviations from the intended interventions that occurred specifically due to the trial context.*	PN
2.4	If Y/PY/NI to 2.3: Were these deviations likely to have affected the outcome?	NA
2.5	If Y/PY to 2.4: Were these deviations from intended intervention balanced between groups?	NA
2.6	Was an appropriate analysis used to estimate the effect of assignment to intervention?<br>***Note:*** *The authors used Kaplan-Meier survival analysis and log-rank tests to compare groups based on their original random assignment (intention-to-treat principle), which is appropriate.*	Y
2.7	If N/PN/NI to 2.6: Was there potential for a substantial impact (on the result) of the failure to analyse participants in the group to which they were randomised?	NA
**Risk-of-bias judgment**		Low
**3**	**Bias due to missing outcome data**	
3.1	Were data for this outcome available for all, or nearly all, participants randomised?	N

Kamal2019 *(continued)*

	Note: *Figure 1 (CONSORT diagram) shows that 6 patients (2 lost to follow-up, 4 died) in the RFA group and 7 patients (2 lost to follow-up, 5 died) in the MWA group did not complete the 12-month follow-up. Outcome data (e.g., recurrence) were therefore not available for all randomized participants.*	
3.2	If N/PN/NI to 3.1: Is there evidence that the result was not biased by missing outcome data?	PY
	Note: *The lost to follow-up seemed to be balanced between the two groups, which indicated that the true value may not be biased.*	
3.3	If N/PN to 3.2: Could missingness in the outcome depend on its true value?	NA
3.4	If Y/PY/NI to 3.3: Is it likely that missingness in the outcome depended on its true value?	NA
**Risk-of-bias judgment**		Low
**4**	**Bias in measurement of the outcome**	
4.1	Was the method of measuring the outcome inappropriate?	N
	Note: *Local tumor recurrence was assessed using triphasic CT and evaluated according to mRECIST criteria, which is an appropriate and standard method.*	
4.2	Could measurement or ascertainment of the outcome have differed between intervention groups?	N
	Note: *The same follow-up protocol and imaging criteria (triphasic CT, mRECIST) were applied uniformly to both groups.*	
4.3	If N/PN/NI to 4.1 and 4.2: Were outcome assessors aware of the intervention received by study participants?	NI
	Note: *The article does not specify whether the radiologists assessing the CT scans for recurrence were blinded to the treatment group.*	
4.4	If Y/PY/NI to 4.3: Could assessment of the outcome have been influenced by knowledge of intervention received?	PN
	Note: *The outcome (radiologically confirmed recurrence) is objective and based on standardized criteria (mRECIST), making it unlikely that assessor knowledge influenced the result.*	
4.5	If Y/PY/NI to 4.4: Is it likely that assessment of the outcome was influenced by knowledge of intervention received?	NA
**Risk-of-bias judgment**		Low
**5**	**Bias in selection of the reported result**	
5.1	Were the data that produced this result analysed in accordance with a prespecified analysis plan that was finalised before unblinded outcome data were available for analysis?	N
	Note: *There is no mention of a preregistered protocol or statistical analysis plan.*	
Is the numerical result being assessed likely to have been selected, on the basis of the results, from:		
5.2	Multiple eligible outcome measurements (eg, scales, definitions, time points) within the outcome domain?	N
	Note: *The primary outcomes (local recurrence, complications, survival) were clearly defined, and there is no evidence of selective reporting from multiple measurements.*	
5.3	Multiple eligible analyses of the data?	N
	Note: *The authors used standard statistical methods (Kaplan-Meier, log-rank test, Cox regression, chi-square, t-tests) as appropriate for the data types, and there is no indication of multiple analytic approaches being tried.*	
**Risk-of-bias judgment**		Some concerns
**Overall bias**		Some concerns

Koda2001

Title: Combination Therapy with Transcatheter Arterial Chemoembolization and Percutaneous Ethanol Injection Compared with Percutaneous Ethanol Injection Alone for Patients with Small Hepatocellular Carcinoma: A Randomized Control Study

Authors: Masahiko Koda, Yoshikazu Murawaki, Akeri Mitsuda, *et al.* **Year:** 2001

Interventions: A combination of transcatheter arterial chemoembolization (TACE) and percutaneous ethanol injection (PEI) vs. PEI

Registration: NA

1	**Bias arising from the randomisation process**	
1.1	Was the allocation sequence random?<br>***Note:*** *The study states that patients were randomly allocated using a computer-generated sequence.*	Y
1.2	Was the allocation sequence concealed until participants were enrolled and assigned to interventions?<br>***Note:*** *The use of "sealed envelopes" is a standard and effective method for allocation concealment, ensuring that the sequence was concealed until after enrollment and assignment.Y*	Y
1.3	Did baseline differences between intervention groups suggest a problem with the randomisation process?<br>***Note:*** *Table 1 shows no statistically significant differences in baseline characteristics between the TACE-PEI and PEI alone groups, indicating that the randomization process was successful.*	N
**Risk-of-bias judgment**		Low
2	**Bias due to deviations from intended interventions**	
2.1	Were participants aware of their assigned intervention during the trial?<br>***Note:*** *This is a procedural intervention trial; participants would be aware of whether they underwent TACE+PEI or PEI alone.*	Y
2.2	Were carers and people delivering the interventions aware of participants' assigned intervention during the trial?<br>***Note:*** *The interventional radiologists performing TACE and PEI were necessarily aware of the treatment assignment.*	Y
2.3	If Y/PY/NI to 2.1 or 2.2: Were there deviations from the intended intervention that arose because of the trial context?<br>***Note:*** *The authors do not report any deviations from the intended interventions that occurred due to the trial context.*	PN
2.4	If Y/PY/NI to 2.3: Were these deviations likely to have affected the outcome?	NA
2.5	If Y/PY to 2.4: Were these deviations from intended intervention balanced between groups?	NA
2.6	Was an appropriate analysis used to estimate the effect of assignment to intervention?<br>***Note:*** *The authors used intention-to-treat analysis (Kaplan-Meier for recurrence and survival) based on the original random assignment, which is appropriate.*	Y
2.7	If N/PN/NI to 2.6: Was there potential for a substantial impact (on the result) of the failure to analyse participants in the group to which they were randomised?	NA
**Risk-of-bias judgment**		Low
3	**Bias due to missing outcome data**	
3.1	Were data for this outcome available for all, or nearly all, participants randomised?	PY

	Note: *All 52 randomized patients were included in the analysis. Outcomes (recurrence, survival) were reported for all patients, with clear accounting of follow-up duration and events.*	
3.2	If N/PN/NI to 3.1: Is there evidence that the result was not biased by missing outcome data?	NA
3.3	If N/PN to 3.2: Could missingness in the outcome depend on its true value?	NA
3.4	If Y/PY/NI to 3.3: Is it likely that missingness in the outcome depended on its true value?	NA
**Risk-of-bias judgment**		Low
**4**	**Bias in measurement of the outcome**	
4.1	Was the method of measuring the outcome inappropriate?	N
	Note: *Local recurrence and new nodular recurrence were assessed using standard imaging (dynamic CT) and defined clearly. Survival is an objective outcome.*	
4.2	Could measurement or ascertainment of the outcome have differed between intervention groups?	N
	Note: *The same follow-up protocol (imaging, tumor markers) was applied uniformly to both groups.*	
4.3	If N/PN/NI to 4.1 and 4.2: Were outcome assessors aware of the intervention received by study participants?	PY
	Note: *The article does not specify whether the radiologists assessing recurrence were blinded to the treatment group.*	
4.4	If Y/PY/NI to 4.3: Could assessment of the outcome have been influenced by knowledge of intervention received?	PN
	Note: *The outcomes (recurrence on imaging, death) are objective and unlikely to be influenced by knowledge of the treatment group.*	
4.5	If Y/PY/NI to 4.4: Is it likely that assessment of the outcome was influenced by knowledge of intervention received?	NA
**Risk-of-bias judgment**		Low
**5**	**Bias in selection of the reported result**	
5.1	Were the data that produced this result analysed in accordance with a prespecified analysis plan that was finalised before unblinded outcome data were available for analysis?	PY
	Note: *The study was conducted and published in 2001, prior to the era of common clinical trial registry. While the primary outcomes (local recurrence, new recurrence, survival) are clearly stated in the methods section, suggesting a pre-defined analysis plan, the protocol was not publicly registered. The subgroup analysis for tumors ¡2 cm, while plausible as a pre-specified hypothesis, could also be exploratory. Therefore, the risk of potential selective reporting is considered low but cannot be fully excluded.*	
Is the numerical result being assessed likely to have been selected, on the basis of the results, from:		
5.2	Multiple eligible outcome measurements (eg, scales, definitions, time points) within the outcome domain?	N
	Note: *The primary outcomes (local recurrence, new recurrence, survival) were clearly defined prospectively in the methods section. There is no evidence of selection from multiple outcome measurements.*	
5.3	Multiple eligible analyses of the data?	N
	Note: *The authors used standard statistical methods (Kaplan-Meier, log-rank test) as stated in the methods. There is no indication that multiple analytical approaches were tried for the primary results.*	

Koda2001 *(continued)*

**Risk-of-bias judgment**	Low
**Overall bias**	Low

Lee2018

**Title:** A prospective randomized study comparing radiofrequency ablation and hepatic resection for hepatocellular carcinoma		
**Authors:** Hae Won Lee, Jeong Min Lee, Jung-Hwan Yoon, *et al.*		**Year:** 2018
**Interventions:** Radiofrequency ablation (RFA) vs. Surgical resection (SR)		
**Registration:** NCT02482909		
**1**	**Bias arising from the randomisation process**	
1.1	Was the allocation sequence random?<br>***Note:*** *The manuscript states, "Patients were recruited and assigned to 2 groups (the RFA and HR groups) by a stratified randomization method beforehand, which was developed by the Medical Research Collaborating Center (MRCC) of Seoul National University Hospital." This indicates the use of a proper random allocation sequence.*	Y
1.2	Was the allocation sequence concealed until participants were enrolled and assigned to interventions?<br>***Note:*** *The registry number was printed on envelopes in order, and the corresponding group name was inside each sealed envelope. Research nurses opened the envelopes in sequence after informed consent was obtained. This suggests adequate concealment, though the exact method of sequence generation is not fully detailed.*	PY
1.3	Did baseline differences between intervention groups suggest a problem with the randomisation process?<br>***Note:*** *Table 1 shows no significant differences in baseline characteristics (e.g., age, tumor size, Child-Pugh score, α-FP, platelet count) between the two groups, indicating that the randomization process was successful.*	N
**Risk-of-bias judgment**		Low
**2**	**Bias due to deviations from intended interventions**	
2.1	Were participants aware of their assigned intervention during the trial?<br>***Note:*** *Due to the nature of the interventions (surgery vs. ablation), participants were necessarily aware of their treatment assignment.*	Y
2.2	Were carers and people delivering the interventions aware of participants' assigned intervention during the trial?<br>***Note:*** *Surgeons and radiologists performing the procedures were aware of the treatment assignment.*	Y
2.3	If Y/PY/NI to 2.1 or 2.2: Were there deviations from the intended intervention that arose because of the trial context?<br>***Note:*** *Some patients switched treatments after randomization (e.g., 2 in the HR group requested RFA, 4 in the RFA group switched to HR). However, compared with the sample size, the influence may be minimal.*	PN
2.4	If Y/PY/NI to 2.3: Were these deviations likely to have affected the outcome?	NA
2.5	If Y/PY to 2.4: Were these deviations from intended intervention balanced between groups?	NA
2.6	Was an appropriate analysis used to estimate the effect of assignment to intervention?<br>***Note:*** *The primary analysis was intention-to-treat (ITT), and additional per-protocol (PP) and per-treatment (PT) analyses were also performed.*	Y
2.7	If N/PN/NI to 2.6: Was there potential for a substantial impact (on the result) of the failure to analyse participants in the group to which they were randomised?	NA

Lee2018 *(continued)*

**Risk-of-bias judgment**		Low
**3**	**Bias due to missing outcome data**	
3.1	Were data for this outcome available for all, or nearly all, participants randomised?<br>***Note:*** *Of the 68 randomised patients, 63 were included in the ITT analysis. Only 5 withdrew consent before treatment and were excluded. Follow-up data appear complete for the analysed patients.*	Y
3.2	If N/PN/NI to 3.1: Is there evidence that the result was not biased by missing outcome data?	NA
3.3	If N/PN to 3.2: Could missingness in the outcome depend on its true value?	NA
3.4	If Y/PY/NI to 3.3: Is it likely that missingness in the outcome depended on its true value?	NA
**Risk-of-bias judgment**		Low
**4**	**Bias in measurement of the outcome**	
4.1	Was the method of measuring the outcome inappropriate?<br>***Note:*** *Overall survival and disease-free survival are objective outcomes. Recurrence was assessed using imaging (CT/MRI) and classified into clear categories.*	N
4.2	Could measurement or ascertainment of the outcome have differed between intervention groups?<br>***Note:*** *The same follow-up protocol (imaging and laboratory tests) was applied to both groups.*	N
4.3	If N/PN/NI to 4.1 and 4.2: Were outcome assessors aware of the intervention received by study participants?<br>***Note:*** *Due to the nature of the interventions, assessors were likely aware of the treatment received.*	Y
4.4	If Y/PY/NI to 4.3: Could assessment of the outcome have been influenced by knowledge of intervention received?<br>***Note:*** *Although assessors were not blinded, the outcomes (survival, recurrence on imaging) are objective and unlikely to be influenced by knowledge of treatment.*	PN
4.5	If Y/PY/NI to 4.4: Is it likely that assessment of the outcome was influenced by knowledge of intervention received?	NA
**Risk-of-bias judgment**		Low
**5**	**Bias in selection of the reported result**	
5.1	Were the data that produced this result analysed in accordance with a prespecified analysis plan that was finalised before unblinded outcome data were available for analysis?<br>***Note:*** *The study was registered on ClinicalTrials.gov (NCT02482909), and the statistical methods (ITT, PP, PT analyses, log-rank, Breslow tests) align with typical RCT reporting.*	Y
Is the numerical result being assessed likely to have been selected, on the basis of the results, from:		
5.2	Multiple eligible outcome measurements (eg, scales, definitions, time points) within the outcome domain?<br>***Note:*** *The primary and secondary outcomes (overall survival, disease-free survival) are clearly defined and standard for such trials. No evidence of selective reporting of outcome measures.*	N
5.3	Multiple eligible analyses of the data?<br>***Note:*** *The analyses (ITT, PP, PT) were pre-specified and complementary. There is no indication of result-dependent selection from multiple analytic approaches.*	N
**Risk-of-bias judgment**		Low
**Overall bias**		Low

Lencioni2003

Title: Small hepatocellular carcinoma in cirrhosis: randomized comparison of radio-frequency thermal ablation versus percutaneous ethanol injection

Authors: Riccardo A. Lencioni, Hans-Peter Allgaier, Dania Cioni, *et al.*. **Year:** 2003

Interventions: Radiofrequency ablation (RFA) vs Percutaneous ethanol injection (PEI)

Registration number: NA

**1**	**Bias arising from the randomisation process**	
1.1	Was the allocation sequence random?<br>***Note:*** *The paper states that patients were assigned "by using a computer-generated randomization list." This is appropriate evidence that a random allocation sequence was generated.*	Y
1.2	Was the allocation sequence concealed until participants were enrolled and assigned to interventions?<br>***Note:*** *The article states that the randomization list was "not available to the treating physician." Under a relatively lenient RoB 2.0 reading, this is acceptable evidence that allocation was concealed from the clinicians enrolling/treating patients.*	PY
1.3	Did baseline differences between intervention groups suggest a problem with the randomisation process?<br>***Note:*** *The paper reports that there were no statistically significant differences in baseline characteristics between groups except for age and albumin concentration. Although those imbalances exist, they do not strongly suggest a failed randomization process, especially given the modest sample size and otherwise broadly comparable baseline profile.*	PN
**Risk-of-bias judgment**		Low
**2**	**Bias due to deviations from intended interventions**	
2.1	Were participants aware of their assigned intervention during the trial?<br>***Note:*** *Participants would almost certainly know whether they received RF ablation or PEI, because these procedures differ substantially in number of sessions, treatment experience, and technique. RF was usually completed in about one session, whereas PEI often required multiple sessions.*	PY
2.2	Were carers and people delivering the interventions aware of participants' assigned intervention during the trial?<br>***Note:*** *The treating physicians necessarily knew which procedure they were delivering, since RF ablation and PEI use different devices, procedures, and schedules.*	Y
2.3	If Y/PY/NI to 2.1 or 2.2: Were there deviations from the intended intervention that arose because of the trial context?<br>***Note:*** *There is no clear evidence of clinically important deviations caused by lack of blinding. Additional treatment for residual viable tissue was prespecified within each treatment strategy, and if repeated local treatment failed, further management was scheduled according to protocol. That looks more like planned management than problematic departure from assigned intervention.*	PN
2.4	If Y/PY/NI to 2.3: Were these deviations likely to have affected the outcome?	NA
2.5	If Y/PY to 2.4: Were these deviations from intended intervention balanced between groups?	NA
2.6	Was an appropriate analysis used to estimate the effect of assignment to intervention?	PY

	Note: *The paper analyzes patients according to the randomized RF and PEI groups and uses standard time-to-event methods for survival. Two patients assigned to RF were excluded shortly after randomization because they were retrospectively found to be ineligible. Strictly speaking, this is not a perfect modern ITT analysis, but under a lenient reading, these exclusions were based on post-randomization discovery of clear ineligibility rather than treatment-related switching or selective removal of poor outcomes.*	
2.7	If N/PN/NI to 2.6: Was there potential for a substantial impact (on the result) of the failure to analyse participants in the group to which they were randomised?	NA
**Risk-of-bias judgment**		Low
**3**	**Bias due to missing outcome data**	
3.1	Were data for this outcome available for all, or nearly all, participants randomised?	PY
	Note: *For the analyzed study population of 102 patients, the paper presents survival outcomes with Kaplan-Meier analysis and reports mean follow-up of about 22 months in both groups. The article does not emphasize major loss to follow-up as a study limitation, which suggests that survival data were available for nearly all analyzed participants.*	
3.2	If N/PN/NI to 3.1: Is there evidence that the result was not biased by missing outcome data?	NA
3.3	If N/PN to 3.2: Could missingness in the outcome depend on its true value?	NA
3.4	If Y/PY/NI to 3.3: Is it likely that missingness in the outcome depended on its true value?	NA
**Risk-of-bias judgment**		Low
**4**	**Bias in measurement of the outcome**	
4.1	Was the method of measuring the outcome inappropriate?	N
	Note: *The target result assessed here is overall survival, which is an appropriate and objective endpoint for a randomized trial of HCC treatment.*	
4.2	Could measurement or ascertainment of the outcome have differed between intervention groups?	PN
	Note: *Death is an objective outcome, and there is no clear reason to think that death ascertainment differed materially between the RF and PEI groups.*	
4.3	If N/PN/NI to 4.1 and 4.2: Were outcome assessors aware of the intervention received by study participants?	PY
	Note: *The study was not blinded, and the investigators were likely aware of which treatment each patient had received during follow-up.*	
4.4	If Y/PY/NI to 4.3: Could assessment of the outcome have been influenced by knowledge of intervention received?	PN
	Note: *Overall survival is a hard endpoint and is not realistically vulnerable to interpretive assessment bias.*	
4.5	If Y/PY/NI to 4.4: Is it likely that assessment of the outcome was influenced by knowledge of intervention received?	NA
**Risk-of-bias judgment**		Low
**5**	**Bias in selection of the reported result**	
5.1	Were the data that produced this result analysed in accordance with a prespecified analysis plan that was finalised before unblinded outcome data were available for analysis?	PY

	Note: *The study explicitly states its primary and secondary endpoints, gives a sample size rationale, and describes the main statistical methods in advance in the report. A separate protocol or SAP is not provided, so this cannot be confirmed to modern standards. But considering the publication year (when registration of clinical trial was not forcefully demanded), this is reasonably reassuring.*	
Is the numerical result being assessed likely to have been selected, on the basis of the results, from:		
5.2	Multiple eligible outcome measurements (eg, scales, definitions, time points) within the outcome domain?	N
	Note: *For the outcome domain of overall survival, the study is straightforward. The primary endpoint was clearly stated as overall survival rather than multiple interchangeable survival definitions.*	
5.3	Multiple eligible analyses of the data?	N
	Note: *The paper uses standard Kaplan-Meier and Cox regression methods for the main survival result. Although secondary analyses were performed for other outcomes, there is no strong sign that the main overall survival result was selectively chosen from many competing analyses.*	
**Risk-of-bias judgment**		Low
**Overall bias**		Low

Lin2005

Title: Randomised controlled trial comparing percutaneous radiofrequency thermal ablation, percutaneous ethanol injection, and percutaneous acetic acid injection to treat hepatocellular carcinoma of 3 cm or Less
Authors: S-M Lin, C-J Lin, C-C Lin, *et al.*. **Year:** 2003
Interventions: Radiofrequency ablation (RFA) vs Percutaneous ethanol injection (PEI)*
Registration number: NA

**1**	**Bias arising from the randomisation process**	
1.1	Was the allocation sequence random?<br>***Note:*** *The paper states that patients were randomized using a computer randomisation list, which is an appropriate random sequence generation method.*	Y
1.2	Was the allocation sequence concealed until participants were enrolled and assigned to interventions?<br>***Note:*** *The article states that the randomisation list was not available to the treating physician, which supports allocation concealment under a reasonably lenient RoB 2.0 interpretation.*	PY
1.3	Did baseline differences between intervention groups suggest a problem with the randomisation process?<br>***Note:*** *The baseline table and text state that there were no significant differences among the RFTA, PEI, and PAI groups for the listed clinical characteristics and follow-up duration. This does not suggest a problem with the randomization process.*	N
**Risk-of-bias judgment**		Low
**2**	**Bias due to deviations from intended interventions**	
2.1	Were participants aware of their assigned intervention during the trial?<br>***Note:*** *Participants would almost certainly know whether they received RF ablation or PEParticipants would almost certainly know whether they received radiofrequency thermal ablation, ethanol injection, or acetic acid injection, because these are materially different procedures with different treatment intensity and numbers of sessions.*	PY
2.2	Were carers and people delivering the interventions aware of participants' assigned intervention during the trial?<br>***Note:*** *The treating clinicians necessarily knew which intervention they were delivering because the three treatment techniques used different procedures, devices, and schedules.*	Y
2.3	If Y/PY/NI to 2.1 or 2.2: Were there deviations from the intended intervention that arose because of the trial context?<br>***Note:*** *The paper does not report important crossovers between groups or clear departures caused by knowledge of treatment assignment. Repeated treatment courses and booster treatment were part of the protocol, not obvious protocol violations. Patients with treatment failure later received additional or alternative therapies, but this appears to have happened after failure of the assigned local strategy rather than representing uncontrolled deviation during the comparison itself.*	PN
2.4	If Y/PY/NI to 2.3: Were these deviations likely to have affected the outcome?	NA
2.5	If Y/PY to 2.4: Were these deviations from intended intervention balanced between groups?	NA
2.6	Was an appropriate analysis used to estimate the effect of assignment to intervention?	PY

Lin2005 *(continued)*

	Note: *The article does not explicitly use the phrase intention-to-treat, so this is not as strong as a modern trial report. However, the survival analyses are presented at the randomized group level using Kaplan-Meier and Cox regression methods, and there is no obvious indication that patients were re-analysed according to a different actual-treatment grouping. Under a lenient interpretation, this is probably acceptable for estimating the effect of assignment.*	
2.7	If N/PN/NI to 2.6: Was there potential for a substantial impact (on the result) of the failure to analyse participants in the group to which they were randomised?	NA
**Risk-of-bias judgment**		Low
**3**	**Bias due to missing outcome data**	
3.1	Were data for this outcome available for all, or nearly all, participants randomised?	PY
	Note: *For overall survival, follow-up appears reasonably complete. The paper reports mean follow-up of about 26–28 months and specifically mentions only seven patients lost to follow-up in total across the three groups, with those losses occurring after recurrence. In a trial of 187 patients, this is not ideal but is still fairly limited.*	
3.2	If N/PN/NI to 3.1: Is there evidence that the result was not biased by missing outcome data?	NA
3.3	If N/PN to 3.2: Could missingness in the outcome depend on its true value?	NA
3.4	If Y/PY/NI to 3.3: Is it likely that missingness in the outcome depended on its true value?	NA
**Risk-of-bias judgment**		Low
**4**	**Bias in measurement of the outcome**	
4.1	Was the method of measuring the outcome inappropriate?	N
	Note: *The assessed outcome here is overall survival, which is an appropriate and objective end-point for a randomized comparison of HCC treatments.*	
4.2	Could measurement or ascertainment of the outcome have differed between intervention groups?	PN
	Note: *Death is an objective event, and there is no clear reason to think that death ascertainment differed materially among the three randomized groups.*	
4.3	If N/PN/NI to 4.1 and 4.2: Were outcome assessors aware of the intervention received by study participants?	PY
	Note: *The paper does not describe blinding, and in practice investigators were likely aware of treatment assignment during follow-up.*	
4.4	If Y/PY/NI to 4.3: Could assessment of the outcome have been influenced by knowledge of intervention received?	PN
	Note: *Because the outcome is overall survival, knowledge of the assigned intervention is very unlikely to influence whether death occurred.*	
4.5	If Y/PY/NI to 4.4: Is it likely that assessment of the outcome was influenced by knowledge of intervention received?	NA
**Risk-of-bias judgment**		Low
**5**	**Bias in selection of the reported result**	
5.1	Were the data that produced this result analysed in accordance with a prespecified analysis plan that was finalised before unblinded outcome data were available for analysis?	PY

Lin2005 *(continued)*

	Note: *The study explicitly states its primary and secondary endpoints, gives a sample size rationale, and describes the main statistical methods in advance in the report. A separate protocol or SAP is not provided, so this cannot be confirmed to modern standards. But considering the publication year (when registration of clinical trial was not forcefully demanded), this is reasonably reassuring.*	
Is the numerical result being assessed likely to have been selected, on the basis of the results, from:		
5.2	Multiple eligible outcome measurements (eg, scales, definitions, time points) within the outcome domain?	N
	Note: *For the domain of overall survival, the study appears straightforward. Although the paper also reports local recurrence and cancer-free survival, these are separate outcomes rather than multiple interchangeable versions of overall survival.*	
5.3	Multiple eligible analyses of the data?	N
	Note: *The main overall survival comparison was presented using standard Kaplan-Meier and Cox regression methods, and there is no strong indication that the authors selected this result from many competing alternative analyses.*	
**Risk-of-bias judgment**		Low
**Overall bias**		Low

* The PAI arm was excluded from the present analysis because percutaneous acetic acid injection (PAI) is no longer commonly used in contemporary HCC practice, and current evidence/guidelines do not support a clear substantive advantage of PAI over PEI. To improve clinical relevance, only the PEI and RFA arms were retained.

Liu2016

**Title:** Randomized clinical trial of chemoembolization plus radiofrequency ablation versus partial hepatectomy for hepatocellular carcinoma within the Milan criteria		
**Authors:** H. Liu, Z.-G. Wang, S.-Y. Fu, *et al.*.		**Year:** 2016
**Interventions:** Transcatheter arterial chemoembolization (TACE) + Radiofrequency ablation (RFA) vs Surgical resection (SR)		
**Registration number:** ACTRN12611000770965		
**1**	**Bias arising from the randomisation process**	
1.1	Was the allocation sequence random?<br>***Note:*** *The paper states that patients were randomized in a 1:1 ratio using random numbers, and that the allocation sequence was generated from a computer by a research assistant not involved in the study. This is adequate evidence of a genuinely random sequence.*	Y
1.2	Was the allocation sequence concealed until participants were enrolled and assigned to interventions?<br>***Note:*** *The report states that after consent was obtained, surgeons informed a research assistant, who then assigned participants according to the pre-generated allocation sequence. This suggests a reasonable degree of allocation concealment, although the paper does not describe more formal mechanisms such as central web randomization or sealed opaque envelopes. Under a relatively lenient interpretation, this is acceptable.*	PY
1.3	Did baseline differences between intervention groups suggest a problem with the randomisation process?<br>***Note:*** *Baseline variables were generally similar between groups, although AFP was significantly higher in the TACE + RFA group. A single imbalance does not necessarily imply failure of randomization, especially in a trial of 200 patients, but it does prevent an entirely perfect impression.*	PN
**Risk-of-bias judgment**		Low
**2**	**Bias due to deviations from intended interventions**	
2.1	Were participants aware of their assigned intervention during the trial?<br>***Note:*** *Participants would clearly know whether they underwent partial hepatectomy or TACE followed by RFA/TACE alone, because these are substantially different treatment strategies in invasiveness, timing, hospitalization, and clinical experience. The paper also explicitly says that double-blinding was impractical.*	Y
2.2	Were carers and people delivering the interventions aware of participants' assigned intervention during the trial?<br>***Note:*** *Clinicians necessarily knew which intervention they were delivering, because surgery and TACE/RFA are fundamentally different procedures.*	Y
2.3	If Y/PY/NI to 2.1 or 2.2: Were there deviations from the intended intervention that arose because of the trial context?<br>***Note:*** *Although 9 patients received TACE only rather than TACE + RFA and In addition, 4 patients later underwent salvage hepatectomy after RFA failure, the proportion is relatively low compared with the number of patients.*	PN
2.4	If Y/PY/NI to 2.3: Were these deviations likely to have affected the outcome?	NA
2.5	If Y/PY to 2.4: Were these deviations from intended intervention balanced between groups?	NA
2.6	Was an appropriate analysis used to estimate the effect of assignment to intervention?	Y

	Note: *The paper explicitly states that "An intention-to-treat analysis was followed when performing survival analysis." This is the correct approach for the ITT estimand, especially given the post-randomization deviations described above.*	
2.7	If N/PN/NI to 2.6: Was there potential for a substantial impact (on the result) of the failure to analyse participants in the group to which they were randomised?	NA
**Risk-of-bias judgment**		Low
**3**	**Bias due to missing outcome data**	
3.1	Were data for this outcome available for all, or nearly all, participants randomised?	PY
	Note: *Though some patients were lost to follow-up, the proportion was relatively low compared with the sample size. Thus, we suppose data for this outcome were available for nearly all participants.*	
3.2	If N/PN/NI to 3.1: Is there evidence that the result was not biased by missing outcome data?	NA
3.3	If N/PN to 3.2: Could missingness in the outcome depend on its true value?	NA
3.4	If Y/PY/NI to 3.3: Is it likely that missingness in the outcome depended on its true value?	NA
**Risk-of-bias judgment**		Low
**4**	**Bias in measurement of the outcome**	
4.1	Was the method of measuring the outcome inappropriate?	N
	Note: *The target outcome here is overall survival, which is an appropriate and highly objective endpoint for this comparison.*	
4.2	Could measurement or ascertainment of the outcome have differed between intervention groups?	PN
	Note: *Death is an objective outcome, and there is no obvious reason to think that ascertainment of death differed materially between the two groups.*	
4.3	If N/PN/NI to 4.1 and 4.2: Were outcome assessors aware of the intervention received by study participants?	PY
	Note: *The study was not blinded, and investigators were very likely aware of the assigned intervention during follow-up.*	
4.4	If Y/PY/NI to 4.3: Could assessment of the outcome have been influenced by knowledge of intervention received?	PN
	Note: *Because the outcome assessed here is overall survival, awareness of treatment assignment is very unlikely to affect whether death occurred.*	
4.5	If Y/PY/NI to 4.4: Is it likely that assessment of the outcome was influenced by knowledge of intervention received?	NA
**Risk-of-bias judgment**		Low
**5**	**Bias in selection of the reported result**	
5.1	Were the data that produced this result analysed in accordance with a prespecified analysis plan that was finalised before unblinded outcome data were available for analysis?	PY
	Note: *This study was registered, and the implementation and analysis were in line with the registration.*	
Is the numerical result being assessed likely to have been selected, on the basis of the results, from:		
5.2	Multiple eligible outcome measurements (eg, scales, definitions, time points) within the outcome domain?	N

Liu2016 *(continued)*

	Note: *For the outcome domain of overall survival, the study is relatively straightforward. OS was explicitly identified as the primary outcome rather than one of many interchangeable survival definitions.*	
5.3	Multiple eligible analyses of the data?	N
	Note: *The paper reports both Kaplan–Meier/log-rank analysis and Gray's test for competing risk analysis, and the conclusions differ slightly between endpoints depending on the analytical approach, especially for RFS. This does not imply misconduct, but it does indicate some analytical multiplicity. In addition, subgroup analyses by tumour size were reported.*	
**Risk-of-bias judgment**		Low
**Overall bias**		Low

Mizuki2010

Title: Addition of transcatheter arterial chemoembolization decreased local recurrence but had no survival benefit to percutaneous ethanol injection therapy for patients with small hepatocellular carcinoma: A multicenter randomized control study

Authors: Akira Mizuki, Masayuki Tatemichi, Nobuhiro Tsukada, *et al.*. **Year:** 2010

Interventions: Transcatheter arterial chemoembolization (TACE) vs Percutaneous ethanol injection (PEI)

Registration number: NA

**1**	**Bias arising from the randomisation process**	
1.1	Was the allocation sequence random?<br>***Note:*** *The paper states that this was a randomized controlled study and that patients were randomized by a sealed-envelope method.*	Y
1.2	Was the allocation sequence concealed until participants were enrolled and assigned to interventions?<br>***Note:*** *The paper explicitly states that randomization was performed using a sealed-envelope method, which usually supports allocation concealment if implemented properly.*	PY
1.3	Did baseline differences between intervention groups suggest a problem with the randomisation process?<br>***Note:*** *The authors state that no significant differences were found between the two groups in baseline characteristics, and the table on page 3 does not show any clearly extreme imbalance in age, sex, liver disease etiology, liver function variables, tumor number, or tumor size. This does not suggest a problem with the randomization process.*	N
**Risk-of-bias judgment**		Low
**2**	**Bias due to deviations from intended interventions**	
2.1	Were participants aware of their assigned intervention during the trial?<br>***Note:*** *Participants would almost certainly know whether they received PEI alone or TACE followed by PEI, because these are materially different procedures with different treatment intensity and procedural experience.*	PY
2.2	Were carers and people delivering the interventions aware of participants' assigned intervention during the trial?<br>***Note:*** *Clinicians necessarily knew which intervention they were delivering because PEI alone and TACE-PEI required different procedures, equipment, and treatment steps.*	Y
2.3	If Y/PY/NI to 2.1 or 2.2: Were there deviations from the intended intervention that arose because of the trial context?<br>***Note:*** *The paper does not report important crossover between the two randomized strategies or obvious protocol-breaking deviations caused by lack of blinding. Re-treatment of recurrent tumors was part of the follow-up management plan and appears to have been prespecified, rather than being uncontrolled deviation from the assigned intervention.*	PN
2.4	If Y/PY/NI to 2.3: Were these deviations likely to have affected the outcome?	NA
2.5	If Y/PY to 2.4: Were these deviations from intended intervention balanced between groups?	NA
2.6	Was an appropriate analysis used to estimate the effect of assignment to intervention?<br>***Note:*** *Though some patients quitted this study, they were excluded before the start of the treatment. So removing them in the subsequent analysis was in accordance to ITT principle.*	PY
2.7	If N/PN/NI to 2.6: Was there potential for a substantial impact (on the result) of the failure to analyse participants in the group to which they were randomised?	NA

Mizuki2010 *(continued)*

**Risk-of-bias judgment**		Low
**3**	**Bias due to missing outcome data**	
3.1	Were data for this outcome available for all, or nearly all, participants randomised?<br>***Note:*** *The number of patients who quitted was relatively low (< 20%), so we deem the outcome was available for nearly all participants.*	PY
3.2	If N/PN/NI to 3.1: Is there evidence that the result was not biased by missing outcome data?	NA
3.3	If N/PN to 3.2: Could missingness in the outcome depend on its true value?	NA
3.4	If Y/PY/NI to 3.3: Is it likely that missingness in the outcome depended on its true value?	NA
**Risk-of-bias judgment**		Low
**4**	**Bias in measurement of the outcome**	
4.1	Was the method of measuring the outcome inappropriate?<br>***Note:*** *The target result assessed here is overall survival / patient death, which is an appropriate and objective endpoint for a randomized comparison of HCC treatment strategies.*	N
4.2	Could measurement or ascertainment of the outcome have differed between intervention groups?<br>***Note:*** *Death is an objective event, and there is no clear reason to think that death ascertainment differed materially between the TACE-PEI and PEI-alone groups.*	PN
4.3	If N/PN/NI to 4.1 and 4.2: Were outcome assessors aware of the intervention received by study participants?<br>***Note:*** *The paper does not describe blinding, and in practice the investigators were likely aware of treatment assignment during follow-up.*	PY
4.4	If Y/PY/NI to 4.3: Could assessment of the outcome have been influenced by knowledge of intervention received?<br>***Note:*** *Because the outcome is overall survival, knowledge of the assigned intervention is very unlikely to influence whether death occurred.*	PN
4.5	If Y/PY/NI to 4.4: Is it likely that assessment of the outcome was influenced by knowledge of intervention received?	NA
**Risk-of-bias judgment**		Low
**5**	**Bias in selection of the reported result**	
5.1	Were the data that produced this result analysed in accordance with a prespecified analysis plan that was finalised before unblinded outcome data were available for analysis?<br>***Note:*** *Patients were enrolled before 2000 (rom 1997 to 1999), when the registration platform was not widely applied. Concerning this was a multicenter study and the protocol was approved by the review broad of each hospital, we suppose the data produced were in accordance with a prespecified analysis plan.*	PY
Is the numerical result being assessed likely to have been selected, on the basis of the results, from:		
5.2	Multiple eligible outcome measurements (eg, scales, definitions, time points) within the outcome domain?<br>***Note:*** *For the domain of overall survival, the outcome is straightforward. The study also reports cancer-free time and local recurrence pattern, but these are different outcomes rather than multiple interchangeable measures of survival.*	N
5.3	Multiple eligible analyses of the data?	N

**Mizuki2010** *(continued)*	
Note: *The main survival comparison was presented using standard Kaplan-Meier methods, and there is no strong sign that the authors selected this result from many competing alternative analyses.*	
**Risk-of-bias judgment**	Low
**Overall bias**	Low

Ng2017

**Title:** Randomized clinical trial of hepatic resection versus radiofrequency ablation for early-stage hepatocellular carcinoma		
**Authors:** K. K. C. Ng, K. S. H. Chok, A. C. Y. Chan, *et al.*		**Year:** 2017
**Interventions:** Radiofrequency ablation (RFA) vs. Surgical resection (SR)		
**Registration:** HKUCTR-10		
**1**	**Bias arising from the randomisation process**	
1.1	Was the allocation sequence random? *Note: The manuscript states, "Randomization (1 : 1 ratio) was performed after informed consent had been obtained using sealed consecutively numbered envelopes." This describes the use of a random allocation sequence.*	Y
1.2	Was the allocation sequence concealed until participants were enrolled and assigned to interventions? *Note: The envelopes were "kept by a research assistant not involved in the treatment of the patient, and were opened only when the patient was considered suitable for both hepatic resection and RFA after full preoperative investigations." This describes adequate allocation concealment.*	PY
1.3	Did baseline differences between intervention groups suggest a problem with the randomisation process? *Note: Table 1 shows no significant differences in baseline characteristics.*	N
**Risk-of-bias judgment**		Low
**2**	**Bias due to deviations from intended interventions**	
2.1	Were participants aware of their assigned intervention during the trial? *Note: Due to the nature of the interventions (surgery vs. ablation), participants were necessarily aware of their treatment assignment.*	Y
2.2	Were carers and people delivering the interventions aware of participants' assigned intervention during the trial? *Note: Surgeons and radiologists performing the procedures were aware of the treatment assignment.*	Y
2.3	If Y/PY/NI to 2.1 or 2.2: Were there deviations from the intended intervention that arose because of the trial context? *Note: Though 2 patients in the resection group received RFA or combined treatment, and 2 patients in the RFA group underwent resection, we supposed no significant deviation existed compared with the relatively large sample size.*	PN
2.4	If Y/PY/NI to 2.3: Were these deviations likely to have affected the outcome?	NA
2.5	If Y/PY to 2.4: Were these deviations from intended intervention balanced between groups?	NA
2.6	Was an appropriate analysis used to estimate the effect of assignment to intervention? *Note: The primary analysis was performed on an intention-to-treat (ITT) basis, which is appropriate for RCTs.*	Y
2.7	If N/PN/NI to 2.6: Was there potential for a substantial impact (on the result) of the failure to analyse participants in the group to which they were randomised?	NA
**Risk-of-bias judgment**		Low
**3**	**Bias due to missing outcome data**	
3.1	Were data for this outcome available for all, or nearly all, participants randomised? *Note: All 218 randomised patients were included in the ITT analysis for the primary outcome (tumor recurrence). There were no losses to follow-up (Fig. 1).*	Y

**Ng2017** *(continued)*		
3.2	If N/PN/NI to 3.1: Is there evidence that the result was not biased by missing outcome data?	NA
3.3	If N/PN to 3.2: Could missingness in the outcome depend on its true value?	NA
3.4	If Y/PY/NI to 3.3: Is it likely that missingness in the outcome depended on its true value?	NA
**Risk-of-bias judgment**		Low
**4**	**Bias in measurement of the outcome**	
4.1	Was the method of measuring the outcome inappropriate?	N
	Note: *Overall survival, disease-free survival, and tumor recurrence are objective outcomes. Recurrence was assessed using standardized imaging (CT) at regular intervals.*	
4.2	Could measurement or ascertainment of the outcome have differed between intervention groups?	N
	Note: *The same follow-up protocol (imaging and laboratory tests) was applied uniformly to both groups.*	
4.3	If N/PN/NI to 4.1 and 4.2: Were outcome assessors aware of the intervention received by study participants?	Y
	Note: *Due to the nature of the interventions, assessors were likely aware of the treatment received.*	
4.4	If Y/PY/NI to 4.3: Could assessment of the outcome have been influenced by knowledge of intervention received?	PN
	Note: *Although assessors were not blinded, the primary outcomes (survival, radiologically confirmed recurrence) are objective and unlikely to be influenced by knowledge of the intervention.*	
4.5	If Y/PY/NI to 4.4: Is it likely that assessment of the outcome was influenced by knowledge of intervention received?	NA
**Risk-of-bias judgment**		Low
**5**	**Bias in selection of the reported result**	
5.1	Were the data that produced this result analysed in accordance with a prespecified analysis plan that was finalised before unblinded outcome data were available for analysis?	Y
	Note: *The study was registered (HKUCTR-10). The primary and secondary outcomes specified in the manuscript (overall tumor recurrence, overall survival, disease-free survival) align with typical RCT reporting. However, the registry entry details are not provided to confirm full adherence.*	
Is the numerical result being assessed likely to have been selected, on the basis of the results, from:		
5.2	Multiple eligible outcome measurements (eg, scales, definitions, time points) within the outcome domain?	N
	Note: *The outcomes are clearly defined and standard for such trials. Subgroup analyses (e.g., very early HCC) were pre-specified. No evidence of selective reporting of outcome measures.*	
5.3	Multiple eligible analyses of the data?	N
	Note: *The primary analysis (ITT) was pre-specified. Additional multivariable and subgroup analyses are complementary and justified. No indication of result-dependent selection from multiple analytic approaches.*	
**Risk-of-bias judgment**		Low
**Overall bias**		Low

Orlacchio2014

**Title:** Comparative evaluation of percutaneous laser and radiofrequency ablation in patients with HCC smaller than 4 cm		
**Authors:** Antonio Orlacchio, Francesca Bolacchi, Fabrizio Chegai, *et al.*.		**Year:** 2010
**Interventions:** Radiofrequency ablation (RFA) vs Laser ablation (LA)		
**Registration number:** NA		
**1**	**Bias arising from the randomisation process**	
1.1	Was the allocation sequence random?	Y
	Note: *The paper explicitly states that "Randomisation software was used to allocate each patient to a treatment group." This is acceptable evidence that a random allocation sequence was generated.*	
1.2	Was the allocation sequence concealed until participants were enrolled and assigned to interventions?	PY
	Note: *The paper says that randomisation software was used, so concealment could be confirmed from the report.*	
1.3	Did baseline differences between intervention groups suggest a problem with the randomisation process?	N
	Note: *The baseline table and results section indicate that the two groups were homogeneous in terms of baseline characteristics and nodule dimensions, with no obviously major imbalances in age, sex, Child-Pugh class, lesion size, or tumour distribution. Under a lenient interpretation, this does not suggest a problem with randomization.*	
**Risk-of-bias judgment**		Low
**2**	**Bias due to deviations from intended interventions**	
2.1	Were participants aware of their assigned intervention during the trial?	PY
	Note: *Participants would probably know whether they received PLA or RFA, because these are different procedures using different devices and treatment experiences, even though both were performed percutaneously under image guidance.*	
2.2	Were carers and people delivering the interventions aware of participants' assigned intervention during the trial?	Y
	Note: *The treating radiologist necessarily knew which intervention was being delivered because PLA and RFA used different equipment, needles, and technical protocols. The paper also states that all procedures were performed by a single radiologist.*	
2.3	If Y/PY/NI to 2.1 or 2.2: Were there deviations from the intended intervention that arose because of the trial context?	PN
	Note: *There is no clear evidence of important crossovers or protocol-breaking deviations caused by the lack of blinding. Patients with partial response at 30 days were re-treated with the same technique, which appears to have been part of the study treatment strategy rather than a deviation from it.*	
2.4	If Y/PY/NI to 2.3: Were these deviations likely to have affected the outcome?	NA
2.5	If Y/PY to 2.4: Were these deviations from intended intervention balanced between groups?	NA
2.6	Was an appropriate analysis used to estimate the effect of assignment to intervention?	PY
	Note: *The paper reports 30 randomized patients, 15 in each group, and gives the 30-day CR results for all lesions/patients after completion of the planned treatment course, including second treatment where needed. It does not explicitly state that an ITT analysis was performed, but for this short-term efficacy result the analysis appears close to the randomized-group comparison. Thus, this is probably acceptable.*	

Orlacchio2014 *(continued)*

2.7	If N/PN/NI to 2.6: Was there potential for a substantial impact (on the result) of the failure to analyse participants in the group to which they were randomised?	NA
**Risk-of-bias judgment**		Low
**3**	**Bias due to missing outcome data**	
3.1	Were data for this outcome available for all, or nearly all, participants randomised?<br>***Note:*** *For the 30-day complete response result, the paper reports outcomes for all 30 treated HCC nodules / 30 patients. Five PLA patients and two RFA patients had partial response after the first procedure and were re-treated, and final CR status after the second procedure is reported. This suggests outcome data were available for essentially all randomized participants for this endpoint.*	PY
3.2	If N/PN/NI to 3.1: Is there evidence that the result was not biased by missing outcome data?	NA
3.3	If N/PN to 3.2: Could missingness in the outcome depend on its true value?	NA
3.4	If Y/PY/NI to 3.3: Is it likely that missingness in the outcome depended on its true value?	NA
**Risk-of-bias judgment**		Low
**4**	**Bias in measurement of the outcome**	
4.1	Was the method of measuring the outcome inappropriate?<br>***Note:*** *The paper assessed treatment efficacy using dynamic triphasic CT at 30 days, and response was evaluated according to mRECIST criteria. Complete response was defined as disappearance of all signs of lesion and no pathological enhancement at the treated edges. This is an appropriate method for assessing local ablation response in HCC.*	N
4.2	Could measurement or ascertainment of the outcome have differed between intervention groups?<br>***Note:*** *The same CT-based response framework and the same 30-day assessment schedule were applied to both PLA and RFA groups. There is no indication that one group was measured using a different standard.*	PN
4.3	If N/PN/NI to 4.1 and 4.2: Were outcome assessors aware of the intervention received by study participants?<br>***Note:*** *The paper does not describe blinding, and in practice the investigators were likely aware of treatment assignment during follow-up.*	PY
4.4	If Y/PY/NI to 4.3: Could assessment of the outcome have been influenced by knowledge of intervention received?<br>***Note:*** *Because the outcomes were objective, knowledge of the assigned intervention is very unlikely to influence whether death occurred.*	PN
4.5	If Y/PY/NI to 4.4: Is it likely that assessment of the outcome was influenced by knowledge of intervention received?	NA
**Risk-of-bias judgment**		Low
**5**	**Bias in selection of the reported result**	
5.1	Were the data that produced this result analysed in accordance with a prespecified analysis plan that was finalised before unblinded outcome data were available for analysis?<br>***Note:*** *No prespecified registration record was available.*	NI
Is the numerical result being assessed likely to have been selected, on the basis of the results, from:		
5.2	Multiple eligible outcome measurements (eg, scales, definitions, time points) within the outcome domain?	N

Orlacchio2014 *(continued)*

	Note: *For this outcome domain, the study is reasonably specific: complete response at 30 days assessed by CT using mRECIST. Although the paper also reports local recurrence-free survival and complications, these are different outcomes rather than multiple interchangeable measures of the same 30-day CR result.*	
5.3	Multiple eligible analyses of the data?	PN
	Note: *The paper uses straightforward comparative statistics and does not show obvious evidence that the 30-day CR result was selectively chosen from many competing analyses. Some subgroup exploration by tumour size was performed, but the main CR result itself appears directly reported.*	
**Risk-of-bias judgment**		Some concerns
**Overall bias**		Some concerns

Park2021

**Title:** Switching Monopolar No-Touch Radiofrequency Ablation Using Octopus Electrodes for Small Hepatocellular Carcinoma: A Randomized Clinical Trial		
**Authors:** Saejin Park, Eunju Cho, Jeonghoon Lee, *et al.*		**Year:** 2021
**Interventions:** Radiofrequency ablation (RFA) vs. No-touch RFA (NT-RFA)		
**Registration:** NCT03375281		
**1**	**Bias arising from the randomisation process**	
1.1	Was the allocation sequence random?<br>***Note:*** *The manuscript states, "participants... were randomly assigned to two groups... by the stratified randomization and block randomization method beforehand, which was developed by the Medical Research Collaborating Center of our institution." This describes a proper random allocation sequence.*	Y
1.2	Was the allocation sequence concealed until participants were enrolled and assigned to interventions?<br>***Note:*** *The use of a centralized method developed by the Medical Research Collaborating Center implies that the allocation sequence was concealed from the investigators enrolling participants until after assignment.*	Y
1.3	Did baseline differences between intervention groups suggest a problem with the randomisation process?<br>***Note:*** *Table 1 shows no significant differences in baseline characteristics (e.g., age, sex, etiology, tumor size, location, previous treatment) between the two groups, indicating successful randomization.*	N
**Risk-of-bias judgment**		Low
**2**	**Bias due to deviations from intended interventions**	
2.1	Were participants aware of their assigned intervention during the trial?<br>***Note:*** *Due to the nature of the percutaneous interventions, participants were likely aware of the treatment assignment, though the specific technical differences might not have been apparent.*	Y
2.2	Were carers and people delivering the interventions aware of participants' assigned intervention during the trial?<br>***Note:*** *The radiologists performing the procedures were necessarily aware of the assigned technique (no-touch vs. conventional).*	Y
2.3	If Y/PY/NI to 2.1 or 2.2: Were there deviations from the intended intervention that arose because of the trial context?<br>***Note:*** *The CONSORT diagram (Fig. 1) indicates that all participants received the intervention to which they were randomized. There were no crossovers or major protocol deviations reported.*	N
2.4	If Y/PY/NI to 2.3: Were these deviations likely to have affected the outcome?	NA
2.5	If Y/PY to 2.4: Were these deviations from intended intervention balanced between groups?	NA
2.6	Was an appropriate analysis used to estimate the effect of assignment to intervention?<br>***Note:*** *The primary analysis was performed on an intention-to-treat (ITT) basis, which is appropriate for RCTs.*	Y
2.7	If N/PN/NI to 2.6: Was there potential for a substantial impact (on the result) of the failure to analyse participants in the group to which they were randomised?	NA
**Risk-of-bias judgment**		Low
**3**	**Bias due to missing outcome data**	

Park2021 *(continued)*

3.1	Were data for this outcome available for all, or nearly all, participants randomised?<br>***Note:*** *Of the 116 randomized participants, 5 were lost to follow-up (3 in conventional, 2 in no-touch group), representing a low attrition rate (4.3%). The primary outcome (LTP) was likely available for the vast majority, and the analysis appears complete.*	Y
3.2	If N/PN/NI to 3.1: Is there evidence that the result was not biased by missing outcome data?	NA
3.3	If N/PN to 3.2: Could missingness in the outcome depend on its true value?	NA
3.4	If Y/PY/NI to 3.3: Is it likely that missingness in the outcome depended on its true value?	NA
**Risk-of-bias judgment**		Low
**4**	**Bias in measurement of the outcome**	
4.1	Was the method of measuring the outcome inappropriate?<br>***Note:*** *Local Tumor Progression (LTP) is an objective outcome, assessed by imaging (CT) according to standard radiological criteria.*	N
4.2	Could measurement or ascertainment of the outcome have differed between intervention groups?<br>***Note:*** *The same follow-up imaging protocol was applied uniformly to both groups.*	N
4.3	If N/PN/NI to 4.1 and 4.2: Were outcome assessors aware of the intervention received by study participants?<br>***Note:*** *The radiologists assessing the follow-up scans were likely aware of the treatment group, as the ablation zone appearance might differ between techniques.*	Y
4.4	If Y/PY/NI to 4.3: Could assessment of the outcome have been influenced by knowledge of intervention received?<br>***Note:*** *Although the outcome assessors were likely not blinded, the primary outcome (LTP on CT) is a reasonably objective radiological finding, making significant bias less likely.*	PN
4.5	If Y/PY/NI to 4.4: Is it likely that assessment of the outcome was influenced by knowledge of intervention received?	NA
**Risk-of-bias judgment**		Low
**5**	**Bias in selection of the reported result**	
5.1	Were the data that produced this result analysed in accordance with a prespecified analysis plan that was finalised before unblinded outcome data were available for analysis?<br>***Note:*** *The study was registered on ClinicalTrials.gov (NCT03375281). The primary and secondary outcomes specified in the manuscript align with the registry record, and the statistical methods (Kaplan-Meier, Cox regression) are standard for this type of trial.*	Y
Is the numerical result being assessed likely to have been selected, on the basis of the results, from:		
5.2	Multiple eligible outcome measurements (eg, scales, definitions, time points) within the outcome domain?<br>***Note:*** *The primary outcome (cumulative incidence of LTP) is clearly defined. No evidence suggests selective reporting from multiple measurements.*	N
5.3	Multiple eligible analyses of the data?<br>***Note:*** *The primary analysis (Kaplan-Meier with log-rank test) and secondary analyses (Cox regression) are standard and pre-specified. No indication of result-dependent selection from multiple analytic approaches.*	N
**Risk-of-bias judgment**		Low
**Overall bias**		Low

Shibata2002

**Title:** Small Hepatocellular Carcinoma: Comparison of Radio-frequency Ablation and Percutaneous Microwave Coagulation Therapy	
**Authors:** Toshiya Shibata, Yuji Iimuro, Yuzo Yamamoto, *et al.*	**Year:** 2002
**Interventions:** Radiofrequency ablation (RFA) vs. Microwave ablation (MWA)	
**Registration:** NCT03375281	

**1**	**Bias arising from the randomisation process**	
1.1	Was the allocation sequence random?<br>***Note:*** *The patients were randomly assigned into two groups using sealed envelopes, and they were told that the two therapies were equal in effectiveness. Thus, the allocation process was random.*	Y
1.2	Was the allocation sequence concealed until participants were enrolled and assigned to interventions?<br>***Note:*** *he paper states that patients were assigned "with use of sealed envelopes" to the RF ablation or PMC group. Although the details are not as complete as in a modern trial report, this is reasonably supportive of allocation concealment under a relatively forgiving RoB 2.0 interpretation.*	PY
1.3	Did baseline differences between intervention groups suggest a problem with the randomisation process?<br>***Note:*** *The baseline table and results section indicate no significant difference between groups for age, lesion size, sex, Child-Pugh class, number of lesions, viral markers, or AFP status. This does not suggest a problem with the randomization process.*	N
**Risk-of-bias judgment**		Low
**2**	**Bias due to deviations from intended interventions**	
2.1	Were participants aware of their assigned intervention during the trial?<br>***Note:*** *Participants would almost certainly know whether they received RF ablation or PMC, because these were different procedures with different devices, numbers of sessions, and treatment experiences. The paper reports a mean of 1.1 sessions per nodule in the RF group versus 2.4 sessions per nodule in the PMC group.*	Y
2.2	Were carers and people delivering the interventions aware of participants' assigned intervention during the trial?<br>***Note:*** *The treating physician necessarily knew which intervention was being delivered, because RF ablation and PMC used different equipment and technical protocols. In fact, the paper states that one author performed both RF ablation and PMC procedures.*	Y
2.3	If Y/PY/NI to 2.1 or 2.2: Were there deviations from the intended intervention that arose because of the trial context?<br>***Note:*** *There is no clear evidence of important crossover or protocol-breaking deviations caused by lack of blinding. When incomplete necrosis was seen at 1 week, additional RF ablation or PMC was performed using the same assigned technique, which appears to have been part of the planned treatment strategy rather than a deviation from it.*	N
2.4	If Y/PY/NI to 2.3: Were these deviations likely to have affected the outcome?	NA
2.5	If Y/PY to 2.4: Were these deviations from intended intervention balanced between groups?	NA
2.6	Was an appropriate analysis used to estimate the effect of assignment to intervention?<br>***Note:*** *The analysis was performed on an intention-to-treat (ITT) basis, which is appropriate.*	Y
2.7	If N/PN/NI to 2.6: Was there potential for a substantial impact (on the result) of the failure to analyse participants in the group to which they were randomised?	NA

Shibata2002 *(continued)*

**Risk-of-bias judgment**		Low
**3**	**Bias due to missing outcome data**	
3.1	Were data for this outcome available for all, or nearly all, participants randomised? ***Note:*** *For the 1-month complete therapeutic effect outcome, the paper appears to report results for all randomized nodules/patients after completion of treatment sessions within 1 month. There is no indication of meaningful early dropout before this endpoint was assessed.*	Y
3.2	If N/PN/NI to 3.1: Is there evidence that the result was not biased by missing outcome data?	NA
3.3	If N/PN to 3.2: Could missingness in the outcome depend on its true value?	NA
3.4	If Y/PY/NI to 3.3: Is it likely that missingness in the outcome depended on its true value?	NA
**Risk-of-bias judgment**		Low
**4**	**Bias in measurement of the outcome**	
4.1	Was the method of measuring the outcome inappropriate? ***Note:*** *The study assessed therapeutic effect using dynamic CT at 1 week and 1 month after the initial treatments. Complete therapeutic effect was defined by absence of enhancement and adequate nonenhancing area at the treated site. This is an appropriate method for evaluating local ablation efficacy in HCC.*	N
4.2	Could measurement or ascertainment of the outcome have differed between intervention groups? ***Note:*** *The same CT-based assessment framework and time points were applied to both the RF ablation and PMC groups. There is no indication that one group was evaluated by a different standard.*	PN
4.3	If N/PN/NI to 4.1 and 4.2: Were outcome assessors aware of the intervention received by study participants? ***Note:*** *The paper states that the CT scans were interpreted by one author, but it does not say that this assessment was blinded. Therefore, it is likely that the assessor knew which treatment had been used.*	Y
4.4	If Y/PY/NI to 4.3: Could assessment of the outcome have been influenced by knowledge of intervention received? ***Note:*** *Although the outcome assessor was likely not blinded, the primary outcome (CT) is a reasonably objective radiological finding, making significant bias less likely.*	PN
4.5	If Y/PY/NI to 4.4: Is it likely that assessment of the outcome was influenced by knowledge of intervention received?	NA
**Risk-of-bias judgment**		Low
**5**	**Bias in selection of the reported result**	
5.1	Were the data that produced this result analysed in accordance with a prespecified analysis plan that was finalised before unblinded outcome data were available for analysis? ***Note:*** *This study was not registered. But considering its time when online registration was not popular and necessary, this couldn't be the main reason for denying its robustness in methodology. Additionally, we could see that the enrollment of patients was described in detail, that the analytical process was arranged logically, that the results parts followed the methods parts closely, and that true CT images were provided. Thus, we suppose the data analysis was in accordance with a prespecified analysis plan.*	PY
Is the numerical result being assessed likely to have been selected, on the basis of the results, from:		

Shibata2002 *(continued)*

5.2	Multiple eligible outcome measurements (eg, scales, definitions, time points) within the outcome domain?<br>***Note:*** *For this outcome domain, the paper is reasonably specific: complete therapeutic effect at 1 month after the initial treatment course. The study also reports complication rates and residual foci of untreated disease during follow-up, but these are separate outcomes rather than interchangeable versions of the same short-term efficacy endpoint.*	N
5.3	Multiple eligible analyses of the data?<br>***Note:*** *Though the study involved results at the nodule level, we only included outcomes at the treatment level. In this way, no multiple eligible analyses of the data were included.*	N
**Risk-of-bias judgment**		Low
**Overall bias**		Low

Shibata2009

**Title:** Small hepatocellular carcinoma: is radiofrequency ablation combined with transcatheter arterial chemoembolization more effective than radiofrequency ablation alone for treatment?	
**Authors:** Saejin Park, Eunju Cho, Jeonghoon Lee, *et al.*	**Year:** 2009
**Interventions:** Radiofrequency ablation (RFA) + Transcatheter arterial chemoembolization (TACE) vs. RFA	
**Registration:** NCT03375281	

**1**	**Bias arising from the randomisation process**	
1.1	Was the allocation sequence random?<br>***Note:*** *The manuscript states that patients were "randomly assigned" but the method is described as: "The patients scheduled to undergo the procedure on Tuesday underwent TACE first... The patients scheduled for Thursday underwent radiofrequency ablation alone." This method of allocation based on the day of the week is not a truly random process and introduces a high risk of bias.*	N
1.2	Was the allocation sequence concealed until participants were enrolled and assigned to interventions?<br>***Note:*** *The allocation was predictable based on the schedule (Tuesday vs. Thursday). There is no mention of concealment, and the nature of the allocation method means it could not be concealed.*	N
1.3	Did baseline differences between intervention groups suggest a problem with the randomisation process?<br>***Note:*** *Table 1 shows no significant differences in baseline characteristics between the two groups. However, given the highly flawed allocation method, the lack of differences is likely due to chance rather than successful randomization.*	N
**Risk-of-bias judgment**		High
**2**	**Bias due to deviations from intended interventions**	
2.1	Were participants aware of their assigned intervention during the trial?<br>***Note:*** *Due to the nature of the interventions, participants were necessarily aware of their treatment assignment (whether they received TACE or not).*	Y
2.2	Were carers and people delivering the interventions aware of participants' assigned intervention during the trial?<br>***Note:*** *The interventional radiologists performing the procedures were aware of the treatment assignment.*	Y
2.3	If Y/PY/NI to 2.1 or 2.2: Were there deviations from the intended intervention that arose because of the trial context?<br>***Note:*** *The manuscript states that all patients received the allocated intervention. There were no reported crossovers or major protocol deviations.*	N
2.4	If Y/PY/NI to 2.3: Were these deviations likely to have affected the outcome?	NA
2.5	If Y/PY to 2.4: Were these deviations from intended intervention balanced between groups?	NA
2.6	Was an appropriate analysis used to estimate the effect of assignment to intervention?<br>***Note:*** *The analysis was performed on an intention-to-treat (ITT) basis, which is appropriate.*	Y
2.7	If N/PN/NI to 2.6: Was there potential for a substantial impact (on the result) of the failure to analyse participants in the group to which they were randomised?	NA
**Risk-of-bias judgment**		Low
**3**	**Bias due to missing outcome data**	
3.1	Were data for this outcome available for all, or nearly all, participants randomised?	Y

	Note: *All 89 randomised patients were included in the analysis. There is no mention of loss to follow-up for the primary outcome (local tumor progression).*	
3.2	If N/PN/NI to 3.1: Is there evidence that the result was not biased by missing outcome data?	NA
3.3	If N/PN to 3.2: Could missingness in the outcome depend on its true value?	NA
3.4	If Y/PY/NI to 3.3: Is it likely that missingness in the outcome depended on its true value?	NA
**Risk-of-bias judgment**		Low
**4**	**Bias in measurement of the outcome**	
4.1	Was the method of measuring the outcome inappropriate?	N
	Note: *Local Tumor Progression (LTP) is an objective outcome, assessed by imaging (CT) according to standard radiological criteria.*	
4.2	Could measurement or ascertainment of the outcome have differed between intervention groups?	N
	Note: *The same follow-up imaging protocol was applied uniformly to both groups.*	
4.3	If N/PN/NI to 4.1 and 4.2: Were outcome assessors aware of the intervention received by study participants?	Y
	Note: *The outcome assessor (Y.H.) was likely aware of the treatment group, as the appearance of the ablation zone with or without iodized oil from TACE would be significantly different on CT.*	
4.4	If Y/PY/NI to 4.3: Could assessment of the outcome have been influenced by knowledge of intervention received?	PN
	Note: *Although the outcome assessor was likely not blinded, the primary outcome (LTP on CT) is a reasonably objective radiological finding, making significant bias less likely.*	
4.5	If Y/PY/NI to 4.4: Is it likely that assessment of the outcome was influenced by knowledge of intervention received?	NA
**Risk-of-bias judgment**		Low
**5**	**Bias in selection of the reported result**	
5.1	Were the data that produced this result analysed in accordance with a prespecified analysis plan that was finalised before unblinded outcome data were available for analysis?	PN
	Note: *There is no mention of a pre-registered protocol or statistical analysis plan. The outcomes and analyses reported are standard for this type of trial, but without a pre-specified plan, the potential for selective reporting cannot be ruled out.*	
Is the numerical result being assessed likely to have been selected, on the basis of the results, from:		
5.2	Multiple eligible outcome measurements (eg, scales, definitions, time points) within the outcome domain?	N
	Note: *The primary outcome (local tumor progression) is clearly defined. No evidence suggests selective reporting from multiple measurements.*	
5.3	Multiple eligible analyses of the data?	N
	Note: *The primary analysis (Kaplan-Meier with log-rank test) is standard. No indication of result-dependent selection from multiple analytic approaches.*	
**Risk-of-bias judgment**		Some concerns
**Overall bias**		High

Song2024

**Title:** Laparoscopic liver resection versus radiofrequency ablation for small hepatocellular carcinoma: randomized clinical trial		
**Authors:** Juxian Song, Li Cao, Kuansheng Ma, *et al.*		**Year:** 2024
**Interventions:** Radiofrequency ablation (RFA) vs. Surgical resection (SR)		
**Registration:** NCT02243384		
**1**	**Bias arising from the randomisation process**	
1.1	Was the allocation sequence random?<br>***Note:*** *The manuscript states, "a computer-generated randomization sequence was placed in sequentially numbered, opaque, sealed envelopes by staff outside the study team at the data centre". This describes a proper random allocation sequence.*	Y
1.2	Was the allocation sequence concealed until participants were enrolled and assigned to interventions?<br>***Note:*** *The use of sequentially numbered, opaque, sealed envelopes, managed by staff outside the study team, constitutes adequate allocation concealment.*	Y
1.3	Did baseline differences between intervention groups suggest a problem with the randomisation process?<br>***Note:*** *Though Table 1 shows some differences in baseline characteristics between the two groups, the difference is likely due to chance rather than unsuccessful randomization.*	PN
**Risk-of-bias judgment**		Low
**2**	**Bias due to deviations from intended interventions**	
2.1	Were participants aware of their assigned intervention during the trial?<br>***Note:*** *Due to the nature of the interventions (laparoscopic surgery vs. percutaneous ablation), participants were necessarily aware of their treatment assignment.*	Y
2.2	Were carers and people delivering the interventions aware of participants' assigned intervention during the trial?<br>***Note:*** *The surgical and interventional radiology teams performing the procedures were aware of the treatment assignment.*	Y
2.3	If Y/PY/NI to 2.1 or 2.2: Were there deviations from the intended intervention that arose because of the trial context?<br>***Note:*** *Though some patients didn't receive the allocated treatment, the overall outcome won't be significantly influenced.*	PN
2.4	If Y/PY/NI to 2.3: Were these deviations likely to have affected the outcome?	NA
2.5	If Y/PY to 2.4: Were these deviations from intended intervention balanced between groups?	NA
2.6	Was an appropriate analysis used to estimate the effect of assignment to intervention?<br>***Note:*** *The primary analysis was performed on an intention-to-treat (ITT) basis, which is appropriate for RCTs and handles crossovers correctly.*	Y
2.7	If N/PN/NI to 2.6: Was there potential for a substantial impact (on the result) of the failure to analyse participants in the group to which they were randomised?	NA
**Risk-of-bias judgment**		Low
**3**	**Bias due to missing outcome data**	
3.1	Were data for this outcome available for all, or nearly all, participants randomised?	Y

Song2024 *(continued)*

	Note: *Of the 150 randomised patients, 3 were lost to follow-up (2 in LLR, 1 in RFA group), representing a very low attrition rate (2%). The primary outcome (overall survival) was available for the vast majority.*	
3.2	If N/PN/NI to 3.1: Is there evidence that the result was not biased by missing outcome data?	NA
3.3	If N/PN to 3.2: Could missingness in the outcome depend on its true value?	NA
3.4	If Y/PY/NI to 3.3: Is it likely that missingness in the outcome depended on its true value?	NA
**Risk-of-bias judgment**		Low
**4**	**Bias in measurement of the outcome**	
4.1	Was the method of measuring the outcome inappropriate?	N
	Note: *Overall survival and recurrence-free survival are objective, hard endpoints.*	
4.2	Could measurement or ascertainment of the outcome have differed between intervention groups?	N
	Note: *The same follow-up imaging protocol was applied uniformly to both groups.*	
4.3	If N/PN/NI to 4.1 and 4.2: Were outcome assessors aware of the intervention received by study participants?	Y
	Note: *The outcome assessors (surgeons and radiologists) were likely aware of the treatment group.*	
4.4	If Y/PY/NI to 4.3: Could assessment of the outcome have been influenced by knowledge of intervention received?	PN
	Note: *Although the outcome assessors were likely not blinded, the primary outcomes (death, radiologically confirmed recurrence) are objective and unlikely to be influenced by knowledge of the intervention.*	
4.5	If Y/PY/NI to 4.4: Is it likely that assessment of the outcome was influenced by knowledge of intervention received?	NA
**Risk-of-bias judgment**		Low
**5**	**Bias in selection of the reported result**	
5.1	Were the data that produced this result analysed in accordance with a prespecified analysis plan that was finalised before unblinded outcome data were available for analysis?	Y
	Note: *The study was registered on ClinicalTrials.gov (NCT02243384). The primary and secondary outcomes specified in the manuscript align with the registry record. The statistical methods (Kaplan-Meier, Cox regression) are standard for this type of trial.*	
Is the numerical result being assessed likely to have been selected, on the basis of the results, from:		
5.2	Multiple eligible outcome measurements (eg, scales, definitions, time points) within the outcome domain?	N
	Note: *The outcomes are clearly defined (overall survival, recurrence-free survival). No evidence suggests selective reporting from multiple measurements.*	
5.3	Multiple eligible analyses of the data?	N
	Note: *The primary analysis (Kaplan-Meier with log-rank test) is standard. Additional multivariable analyses are justified and complementary. No indication of result-dependent selection from multiple analytic approaches.*	
**Risk-of-bias judgment**		Low
**Overall bias**		Low

Sugimoto2025

**Title:** Microwave ablation vs. single-needle radiofrequency ablation for the treatment of HCC up to 4 cm: A randomized-controlled trial		
**Authors:** Katsutoshi Sugimoto, Kento Imajo, Hidekatsu Kuroda, *et al.*		**Year:** 2025
**Interventions:** Microwave ablation (MWA) vs. Radiofrequency ablation (RFA)		
**Registration:** UMIN000033297		
**1**	**Bias arising from the randomisation process**	
1.1	Was the allocation sequence random?<br>***Note:*** *The authors state that randomization was performed using a "fixed-block method (block size, 8)", which is a valid random allocation sequence.*	Y
1.2	Was the allocation sequence concealed until participants were enrolled and assigned to interventions?<br>***Note:*** *Although not explicitly described, allocation was performed by a non-clinician not involved in patient care, suggesting that concealment was likely maintained until assignment.*	Y
1.3	Did baseline differences between intervention groups suggest a problem with the randomisation process?<br>***Note:*** *Baseline characteristics (patient and tumor factors) were well-balanced between the MWA and RFA groups, as shown in Tables 1 and 2, indicating no issue with the randomization process.*	PN
**Risk-of-bias judgment**		Low
**2**	**Bias due to deviations from intended interventions**	
2.1	Were participants aware of their assigned intervention during the trial?<br>***Note:*** *The authors state that "the patients were blinded to the treatment", implying they were not aware of their assigned intervention.*	Y
2.2	Were carers and people delivering the interventions aware of participants' assigned intervention during the trial?<br>***Note:*** *The physicians were not blinded due to the different devices used for MWA and RFA.*	Y
2.3	If Y/PY/NI to 2.1 or 2.2: Were there deviations from the intended intervention that arose because of the trial context?<br>***Note:*** *There is no indication that the trial context led to deviations from the intended interventions. All patients received the allocated treatment.*	PN
2.4	If Y/PY/NI to 2.3: Were these deviations likely to have affected the outcome?	NA
2.5	If Y/PY to 2.4: Were these deviations from intended intervention balanced between groups?	NA
2.6	Was an appropriate analysis used to estimate the effect of assignment to intervention?<br>***Note:*** *The analysis was performed on an intention-to-treat basis where possible, and per-protocol for the primary outcome. Statistical methods were appropriate (Fisher exact test, Kaplan-Meier, Cox regression).*	Y
2.7	If N/PN/NI to 2.6: Was there potential for a substantial impact (on the result) of the failure to analyse participants in the group to which they were randomised?	NA
**Risk-of-bias judgment**		Low
**3**	**Bias due to missing outcome data**	
3.1	Were data for this outcome available for all, or nearly all, participants randomised?<br>***Note:*** *Data were available for 236/240 randomised participants (98.3%). Nine patients (19 lesions) were lost to follow-up for the primary endpoint, but this represents a small proportion (7.1% of lesions).*	Y

Sugimoto2025 *(continued)*

3.2	If N/PN/NI to 3.1: Is there evidence that the result was not biased by missing outcome data?	NA
3.3	If N/PN to 3.2: Could missingness in the outcome depend on its true value?	NA
3.4	If Y/PY/NI to 3.3: Is it likely that missingness in the outcome depended on its true value?	NA
**Risk-of-bias judgment**		Low
**4**	**Bias in measurement of the outcome**	
4.1	Was the method of measuring the outcome inappropriate?	N
	Note: *Local tumor progression (LTP) was assessed via contrast-enhanced CT or MRI, which is a standard and objective imaging-based method.*	
4.2	Could measurement or ascertainment of the outcome have differed between intervention groups?	N
	Note: *The same imaging modalities and diagnostic criteria were applied to both groups. Assessments were conducted at each center with consensus in case of disagreement.*	
4.3	If N/PN/NI to 4.1 and 4.2: Were outcome assessors aware of the intervention received by study participants?	Y
	Note: *Although not explicitly stated, radiologists and clinicians were likely aware of the treatment group due to the nature of the ablation zones visible on imaging.*	
4.4	If Y/PY/NI to 4.3: Could assessment of the outcome have been influenced by knowledge of intervention received?	PN
	Note: *Although assessors were likely unblinded, the outcome (LTP) was based on objective radiological criteria, reducing the risk of assessment bias.*	
4.5	If Y/PY/NI to 4.4: Is it likely that assessment of the outcome was influenced by knowledge of intervention received?	NA
**Risk-of-bias judgment**		Low
**5**	**Bias in selection of the reported result**	
5.1	Were the data that produced this result analysed in accordance with a prespecified analysis plan that was finalised before unblinded outcome data were available for analysis?	PY
	Note: *The trial was registered (UMIN000033297), and the statistical methods section describes pre-specified endpoints and analyses. However, it is not explicitly stated that the analysis plan was finalized before unblinding.*	
Is the numerical result being assessed likely to have been selected, on the basis of the results, from:		
5.2	Multiple eligible outcome measurements (eg, scales, definitions, time points) within the outcome domain?	N
	Note: *The primary outcome (LTP at 2 years) was clearly pre-specified. There is no evidence of selective reporting from multiple measurements.*	
5.3	Multiple eligible analyses of the data?	N
	Note: *The authors used pre-specified statistical methods. There is no indication of data dredging or selective reporting from multiple analyses.*	
**Risk-of-bias judgment**		Low
**Overall bias**		Low

Suh2021

**Title:** No-Touch vs. Conventional Radiofrequency Ablation Using Twin Internally Cooled Wet Electrodes for Small Hepatocellular Carcinomas: A Randomized Prospective Comparative Study		
**Authors:** Yun Seok Suh, Jae Won Choi, Jeong Hee Yoon, *et al.*		**Year:** 2021
**Interventions:** No-Touch Radiofrequency ablation (NT-RFA) vs. RFA		
**Registration:** NCT02806076		
**1**	**Bias arising from the randomisation process**	
1.1	Was the allocation sequence random?	Y
	Note: *The authors used a web-based randomization service with stratified block randomization, which ensures a random allocation sequence.*	
1.2	Was the allocation sequence concealed until participants were enrolled and assigned to interventions?	Y
	Note: *The use of a web-based randomization service managed by an independent center ensures adequate concealment of the allocation sequence until assignment.*	
1.3	Did baseline differences between intervention groups suggest a problem with the randomisation process?	PN
	Note: *Baseline characteristics were well-balanced between the two groups, as shown in Table 1, indicating no issues with the randomization process.*	
**Risk-of-bias judgment**		Low
**2**	**Bias due to deviations from intended interventions**	
2.1	Were participants aware of their assigned intervention during the trial?	Y
	Note: *The authors state that "study participants were blinded to group assignments".*	
2.2	Were carers and people delivering the interventions aware of participants' assigned intervention during the trial?	Y
	Note: *The interventional radiologist performing the procedures was necessarily aware of the assigned technique due to the nature of the interventions.*	
2.3	If Y/PY/NI to 2.1 or 2.2: Were there deviations from the intended intervention that arose because of the trial context?	PN
	Note: *Four patients in the NT-RFA group were converted to conventional RFA due to technical challenges, which represents a possible deviation from the intended intervention. However, considering the small ratio of these patients in all patients, we didn't think it would change the outcome significantly.*	
2.4	If Y/PY/NI to 2.3: Were these deviations likely to have affected the outcome?	NA
2.5	If Y/PY to 2.4: Were these deviations from intended intervention balanced between groups?	NA
2.6	Was an appropriate analysis used to estimate the effect of assignment to intervention?	Y
	Note: *Both intention-to-treat and as-treated analyses were performed, and appropriate statistical methods were used to account for deviations.*	
2.7	If N/PN/NI to 2.6: Was there potential for a substantial impact (on the result) of the failure to analyse participants in the group to which they were randomised?	NA
**Risk-of-bias judgment**		Low
**3**	**Bias due to missing outcome data**	
3.1	Were data for this outcome available for all, or nearly all, participants randomised?	Y
	Note: *All 73 randomised participants were included in the analysis, and there were no losses to follow-up, as stated in Figure 1.*	

Suh2021 *(continued)*

3.2	If N/PN/NI to 3.1: Is there evidence that the result was not biased by missing outcome data?	NA
3.3	If N/PN to 3.2: Could missingness in the outcome depend on its true value?	NA
3.4	If Y/PY/NI to 3.3: Is it likely that missingness in the outcome depended on its true value?	NA
**Risk-of-bias judgment**		Low
**4**	**Bias in measurement of the outcome**	
4.1	Was the method of measuring the outcome inappropriate?<br>***Note:*** *LTP was assessed using contrast-enhanced CT or MRI, which is a standard and objective imaging-based method.*	N
4.2	Could measurement or ascertainment of the outcome have differed between intervention groups?<br>***Note:*** *The same imaging modalities and diagnostic criteria were applied uniformly to both groups.*	N
4.3	If N/PN/NI to 4.1 and 4.2: Were outcome assessors aware of the intervention received by study participants?<br>***Note:*** *The authors explicitly state that "those assessing outcomes were blinded to group assignments".*	Y
4.4	If Y/PY/NI to 4.3: Could assessment of the outcome have been influenced by knowledge of intervention received?<br>***Note:*** *Although assessors were likely unblinded, the outcome (LTP) was based on objective radiological criteria, reducing the risk of assessment bias.*	PN
4.5	If Y/PY/NI to 4.4: Is it likely that assessment of the outcome was influenced by knowledge of intervention received?	NA
**Risk-of-bias judgment**		Low
**5**	**Bias in selection of the reported result**	
5.1	Were the data that produced this result analysed in accordance with a prespecified analysis plan that was finalised before unblinded outcome data were available for analysis?<br>***Note:*** *The trial was registered (NCT02806076), and the statistical methods section describes pre-specified endpoints and analyses, including both intention-to-treat and as-treated analyses.*	PY
Is the numerical result being assessed likely to have been selected, on the basis of the results, from:		
5.2	Multiple eligible outcome measurements (eg, scales, definitions, time points) within the outcome domain?<br>***Note:*** *The primary outcome (cumulative LTP rate) was clearly pre-specified, and there is no evidence of selection from multiple measurements.*	N
5.3	Multiple eligible analyses of the data?<br>***Note:*** *The authors used pre-specified statistical methods, and both intention-to-treat and as-treated analyses were planned, reducing the risk of selective reporting.*	N
**Risk-of-bias judgment**		Low
**Overall bias**		Low

Takayama2022

Title: Surgery versus Radiofrequency Ablation for Small Hepatocellular Carcinoma: A Randomized Controlled Trial (SURF Trial)

Authors: Tadatoshi Takayama, Kiyoshi Hasegawa, Namiki Izumi, *et al.* **Year:** 2022

Interventions: Radiofrequency ablation (RFA) vs. Surgical resection (SR)

Registration: UMIN000001795

**1**	**Bias arising from the randomisation process**	
1.1	Was the allocation sequence random?<br>***Note:*** *The authors used a probabilistic minimization method for randomization, which is an acceptable random allocation technique.*	Y
1.2	Was the allocation sequence concealed until participants were enrolled and assigned to interventions?<br>***Note:*** *The use of a web-based randomization service managed by an independent center ensures adequate concealment of the allocation sequence until assignment.*	Y
1.3	Did baseline differences between intervention groups suggest a problem with the randomisation process?<br>***Note:*** *Baseline characteristics were well-balanced between the surgery and RFA groups, as shown in Table 1, indicating no issues with the randomization process.*	PN
**Risk-of-bias judgment**		Low
**2**	**Bias due to deviations from intended interventions**	
2.1	Were participants aware of their assigned intervention during the trial?<br>***Note:*** *Although not explicitly stated, participants were likely blinded to treatment allocation due to the nature of the interventions and the use of a centralized randomization system.*	N
2.2	Were carers and people delivering the interventions aware of participants' assigned intervention during the trial?<br>***Note:*** *Surgeons and interventional radiologists were necessarily aware of the assigned treatment due to the nature of the procedures.*	Y
2.3	If Y/PY/NI to 2.1 or 2.2: Were there deviations from the intended intervention that arose because of the trial context?<br>***Note:*** *Five patients in the surgery group and three in the RFA group did not receive the allocated treatment, representing deviations from the intended intervention. However, considering the small ratio of these patients in all patients, we didn't think it would change the outcome significantly.*	PN
2.4	If Y/PY/NI to 2.3: Were these deviations likely to have affected the outcome?	NA
2.5	If Y/PY to 2.4: Were these deviations from intended intervention balanced between groups?	NA
2.6	Was an appropriate analysis used to estimate the effect of assignment to intervention?<br>***Note:*** *The primary analysis was performed on an intention-to-treat basis, which is appropriate for estimating the effect of assignment to intervention.*	Y
2.7	If N/PN/NI to 2.6: Was there potential for a substantial impact (on the result) of the failure to analyse participants in the group to which they were randomised?	NA
**Risk-of-bias judgment**		Low
**3**	**Bias due to missing outcome data**	
3.1	Were data for this outcome available for all, or nearly all, participants randomised?<br>***Note:*** *Data were available for 301/308 randomised patients (97.7%). Loss to follow-up was 6% in the surgery group and 4% in the RFA group, which is acceptable for time-to-event outcomes.*	Y

Takayama2022 *(continued)*

3.2	If N/PN/NI to 3.1: Is there evidence that the result was not biased by missing outcome data?	NA
3.3	If N/PN to 3.2: Could missingness in the outcome depend on its true value?	NA
3.4	If Y/PY/NI to 3.3: Is it likely that missingness in the outcome depended on its true value?	NA
**Risk-of-bias judgment**		Low
**4**	**Bias in measurement of the outcome**	
4.1	Was the method of measuring the outcome inappropriate?<br>***Note:*** *Recurrence-free survival (RFS) was assessed using standard imaging (CT/MRI) and clinical criteria, which is appropriate.*	N
4.2	Could measurement or ascertainment of the outcome have differed between intervention groups?<br>***Note:*** *The same follow-up protocol and diagnostic criteria were applied to both groups.*	N
4.3	If N/PN/NI to 4.1 and 4.2: Were outcome assessors aware of the intervention received by study participants?<br>***Note:*** *Although not explicitly stated, the use of objective imaging criteria and centralized follow-up suggests that outcome assessors were likely blinded to treatment allocation.*	Y
4.4	If Y/PY/NI to 4.3: Could assessment of the outcome have been influenced by knowledge of intervention received?<br>***Note:*** *Although assessors were likely unblinded, the outcome (LTP) was based on objective radiological criteria, reducing the risk of assessment bias.*	PN
4.5	If Y/PY/NI to 4.4: Is it likely that assessment of the outcome was influenced by knowledge of intervention received?	NA
**Risk-of-bias judgment**		Low
**5**	**Bias in selection of the reported result**	
5.1	Were the data that produced this result analysed in accordance with a prespecified analysis plan that was finalised before unblinded outcome data were available for analysis?<br>***Note:*** *The trial was registered (UMIN000001795), and the statistical analysis plan was pre-specified, including the use of intention-to-treat analysis and Kaplan-Meier methods.*	PY
Is the numerical result being assessed likely to have been selected, on the basis of the results, from:		
5.2	Multiple eligible outcome measurements (eg, scales, definitions, time points) within the outcome domain?<br>***Note:*** *The primary outcome (RFS) was clearly pre-specified in the protocol. There is no evidence of selection from multiple measurements.*	N
5.3	Multiple eligible analyses of the data?<br>***Note:*** *The authors used pre-specified statistical methods. Subgroup analyses were planned and reported, but there is no indication of data dredging.*	N
**Risk-of-bias judgment**		Low
**Overall bias**		Low

Vietti2018

**Title:** Efficacy of microwave ablation versus radiofrequency ablation for the treatment of hepatocellular carcinoma in patients with chronic liver disease: a randomised controlled phase 2 trial		
**Authors:** Vietti Violi, Rafael Duran, Boris Guiu, *et al.*		**Year:** 2021
**Interventions:** Microwave ablation (MWA) vs. Radiofrequency ablation (RFA)		
**Registration:** NCT02859753		
**1**	**Bias arising from the randomisation process**	
1.1	Was the allocation sequence random?<br>***Note:*** *Allocation was done using sealed opaque envelopes, which is a generally acceptable method, though not as robust as a centralized electronic system.*	PY
1.2	Was the allocation sequence concealed until participants were enrolled and assigned to interventions?<br>***Note:*** *Allocation was done using sealed opaque envelopes, which is a generally acceptable method, though not as robust as a centralized electronic system.*	Y
1.3	Did baseline differences between intervention groups suggest a problem with the randomisation process?<br>***Note:*** *Baseline characteristics were well-balanced between the two groups, as shown in the manuscript, indicating no issues with the randomization process.*	PN
**Risk-of-bias judgment**		Low
**2**	**Bias due to deviations from intended interventions**	
2.1	Were participants aware of their assigned intervention during the trial?<br>***Note:*** *The authors state that "patients were masked to the treatment".*	N
2.2	Were carers and people delivering the interventions aware of participants' assigned intervention during the trial?<br>***Note:*** *Physicians were not blinded due to the different devices used.*	Y
2.3	If Y/PY/NI to 2.1 or 2.2: Were there deviations from the intended intervention that arose because of the trial context?<br>***Note:*** *Six patients did not receive the allocated treatment, and two were lost to follow-up immediately after intervention. However, the number of patients lost to follow-up was not large, so no significant deviation exists.*	Y
2.4	If Y/PY/NI to 2.3: Were these deviations likely to have affected the outcome?	NA
2.5	If Y/PY to 2.4: Were these deviations from intended intervention balanced between groups?	NA
2.6	Was an appropriate analysis used to estimate the effect of assignment to intervention?<br>***Note:*** *The primary analysis was performed on a per-protocol basis rather than intention-to-treat, which may introduce bias.*	PN
2.7	If N/PN/NI to 2.6: Was there potential for a substantial impact (on the result) of the failure to analyse participants in the group to which they were randomised?<br>***Note:*** *Considering the small portion of patients who were lost to follow-up, we suppose that the bias won't be influenced significantly.*	PN
**Risk-of-bias judgment**		Some concerns
**3**	**Bias due to missing outcome data**	
3.1	Were data for this outcome available for all, or nearly all, participants randomised?<br>***Note:*** *Data were available for 144/152 randomised patients (94.7%). Loss to follow-up was minimal (2 patients).*	Y

Vietti2018 *(continued)*

3.2	If N/PN/NI to 3.1: Is there evidence that the result was not biased by missing outcome data?	NA
3.3	If N/PN to 3.2: Could missingness in the outcome depend on its true value?	NA
3.4	If Y/PY/NI to 3.3: Is it likely that missingness in the outcome depended on its true value?	NA
**Risk-of-bias judgment**		Low
**4**	**Bias in measurement of the outcome**	
4.1	Was the method of measuring the outcome inappropriate?<br>***Note:*** *Local tumor progression was assessed using standard imaging (CT/MRI) with central review by blinded radiologists, which is appropriate.*	N
4.2	Could measurement or ascertainment of the outcome have differed between intervention groups?<br>***Note:*** *The same imaging protocols and blinded central review were applied to both groups.*	N
4.3	If N/PN/NI to 4.1 and 4.2: Were outcome assessors aware of the intervention received by study participants?<br>***Note:*** *Although the central reviewers were masked, the local treating physicians were not, which could theoretically influence follow-up intensity, though this risk is low given the objective outcome.*	PY
4.4	If Y/PY/NI to 4.3: Could assessment of the outcome have been influenced by knowledge of intervention received?<br>***Note:*** *The use of blinded central review minimizes the risk of assessment bias.*	PN
4.5	If Y/PY/NI to 4.4: Is it likely that assessment of the outcome was influenced by knowledge of intervention received?	NA
**Risk-of-bias judgment**		Low
**5**	**Bias in selection of the reported result**	
5.1	Were the data that produced this result analysed in accordance with a prespecified analysis plan that was finalised before unblinded outcome data were available for analysis?<br>***Note:*** *The trial was registered (NCT02859753), and the statistical methods were pre-specified.*	Y
Is the numerical result being assessed likely to have been selected, on the basis of the results, from:		
5.2	Multiple eligible outcome measurements (eg, scales, definitions, time points) within the outcome domain?<br>***Note:*** *The primary outcome (local tumor progression at 2 years) was clearly pre-specified.*	N
5.3	Multiple eligible analyses of the data?<br>***Note:*** *The authors used pre-specified statistical methods. There is no indication of selective reporting from multiple analyses.*	N
**Risk-of-bias judgment**		Low
**Overall bias**		Some concerns

Vogl2024

**Title:** Comparison of Microwave and Radiofrequency Ablation for the Treatment of Small- and Medium-Sized Hepatocellular Carcinomas in a Prospective Randomized Trial		
**Authors:** Thomas J. Vogl, Simon S. Martin, Tatjana Gruber-Rouh, *et al.*		**Year:** 2024
**Interventions:** CT-guided percutaneous microwave (MWA) vs. radiofrequency ablation (RFA)		
**Registration:** NA		
**1**	**Bias arising from the randomisation process**	
1.1	Was the allocation sequence random?	Y
	Note: *The study is a randomized controlled trial and explicitly states the use of a permuted block randomization design.*	
1.2	Was the allocation sequence concealed until participants were enrolled and assigned to interventions?	PY
	Note: *Although randomisation was performed, the manuscript does not explicitly describe concealment of the allocation sequence. However, there is no evidence of selective enrolment, and baseline characteristics were balanced.*	
1.3	Did baseline differences between intervention groups suggest a problem with the randomisation process?	N
	Note: *Baseline characteristics including tumor size, grading, BCLC stage, and prior treatment were similar.*	
**Risk-of-bias judgment**		Low
**2**	**Bias due to deviations from intended interventions**	
2.1	Were participants aware of their assigned intervention during the trial?	Y
	Note: *Participants were likely aware of their assigned intervention (RFA or MWA), no blinding described.*	
2.2	Were carers and people delivering the interventions aware of participants' assigned intervention during the trial?	Y
	Note: *Interventional radiologists would know treatment type; no indication of blinding.*	
2.3	If Y/PY/NI to 2.1 or 2.2: Were there deviations from the intended intervention that arose because of the trial context?	N
	Note: *No deviations from intended interventions were reported.*	
2.4	If Y/PY/NI to 2.3: Were these deviations likely to have affected the outcome?	NA
2.5	If Y/PY to 2.4: Were these deviations from intended intervention balanced between groups?	NA
2.6	Was an appropriate analysis used to estimate the effect of assignment to intervention?	Y
	Note: *Analysis used Kaplan–Meier curves, log-rank tests, Cox regression, appropriate for group comparison.*	
2.7	If N/PN/NI to 2.6: Was there potential for a substantial impact (on the result) of the failure to analyse participants in the group to which they were randomised?	NA
**Risk-of-bias judgment**		Low
**3**	**Bias due to missing outcome data**	
3.1	Were data for this outcome available for all, or nearly all, participants randomised?	Y
	Note: *All patients were followed up regularly and survival data were reported.*	
3.2	If N/PN/NI to 3.1: Is there evidence that the result was not biased by missing outcome data?	NA
3.3	If N/PN to 3.2: Could missingness in the outcome depend on its true value?	NA
3.4	If Y/PY/NI to 3.3: Is it likely that missingness in the outcome depended on its true value?	NA

Vogl2024 *(continued)*

**Risk-of-bias judgment**		Low
**4**	**Bias in measurement of the outcome**	
4.1	Was the method of measuring the outcome inappropriate?	N
	Note: *Standardized MRI protocols and definitions used for outcome assessment.*	
4.2	Could measurement or ascertainment of the outcome have differed between intervention groups?	N
	Note: *All patients underwent the same imaging follow-up protocol regardless of group.*	
4.3	If N/PN/NI to 4.1 and 4.2: Were outcome assessors aware of the intervention received by study participants?	Y
	Note: *Assessors were likely aware of group allocation due to intervention-specific imaging features.*	
4.4	If Y/PY/NI to 4.3: Could assessment of the outcome have been influenced by knowledge of intervention received?	N
	Note: *Outcomes (tumor recurrence, survival) are objective and unlikely to be influenced.*	
4.5	If Y/PY/NI to 4.4: Is it likely that assessment of the outcome was influenced by knowledge of intervention received?	NA
**Risk-of-bias judgment**		Low
**5**	**Bias in selection of the reported result**	
5.1	Were the data that produced this result analysed in accordance with a prespecified analysis plan that was finalised before unblinded outcome data were available for analysis?	PY
	Note: *Though no registration number is mentioned, but the protocol was screened and approved in the ethical review.*	
Is the numerical result being assessed likely to have been selected, on the basis of the results, from:		
5.2	Multiple eligible outcome measurements (eg, scales, definitions, time points) within the outcome domain?	N
	Note: *Single method per outcome; no evidence of multiple timepoints or measures selectively reported.*	
5.3	Multiple eligible analyses of the data?	N
	Note: *Each endpoint analyzed with a single method; no multiple analytic approaches suggested.*	
**Risk-of-bias judgment**		Low
**Overall bias**		Low

Wang2015

Title: A Multicenter Randomized Controlled Trial of Percutaneous Cryoablation Versus Radiofrequency Ablation in Hepatocellular Carcinoma

Authors: Chunping Wang, Huaming Wang, Wuwei Yang, *et al.* **Year:** 2015

Interventions: Cryoablation ablation (CA) vs. Radiofrequency ablation (RFA)

Registration: 20071203T

**1**	**Bias arising from the randomisation process**	
1.1	Was the allocation sequence random?	Y
	Note: *The authors state that a computerized random number generator was used to create the allocation sequence.*	
1.2	Was the allocation sequence concealed until participants were enrolled and assigned to interventions?	PY
	Note: *Sealed, consecutively numbered envelopes were used. Although this is a form of concealment, the method of ensuring the envelopes were opaque and tamper-proof is not explicitly described.*	
1.3	Did baseline differences between intervention groups suggest a problem with the randomisation process?	N
	Note: *Baseline characteristics were well-balanced between groups, except for the number of tumors (p=0.04), which was adjusted for in the analysis. This minor imbalance is not considered indicative of a systematic problem with randomization.*	
**Risk-of-bias judgment**		Low
**2**	**Bias due to deviations from intended interventions**	
2.1	Were participants aware of their assigned intervention during the trial?	Y
	Note: *The nature of the percutaneous ablation procedures (cryoablation vs. RFA) makes blinding of participants impossible.*	
2.2	Were carers and people delivering the interventions aware of participants' assigned intervention during the trial?	Y
	Note: *The interventional radiologists performing the procedures were necessarily aware of the treatment allocation.*	
2.3	If Y/PY/NI to 2.1 or 2.2: Were there deviations from the intended intervention that arose because of the trial context?	N
	Note: *There is no indication that the trial context led to systematic deviations from the intended treatment protocols. A small number of patients in the RFA group with incomplete ablation received cryoablation as salvage therapy, but this reflects real-world clinical practice and was balanced between groups.*	
2.4	If Y/PY/NI to 2.3: Were these deviations likely to have affected the outcome?	NA
2.5	If Y/PY to 2.4: Were these deviations from intended intervention balanced between groups?	NA
2.6	Was an appropriate analysis used to estimate the effect of assignment to intervention?	Y
	Note: *An intention-to-treat analysis was performed, including all randomised patients.*	
2.7	If N/PN/NI to 2.6: Was there potential for a substantial impact (on the result) of the failure to analyse participants in the group to which they were randomised?	NA
**Risk-of-bias judgment**		Low
**3**	**Bias due to missing outcome data**	
3.1	Were data for this outcome available for all, or nearly all, participants randomised?	Y

Wang2015 *(continued)*

	Note: *Only 3 patients (0.83%) were lost to follow-up, and all randomised patients were included in the analysis.*	
3.2	If N/PN/NI to 3.1: Is there evidence that the result was not biased by missing outcome data?	NA
3.3	If N/PN to 3.2: Could missingness in the outcome depend on its true value?	NA
3.4	If Y/PY/NI to 3.3: Is it likely that missingness in the outcome depended on its true value?	NA
**Risk-of-bias judgment**		Low
**4**	**Bias in measurement of the outcome**	
4.1	Was the method of measuring the outcome inappropriate?	N
	Note: *Outcomes (LTP, survival) were assessed using standardised imaging (CT/MRI) and clinical follow-up, which are objective and appropriate.*	
4.2	Could measurement or ascertainment of the outcome have differed between intervention groups?	N
	Note: *The same follow-up protocol and imaging criteria were applied to both groups.*	
4.3	If N/PN/NI to 4.1 and 4.2: Were outcome assessors aware of the intervention received by study participants?	Y
	Note: *The radiologists assessing imaging outcomes were likely aware of the treatment type due to the characteristic appearance of cryoablation vs. RFA zones.*	
4.4	If Y/PY/NI to 4.3: Could assessment of the outcome have been influenced by knowledge of intervention received?	PN
	Note: *Although assessors were likely unblinded, the use of objective, pre-defined radiological criteria (e.g., enhancement patterns, size measurements) for recurrence and survival outcomes reduces the risk of detection bias.*	
4.5	If Y/PY/NI to 4.4: Is it likely that assessment of the outcome was influenced by knowledge of intervention received?	NA
**Risk-of-bias judgment**		Low
**5**	**Bias in selection of the reported result**	
5.1	Were the data that produced this result analysed in accordance with a prespecified analysis plan that was finalised before unblinded outcome data were available for analysis?	PY
	Note: *The trial was registered (20071203T), and the primary and secondary endpoints mentioned in the manuscript align with what would be expected in an ablation trial. However, a detailed statistical analysis plan (SAP) is not referenced, so full confirmation is not possible.*	
Is the numerical result being assessed likely to have been selected, on the basis of the results, from:		
5.2	Multiple eligible outcome measurements (eg, scales, definitions, time points) within the outcome domain?	N
	Note: *The outcomes reported (LTP, OS, TFS, complications) are standard and pre-specified. There is no evidence of selective reporting from multiple definitions.*	
5.3	Multiple eligible analyses of the data?	N
	Note: *The statistical methods (Kaplan-Meier, Cox regression) are standard and appropriate. There is no indication of data dredging or selection from multiple analytic approaches.*	
**Risk-of-bias judgment**		Low
**Overall bias**		Low

Wei2023

**Title:** Neoadjuvant intensity modulated radiotherapy for a single and small (⩽ 5 cm) hepatitis B virus-related hepatocellular carcinoma predicted to have high risks of microvascular invasion: a randomized clinical trial	
**Authors:** Xubiao Wei, Yabo Jiang, Shuang Feng, *et al.*	**Year:** 2015
**Interventions:** Radiotherapy (RT) + Surgical resection (SR) vs. SR	
**Registration:** ChiCTR-IOR-1400556	

**1**	**Bias arising from the randomisation process**	
1.1	Was the allocation sequence random?<br>***Note:*** *The authors state that a computer-generated randomization code was used for treatment group allocations.*	Y
1.2	Was the allocation sequence concealed until participants were enrolled and assigned to interventions?<br>***Note:*** *Although randomization was computer-generated, the method of allocation concealment (e.g., use of sealed opaque envelopes or a central system) is not explicitly described. The absence of baseline imbalances suggests adequate concealment was likely maintained.*	PY
1.3	Did baseline differences between intervention groups suggest a problem with the randomisation process?<br>***Note:*** *Baseline characteristics were well-balanced between the two groups, indicating successful randomization.*	N
**Risk-of-bias judgment**		Low
**2**	**Bias due to deviations from intended interventions**	
2.1	Were participants aware of their assigned intervention during the trial?<br>***Note:*** *The trial was open-label; participants were aware of their treatment assignment.*	Y
2.2	Were carers and people delivering the interventions aware of participants' assigned intervention during the trial?<br>***Note:*** *Clinicians delivering radiotherapy or performing surgery were necessarily aware of the treatment group.*	Y
2.3	If Y/PY/NI to 2.1 or 2.2: Were there deviations from the intended intervention that arose because of the trial context?<br>***Note:*** *Three patients in the neoadjuvant RT group deviated from the protocol (two had upfront surgery, one had RFA after RT). However, compared with the sample size, the number of patients who changed the protocol was not significant.*	PN
2.4	If Y/PY/NI to 2.3: Were these deviations likely to have affected the outcome?	NA
2.5	If Y/PY to 2.4: Were these deviations from intended intervention balanced between groups?	NA
2.6	Was an appropriate analysis used to estimate the effect of assignment to intervention?<br>***Note:*** *Both intention-to-treat and per-protocol analyses were performed, and the results were consistent.*	Y
2.7	If N/PN/NI to 2.6: Was there potential for a substantial impact (on the result) of the failure to analyse participants in the group to which they were randomised?	NA
**Risk-of-bias judgment**		Low
**3**	**Bias due to missing outcome data**	
3.1	Were data for this outcome available for all, or nearly all, participants randomised?<br>***Note:*** *Only 3 patients (1 in RT group, 2 in surgery group) were lost to follow-up, and all randomized patients were included in the ITT analysis.*	Y

Wei2023 *(continued)*

3.2	If N/PN/NI to 3.1: Is there evidence that the result was not biased by missing outcome data?	NA
3.3	If N/PN to 3.2: Could missingness in the outcome depend on its true value?	NA
3.4	If Y/PY/NI to 3.3: Is it likely that missingness in the outcome depended on its true value?	NA
**Risk-of-bias judgment**		Low
**4**	**Bias in measurement of the outcome**	
4.1	Was the method of measuring the outcome inappropriate? ***Note:*** *Outcomes (DFS, OS) were assessed using standard clinical and radiological follow-up, which are objective and appropriate.*	N
4.2	Could measurement or ascertainment of the outcome have differed between intervention groups? ***Note:*** *The same follow-up protocol was applied to both groups.*	N
4.3	If N/PN/NI to 4.1 and 4.2: Were outcome assessors aware of the intervention received by study participants? ***Note:*** *The trial was open-label; outcome assessors were likely aware of the treatment assignment.*	Y
4.4	If Y/PY/NI to 4.3: Could assessment of the outcome have been influenced by knowledge of intervention received? ***Note:*** *Although assessors were unblinded, the primary outcomes (DFS, OS) are objective and based on imaging and survival data, reducing the risk of detection bias.*	PN
4.5	If Y/PY/NI to 4.4: Is it likely that assessment of the outcome was influenced by knowledge of intervention received?	NA
**Risk-of-bias judgment**		Low
**5**	**Bias in selection of the reported result**	
5.1	Were the data that produced this result analysed in accordance with a prespecified analysis plan that was finalised before unblinded outcome data were available for analysis? ***Note:*** *The trial was registered (ChiCTR-IOR-1400556), and the primary and secondary endpoints mentioned in the manuscript align with the registration. However, a detailed statistical analysis plan (SAP) is not referenced.*	PY
Is the numerical result being assessed likely to have been selected, on the basis of the results, from:		
5.2	Multiple eligible outcome measurements (eg, scales, definitions, time points) within the outcome domain? ***Note:*** *The outcomes reported (DFS, OS) are standard and pre-specified. There is no evidence of selective reporting from multiple definitions.*	N
5.3	Multiple eligible analyses of the data? ***Note:*** *The statistical methods (Kaplan-Meier, log-rank, Cox regression) are standard and appropriate. There is no indication of data dredging or selection from multiple analytic approaches.*	N
**Risk-of-bias judgment**		Low
**Overall bias**		Low

Xi2024

**Title:** Radiofrequency Ablation Versus Stereotactic Body Radiotherapy for Recurrent Small Hepatocellular Carcinoma: A Randomized, Open-Label, Controlled Trial		
**Authors:** Mian Xi, Zhoutian Yang, Li Hu, *et al.*		**Year:** 2024
**Interventions:** Radiotherapy (RT) vs. Radiofrequency ablation (RFA)		
**Registration:** NCT04047173		
**1**	**Bias arising from the randomisation process**	
1.1	Was the allocation sequence random?	Y
	Note: *The authors state that a computer-generated random assignment system was used.*	
1.2	Was the allocation sequence concealed until participants were enrolled and assigned to interventions?	PY
	Note: *Although randomization was computer-generated, the method of allocation concealment (e.g., sealed envelopes or a centralized system) is not explicitly described. However, the baseline characteristics were well-balanced, suggesting adequate concealment was likely maintained.*	
1.3	Did baseline differences between intervention groups suggest a problem with the randomisation process?	N
	Note: *Baseline characteristics were well-balanced between the two groups, indicating successful randomization.*	
**Risk-of-bias judgment**		Low
**2**	**Bias due to deviations from intended interventions**	
2.1	Were participants aware of their assigned intervention during the trial?	Y
	Note: *The trial was open-label; participants were aware of their treatment assignment.*	
2.2	Were carers and people delivering the interventions aware of participants' assigned intervention during the trial?	Y
	Note: *Clinicians performing RFA or delivering SBRT were necessarily aware of the treatment group.*	
2.3	If Y/PY/NI to 2.1 or 2.2: Were there deviations from the intended intervention that arose because of the trial context?	PN
	Note: *All patients completed the assigned treatments according to the protocol with no treatment discontinuation.*	
2.4	If Y/PY/NI to 2.3: Were these deviations likely to have affected the outcome?	NA
2.5	If Y/PY to 2.4: Were these deviations from intended intervention balanced between groups?	NA
2.6	Was an appropriate analysis used to estimate the effect of assignment to intervention?	Y
	Note: *An intention-to-treat analysis was performed.*	
2.7	If N/PN/NI to 2.6: Was there potential for a substantial impact (on the result) of the failure to analyse participants in the group to which they were randomised?	NA
**Risk-of-bias judgment**		Low
**3**	**Bias due to missing outcome data**	
3.1	Were data for this outcome available for all, or nearly all, participants randomised?	Y
	Note: *All 166 randomized patients were included in the ITT analysis, and follow-up was complete for at least 2 years.*	
3.2	If N/PN/NI to 3.1: Is there evidence that the result was not biased by missing outcome data?	NA
3.3	If N/PN to 3.2: Could missingness in the outcome depend on its true value?	NA
3.4	If Y/PY/NI to 3.3: Is it likely that missingness in the outcome depended on its true value?	NA

Xi2024_(continued)_

**Risk-of-bias judgment**		Low
**4**	**Bias in measurement of the outcome**	
4.1	Was the method of measuring the outcome inappropriate?<br>***Note:*** *Outcomes (LPFS, PFS, OS) were assessed using standard imaging (MRI/CT) and clinical criteria, which are objective and appropriate.*	N
4.2	Could measurement or ascertainment of the outcome have differed between intervention groups?<br>***Note:*** *The same follow-up protocol and imaging criteria were applied to both groups.*	N
4.3	If N/PN/NI to 4.1 and 4.2: Were outcome assessors aware of the intervention received by study participants?<br>***Note:*** *The radiologists evaluating imaging outcomes were blinded to patient outcomes and treatment assignments.*	N
4.4	If Y/PY/NI to 4.3: Could assessment of the outcome have been influenced by knowledge of intervention received?	NA
4.5	If Y/PY/NI to 4.4: Is it likely that assessment of the outcome was influenced by knowledge of intervention received?	NA
**Risk-of-bias judgment**		Low
**5**	**Bias in selection of the reported result**	
5.1	Were the data that produced this result analysed in accordance with a prespecified analysis plan that was finalised before unblinded outcome data were available for analysis?<br>***Note:*** *The trial was registered (NCT04047173), and the primary and secondary endpoints align with the registration. The statistical methods are clearly described and appropriate.*	Y
Is the numerical result being assessed likely to have been selected, on the basis of the results, from:		
5.2	Multiple eligible outcome measurements (eg, scales, definitions, time points) within the outcome domain?<br>***Note:*** *The outcomes reported (LPFS, PFS, OS, LCR) are standard and pre-specified. There is no evidence of selective reporting from multiple definitions.*	N
5.3	Multiple eligible analyses of the data?<br>***Note:*** *The statistical methods (Kaplan-Meier, log-rank, Cox regression) are standard and pre-specified. There is no indication of data dredging or selection from multiple analytic approaches.*	N
**Risk-of-bias judgment**		Low
**Overall bias**		Low

Yu2017

**Title:** Percutaneous cooled-probe microwave versus radiofrequency ablation in early-stage hepatocellular carcinoma: a phase III randomised controlled trial		
**Authors:** Jie Yu, Xiaoling Yu, Zhiyu Han, *et al.*		**Year:** 2017
**Interventions:** Microwave ablation (MWA) vs. Radiofrequency ablation (RFA)		
**Registration:** NCT02539212		
**1**	**Bias arising from the randomisation process**	
1.1	Was the allocation sequence random?	Y
	Note: *The study is described as a phase III randomised controlled trial (RCT). Although the specific method of sequence generation is not detailed, the term "randomised" implies a random process.*	
1.2	Was the allocation sequence concealed until participants were enrolled and assigned to interventions?	PY
	Note: *The manuscript does not describe the method of allocation concealment. However, according to the information published in the registration platform, we supposed that rational randomization method was used.*	
1.3	Did baseline differences between intervention groups suggest a problem with the randomisation process?	N
	Note: *The demographics and preablation liver function tests were reported to be similar between groups, and tumour characteristics (size, location) were well-balanced.*	
**Risk-of-bias judgment**		Low
**2**	**Bias due to deviations from intended interventions**	
2.1	Were participants aware of their assigned intervention during the trial?	Y
	Note: *The nature of the percutaneous ablation procedures makes blinding of participants impractical.*	
2.2	Were carers and people delivering the interventions aware of participants' assigned intervention during the trial?	Y
	Note: *The interventional radiologists performing the procedures were necessarily aware of the treatment type.*	
2.3	If Y/PY/NI to 2.1 or 2.2: Were there deviations from the intended intervention that arose because of the trial context?	N
	Note: *There is no indication that the trial context led to systematic deviations from the intended protocols.*	
2.4	If Y/PY/NI to 2.3: Were these deviations likely to have affected the outcome?	NA
2.5	If Y/PY to 2.4: Were these deviations from intended intervention balanced between groups?	NA
2.6	Was an appropriate analysis used to estimate the effect of assignment to intervention?	Y
	Note: *An intention-to-treat analysis was implied, as all randomised patients were included in the analysis.*	
2.7	If N/PN/NI to 2.6: Was there potential for a substantial impact (on the result) of the failure to analyse participants in the group to which they were randomised?	NA
**Risk-of-bias judgment**		Low
**3**	**Bias due to missing outcome data**	
3.1	Were data for this outcome available for all, or nearly all, participants randomised?	Y

	Note: *All 403 randomised patients (203 in MWA, 200 in RFA) were included in the analysis. The median follow-up was 35.2 months, and no loss to follow-up is mentioned.*	
3.2	If N/PN/NI to 3.1: Is there evidence that the result was not biased by missing outcome data?	NA
3.3	If N/PN to 3.2: Could missingness in the outcome depend on its true value?	NA
3.4	If Y/PY/NI to 3.3: Is it likely that missingness in the outcome depended on its true value?	NA
**Risk-of-bias judgment**		Low
**4**	**Bias in measurement of the outcome**	
4.1	Was the method of measuring the outcome inappropriate?	N
	Note: *Outcomes (technical effectiveness, local progression, survival) were assessed using standard imaging (ultrasound, CT/MRI) and clinical follow-up, which are objective and appropriate.*	
4.2	Could measurement or ascertainment of the outcome have differed between intervention groups?	N
	Note: *The same follow-up protocol and imaging criteria were applied to both groups.*	
4.3	If N/PN/NI to 4.1 and 4.2: Were outcome assessors aware of the intervention received by study participants?	N
	Note: *The study was open-label; outcome assessors were likely aware of the treatment assignment.*	
4.4	If Y/PY/NI to 4.3: Could assessment of the outcome have been influenced by knowledge of intervention received?	PN
	Note: *Although assessors were unblinded, the primary outcomes (e.g., survival, recurrence on imaging) are objective and based on standard criteria, reducing the risk of detection bias.*	
4.5	If Y/PY/NI to 4.4: Is it likely that assessment of the outcome was influenced by knowledge of intervention received?	NA
**Risk-of-bias judgment**		Low
**5**	**Bias in selection of the reported result**	
5.1	Were the data that produced this result analysed in accordance with a prespecified analysis plan that was finalised before unblinded outcome data were available for analysis?	PY
	Note: *The trial was registered (NCT02539212), but the manuscript does not reference a predefined statistical analysis plan (SAP), and the registration number suggests a registration date (2015) after the start of the trial (2008). However, the registration of clinical trials was not that popular and accessible back in 2009. Thus, we thought the registration after the completion of the study didn't suggest an intentionally modified study protocol. So the analysis was possibly in line with a prespecified analysis, considering the relatively comprehensive design and report of this study.*	
Is the numerical result being assessed likely to have been selected, on the basis of the results, from:		
5.2	Multiple eligible outcome measurements (eg, scales, definitions, time points) within the outcome domain?	N
	Note: *The outcomes reported (technical effectiveness, local progression, survival) are standard and pre-specified in the abstract. There is no evidence of selective reporting from multiple definitions.*	
5.3	Multiple eligible analyses of the data?	N
	Note: *The statistical methods (log-rank test for survival) are standard and appropriate. There is no indication of data dredging.*	
**Risk-of-bias judgment**		Low
**Overall bias**		Low

Zhang2021

**Title:** Long-term Outcomes of Transcatheter Arterial Chemoembolization Combined With Radiofrequency Ablation as an Initial Treatment for Early-Stage Hepatocellular Carcinoma		
**Authors:** Yao Jun Zhang; Min Shan Chen; Yong Chen, *et al.*		**Year:** 2021
**Interventions:** Transcatheter arterial chemoembolization (TACE) + Radiofrequency ablation (RFA) vs. RFA		
**Registration:** NCT00554905		
**1**	**Bias arising from the randomisation process**	
1.1	Was the allocation sequence random?	Y
	Note: *The study is based on a previous phase 3 randomized clinical trial (NCT00554905), where participants were randomly assigned to the TACE-RFA or RFA group in a 1:1 ratio.*	
1.2	Was the allocation sequence concealed until participants were enrolled and assigned to interventions?	PY
	Note: *Although the study is derived from an RCT, the publication does not explicitly describe the method of allocation concealment. However, there is no evidence of selection bias.*	
1.3	Did baseline differences between intervention groups suggest a problem with the randomisation process?	N
	Note: *Baseline characteristics were well matched between the two groups, as shown in Table 1, indicating successful randomization.*	
**Risk-of-bias judgment**		Low
**2**	**Bias due to deviations from intended interventions**	
2.1	Were participants aware of their assigned intervention during the trial?	Y
	Note: *Due to the nature of the interventions (TACE + RFA vs. RFA alone), participants were likely aware of their treatment assignment.*	
2.2	Were carers and people delivering the interventions aware of participants' assigned intervention during the trial?	Y
	Note: *The clinicians performing TACE and RFA were necessarily aware of the treatment group.*	
2.3	If Y/PY/NI to 2.1 or 2.2: Were there deviations from the intended intervention that arose because of the trial context?	N
	Note: *There is no indication that deviations from the intended intervention occurred due to the trial context.*	
2.4	If Y/PY/NI to 2.3: Were these deviations likely to have affected the outcome?	NA
2.5	If Y/PY to 2.4: Were these deviations from intended intervention balanced between groups?	NA
2.6	Was an appropriate analysis used to estimate the effect of assignment to intervention?	Y
	Note: *The analysis was performed on an intention-to-treat basis, and Kaplan-Meier and Cox regression methods were appropriately used.*	
2.7	If N/PN/NI to 2.6: Was there potential for a substantial impact (on the result) of the failure to analyse participants in the group to which they were randomised?	NA
**Risk-of-bias judgment**		Low
**3**	**Bias due to missing outcome data**	
3.1	Were data for this outcome available for all, or nearly all, participants randomised?	Y
	Note: *Of the 189 randomised patients, only 6 (3.2%) were lost to follow-up (3 in each group), indicating very low attrition.*	
3.2	If N/PN/NI to 3.1: Is there evidence that the result was not biased by missing outcome data?	NA
3.3	If N/PN to 3.2: Could missingness in the outcome depend on its true value?	NA

Zhang2021 *(continued)*

3.4	If Y/PY/NI to 3.3: Is it likely that missingness in the outcome depended on its true value?	NA
**Risk-of-bias judgment**		Low
**4**	**Bias in measurement of the outcome**	
4.1	Was the method of measuring the outcome inappropriate?<br>***Note:*** *Overall survival and recurrence-free survival were assessed using standard clinical and imaging follow-up, which are appropriate and objective.*	N
4.2	Could measurement or ascertainment of the outcome have differed between intervention groups?<br>***Note:*** *The same follow-up protocols (imaging, blood tests, clinical evaluation) were applied to both groups.*	N
4.3	If N/PN/NI to 4.1 and 4.2: Were outcome assessors aware of the intervention received by study participants?<br>***Note:*** *The manuscript does not explicitly state whether outcome assessors (e.g., radiologists) were blinded. However, the objective nature of survival outcomes reduces the concern for bias.*	NI
4.4	If Y/PY/NI to 4.3: Could assessment of the outcome have been influenced by knowledge of intervention received?<br>***Note:*** *Although blinding was not mentioned, the outcomes (OS and RFS) are objective and unlikely to be influenced by knowledge of the intervention.*	PN
4.5	If Y/PY/NI to 4.4: Is it likely that assessment of the outcome was influenced by knowledge of intervention received?	NA
**Risk-of-bias judgment**		Low
**5**	**Bias in selection of the reported result**	
5.1	Were the data that produced this result analysed in accordance with a prespecified analysis plan that was finalised before unblinded outcome data were available for analysis?<br>***Note:*** *The trial was registered (NCT00554905), and the outcomes reported (OS and RFS) align with the registered protocol.*	Y
Is the numerical result being assessed likely to have been selected, on the basis of the results, from:		
5.2	Multiple eligible outcome measurements (eg, scales, definitions, time points) within the outcome domain?<br>***Note:*** *The primary outcomes (OS and RFS) were clearly pre-specified, and there is no evidence of selective reporting from multiple measurements.*	N
5.3	Multiple eligible analyses of the data?<br>***Note:*** *The statistical methods were pre-specified and consistently applied. There is no indication of data dredging or selective analysis.*	N
**Risk-of-bias judgment**		Low
**Overall bias**		Low

Zhang2022

**Title:** Novel irreversible electroporation ablation (Nano-knife) versus radiofrequency ablation for the treatment of solid liver tumors: a comparative, randomized, multicenter clinical study	
**Authors:** Xiaobo Zhang, Xiao Zhang, Xiaoyi Ding, *et al.*	**Year:** 2022
**Interventions:** Irreversible electroporation ablation (IRE) vs. Radiofrequency ablation (RFA)	
**Registration:** ChiCTR1800017516	

**1**	**Bias arising from the randomisation process**	
1.1	Was the allocation sequence random?	Y
	Note: *The authors used stratified permuted block randomization generated by a randomization specialist using SAS® software, which ensures a random allocation sequence.*	
1.2	Was the allocation sequence concealed until participants were enrolled and assigned to interventions?	PY
	Note: *Although sealed envelopes were used, the description does not explicitly confirm that the envelopes were opaque or sequentially numbered to ensure full concealment. However, there is no evidence of deliberate manipulation.*	
1.3	Did baseline differences between intervention groups suggest a problem with the randomisation process?	N
	Note: *Baseline demographic and clinical characteristics were well-balanced between the IRE and RFA groups, with no statistically significant differences.*	
**Risk-of-bias judgment**		Low
**2**	**Bias due to deviations from intended interventions**	
2.1	Were participants aware of their assigned intervention during the trial?	PY
	Note: *The study did not mention whether participants were blinded to the intervention. Given the nature of the procedures, blinding may not have been feasible.*	
2.2	Were carers and people delivering the interventions aware of participants' assigned intervention during the trial?	Y
	Note: *The surgeons and medical staff performing the ablations were necessarily aware of the treatment assignment due to the different equipment and techniques used.*	
2.3	If Y/PY/NI to 2.1 or 2.2: Were there deviations from the intended intervention that arose because of the trial context?	N
	Note: *There is no indication that deviations occurred due to the trial context. Both interventions were performed as per standard protocols.*	
2.4	If Y/PY/NI to 2.3: Were these deviations likely to have affected the outcome?	NA
2.5	If Y/PY to 2.4: Were these deviations from intended intervention balanced between groups?	NA
2.6	Was an appropriate analysis used to estimate the effect of assignment to intervention?	Y
	Note: *The analysis was performed on both per-protocol and full-analysis sets, and intention-to-treat principles were appropriately applied.*	
2.7	If N/PN/NI to 2.6: Was there potential for a substantial impact (on the result) of the failure to analyse participants in the group to which they were randomised?	NA
**Risk-of-bias judgment**		Low
**3**	**Bias due to missing outcome data**	
3.1	Were data for this outcome available for all, or nearly all, participants randomised?	Y
	Note: *Of the 156 randomised patients, 152 completed the trial, resulting in a low dropout rate (2.56%). Data availability was high for all primary and secondary outcomes.*	

Zhang2022 *(continued)*

3.2	If N/PN/NI to 3.1: Is there evidence that the result was not biased by missing outcome data?	NA
3.3	If N/PN to 3.2: Could missingness in the outcome depend on its true value?	NA
3.4	If Y/PY/NI to 3.3: Is it likely that missingness in the outcome depended on its true value?	NA
**Risk-of-bias judgment**		Low
**4**	**Bias in measurement of the outcome**	
4.1	Was the method of measuring the outcome inappropriate?	N
	Note: *Outcomes were assessed using enhanced CT or MRI, which are standard and objective imaging modalities for evaluating ablation success and recurrence.*	
4.2	Could measurement or ascertainment of the outcome have differed between intervention groups?	N
	Note: *The same imaging methods and criteria were applied to both groups.*	
4.3	If N/PN/NI to 4.1 and 4.2: Were outcome assessors aware of the intervention received by study participants?	Y
	Note: *Radiologists assessing the images were likely aware of the treatment group, as the ablation zones may appear different between IRE and RFA.*	
4.4	If Y/PY/NI to 4.3: Could assessment of the outcome have been influenced by knowledge of intervention received?	PN
	Note: *Although assessors were likely unblinded, the use of objective imaging criteria (presence or absence of enhancement) reduces the likelihood of bias.*	
4.5	If Y/PY/NI to 4.4: Is it likely that assessment of the outcome was influenced by knowledge of intervention received?	NA
**Risk-of-bias judgment**		Low
**5**	**Bias in selection of the reported result**	
5.1	Were the data that produced this result analysed in accordance with a prespecified analysis plan that was finalised before unblinded outcome data were available for analysis?	PY
	Note: *The trial was registered (ChiCTR1800017516), and the primary and secondary outcomes appear consistent with the registry. However, the published protocol or statistical analysis plan was not referenced to confirm full adherence.*	
Is the numerical result being assessed likely to have been selected, on the basis of the results, from:		
5.2	Multiple eligible outcome measurements (eg, scales, definitions, time points) within the outcome domain?	N
	Note: *There is no evidence that multiple definitions or time points were explored for the primary outcome. The success rate was clearly defined and measured at 1 week.*	
5.3	Multiple eligible analyses of the data?	N
	Note: *The statistical methods were pre-specified and consistently applied. There is no indication of alternative analyses being performed selectively.*	
**Risk-of-bias judgment**		Low
**Overall bias**		Low

Zhang2025

Title: Transarterial Chemoembolization with Radiofrequency Ablation versus Surgical Resection for Small Late-Recurrence Hepatocellular Carcinoma

Authors: Yaojun Zhang, Jinbin Chen, Zhongguo Zhou, *et al.* **Year:** 2025

Interventions: Transcatheter arterial chemoembolization (TACE) + Radiofrequency ablation (RFA) vs. Surgical resection (SR)

Registration: NCT01833286

**1**	**Bias arising from the randomisation process**	
1.1	Was the allocation sequence random?<br>***Note:*** *The study was a randomized clinical trial with patients assigned to TACE-RFA or SR groups in a 1:1 ratio using stratified randomization (by tumor size and number).*	Y
1.2	Was the allocation sequence concealed until participants were enrolled and assigned to interventions?<br>***Note:*** *Although the trial was randomized, the publication does not explicitly describe the method of allocation concealment. However, there is no evidence of selection bias.*	PY
1.3	Did baseline differences between intervention groups suggest a problem with the randomisation process?<br>***Note:*** *Baseline characteristics were well balanced between the two groups (Table 1), indicating successful randomization.*	N
**Risk-of-bias judgment**		Low
**2**	**Bias due to deviations from intended interventions**	
2.1	Were participants aware of their assigned intervention during the trial?<br>***Note:*** *Due to the nature of the interventions (TACE-RFA vs. surgical resection), participants were aware of their treatment assignment.*	Y
2.2	Were carers and people delivering the interventions aware of participants' assigned intervention during the trial?<br>***Note:*** *The clinicians performing TACE, RFA, or surgery were necessarily aware of the treatment group.*	Y
2.3	If Y/PY/NI to 2.1 or 2.2: Were there deviations from the intended intervention that arose because of the trial context?<br>***Note:*** *There is no indication that deviations from the intended intervention occurred due to the trial context.*	N
2.4	If Y/PY/NI to 2.3: Were these deviations likely to have affected the outcome?	NA
2.5	If Y/PY to 2.4: Were these deviations from intended intervention balanced between groups?	NA
2.6	Was an appropriate analysis used to estimate the effect of assignment to intervention?<br>***Note:*** *The analysis was performed on both intention-to-treat and per-protocol populations, and Kaplan-Meier and Cox regression methods were appropriately used.*	Y
2.7	If N/PN/NI to 2.6: Was there potential for a substantial impact (on the result) of the failure to analyse participants in the group to which they were randomised?	NA
**Risk-of-bias judgment**		Low
**3**	**Bias due to missing outcome data**	
3.1	Were data for this outcome available for all, or nearly all, participants randomised?	Y

	Note: *Of the 210 randomized patients, only a small number were excluded from the per-protocol analysis (12 patients), and outcome data were available for the vast majority. Loss to follow-up was minimal.*	
3.2	If N/PN/NI to 3.1: Is there evidence that the result was not biased by missing outcome data?	NA
3.3	If N/PN to 3.2: Could missingness in the outcome depend on its true value?	NA
3.4	If Y/PY/NI to 3.3: Is it likely that missingness in the outcome depended on its true value?	NA
**Risk-of-bias judgment**		Low
**4**	**Bias in measurement of the outcome**	
4.1	Was the method of measuring the outcome inappropriate?	N
	Note: *Overall survival and recurrence-free survival were assessed using standard clinical and imaging follow-up, which are objective and appropriate.*	
4.2	Could measurement or ascertainment of the outcome have differed between intervention groups?	N
	Note: *The same follow-up protocols (imaging, clinical evaluation) were applied to both groups.*	
4.3	If N/PN/NI to 4.1 and 4.2: Were outcome assessors aware of the intervention received by study participants?	NI
	Note: *The manuscript does not explicitly state whether outcome assessors (e.g., radiologists) were blinded. However, the objective nature of survival outcomes reduces the concern for bias.*	
4.4	If Y/PY/NI to 4.3: Could assessment of the outcome have been influenced by knowledge of intervention received?	PN
	Note: *Although blinding was not mentioned, the outcomes (OS and RFS) are objective and unlikely to be influenced by knowledge of the intervention.*	
4.5	If Y/PY/NI to 4.4: Is it likely that assessment of the outcome was influenced by knowledge of intervention received?	NA
**Risk-of-bias judgment**		Low
**5**	**Bias in selection of the reported result**	
5.1	Were the data that produced this result analysed in accordance with a prespecified analysis plan that was finalised before unblinded outcome data were available for analysis?	Y
	Note: *The trial was registered (NCT01833286), and the outcomes reported (OS and RFS) align with the registered protocol.*	
Is the numerical result being assessed likely to have been selected, on the basis of the results, from:		
5.2	Multiple eligible outcome measurements (eg, scales, definitions, time points) within the outcome domain?	N
	Note: *The primary and secondary outcomes were clearly pre-specified, and there is no evidence of selective reporting from multiple measurements.*	
5.3	Multiple eligible analyses of the data?	N
	Note: *The statistical methods were pre-specified and consistently applied. There is no indication of data dredging or selective analysis.*	
**Risk-of-bias judgment**		Low
**Overall bias**		Low

The list of included RCTs

1. Abdelaziz A, Elbaz T, Shousha HI, Mahmoud S, Ibrahim M, Abdelmaksoud A, et al. Efficacy and survival analysis of percutaneous radiofrequency versus microwave ablation for hepatocellular carcinoma: an Egyptian multidisciplinary clinic experience. *Surg Endosc.* 2014 Dec;**28**(12):3429–34.

2. Brunello F, Veltri A, Carucci P, Pagano E, Ciccone G, Moretto P, et al. Radiofrequency ablation versus ethanol injection for early hepatocellular carcinoma: a randomized controlled trial. *Scand J Gastroenterol* 2008;**43**:727-35. https://dx.doi.org/10.1080/00365520701885481

3. Bush DA, Volk M, Smith JC, Reeves ME, Sanghvi S, Slater JD, et al. Proton beam radiotherapy versus transarterial chemoembolization for hepatocellular carcinoma: Results of a randomized clinical trial. *Cancer.* 2023 Nov 15;**129**(22):3554–63.

4. Chen K, Chen G, Wang H, Li H, Xiao J, Duan X, et al. Increased survival in hepatocellular carcinoma with iodine-125 implantation plus radiofrequency ablation: a prospective randomized controlled trial. *J Hepatol.* 2014 Dec;**61**(6):1304–11.

5. Chen MS, Li JQ, Zheng Y, Guo RP, Liang HH, Zhang YQ, et al. A prospective randomized trial comparing percutaneous local ablative therapy and partial hepatectomy for small hepatocellular carcinoma. *Ann Surg.* 2006 Mar;**243**(3):321–8.

6. Chong CCN, Lee KF, Cheung SYS, Chu CCM, Fong AKW, Wong J, et al. Prospective double-blinded randomized controlled trial of Microwave versus RadioFrequency Ablation for hepatocellular carcinoma (McRFA trial). *HPB (Oxford).* 2020 Aug;**22**(8):1121–7.

7. Costanzo G, Tortora R, D'Adamo G, Luca M, Lampasi F, Addario L. Radiofrequency ablation versus laser ablation for the treatment of small hepatocellular carcinoma in cirrhosis: a randomized trial. *Journal of gastroenterology and hepatology.* 2015;**30**(3):559–565.

8. Fang C, Luo R, Zhang Y, Wang J, Feng K, Liu S, et al. Hepatectomy versus transcatheter arterial chemoembolization for resectable BCLC stage A/B hepatocellular carcinoma beyond Milan criteria: A randomized clinical trial. *Front Oncol.* 2023;**13**:1101162.

9. Fang Y, Chen W, Liang X, Li D, Lou H, Chen R, et al. Comparison of long-term effectiveness and complications of radiofrequency ablation with hepatectomy for small hepatocellular carcinoma. *J Gastroenterol Hepatol.* 2014 Jan;**29**(1):193–200.

10. Feng K, Yan J, Li X, Xia F, Ma K, Wang S, et al. A randomized controlled trial of radiofrequency ablation and surgical resection in the treatment of small hepatocellular carcinoma. *J Hepatol.* 2012 Oct;**57**(4):794–802.

11. Féray C, Campion L, Mathurin P, Archambreaud I, Mirabel X, Bronowicki JP, et al. TACE and conformal radiotherapy vs. TACE alone for hepatocellular carcinoma: A randomised controlled trial. *JHEP Rep.* 2023 Apr;**5**(4):100689.

12. Ferrari FS, Megliola A, Scorzelli A, Stella A, Vigni F, Drudi FM, et al. Treatment of small HCC through radiofrequency ablation and laser ablation. Comparison of techniques and long-term results. *Radiol Med* 2007;**112**:377-93. https://doi.org/10.1007/s11547-007-0148-2

13. Giorgio A, Di Sarno A, De Stefano G, Scognamiglio U, Farella N, Mariniello A, et al. Percutaneous radiofrequency ablation of hepatocellular carcinoma compared to percutaneous ethanol injection in treatment of cirrhotic patients: an Italian randomized controlled trial. *Anticancer Res.* 2011 Jun;**31**(6):2291–5.

14. Gjoreski A, Jovanoska I, Risteski F, Prgova Veljanova B, Nedelkovski D, Dimov V, et al. Single-center randomized trial comparing conventional chemoembolization versus doxorubicin-loaded polyethylene glycol microspheres for early- and intermediate-stage hepatocellular carcinoma. *Eur J Cancer Prev.* 2021 May 1;**30**(3):258–66.

15. Huang GT, Lee PH, Tsang YM, Lai MY, Yang PM, Hu RH, et al. Percutaneous ethanol injection versus surgical resection for the treatment of small hepatocellular carcinoma: a prospective study. *Ann Surg* 2005;**242**:36-42. https://doi.org/10.1097/01.sla.0000167925.90380.fe

16. Huang J, Yan L, Cheng Z, Wu H, Du L, Wang J, et al. A randomized trial comparing radiofrequency ablation and surgical resection for HCC conforming to the Milan criteria. *Ann Surg* 2010;**252**:903-12. https://dx.doi.org/10.1097/SLA.0b013e3181efc656

17. Kamal A, Elmoety AAA, Rostom YAM, Shater MS, Lashen SA. Percutaneous radiofrequency versus microwave ablation for management of hepatocellular carcinoma: a randomized controlled trial. *J Gastrointest Oncol.* 2019 Jun;**10**(3):562–71.

18. Koda M, Murawaki Y, Mitsuda A, Oyama K, Okamoto K, Idobe Y, et al. Combination therapy with transcatheter arterial chemoembolization and percutaneous ethanol injection compared with percutaneous ethanol injection alone for patients with small hepatocellular carcinoma: a randomized control study. *Cancer.* 2001 Sep 15;**92**(6):1516–24.

19. Lee HW, Lee JM, Yoon JH, Kim YJ, Park JW, Park SJ, et al. A prospective randomized study comparing radiofrequency ablation and hepatic resection for hepatocellular carcinoma. *Ann Surg Treat Res.* 2018 Feb;**94**(2):74–82.

20. Lencioni RA, Allgaier HP, Cioni D, Olschewski M, Deibert P, Crocetti L, et al. Small hepatocellular carcinoma in cirrhosis: randomized comparison of radio-frequency thermal ablation versus percutaneous ethanol injection. *Radiology* 2003;**228**:235-40.

21. Lin SM, Lin CJ, Lin CC, Hsu CW, Chen YC. Randomised controlled trial comparing percutaneous radiofrequency thermal ablation, percutaneous ethanol injection, and percutaneous acetic acid injection to treat hepatocellular carcinoma of 3 cm or less. *Gut* 2005;**54**:1151-6. http://dx.doi.org/10.1136/gut.2004.045203

22. Liu H, Wang ZG, Fu SY, Li AJ, Pan ZY, Zhou WP, et al. Randomized clinical trial of chemoembolization plus radiofrequency ablation versus partial hepatectomy for hepatocellular carcinoma within the Milan criteria. *Br J Surg* 2016;**103**:348-56. https://dx.doi.org/10.1002/bjs.10061

23. Mizuki A, Tatemichi M, Tsukada N, Nagamatsu R, Kawaguchi M, Itoshima T, et al. Addition of transcatheter arterial chemoembolization decreased local recurrence but had no survival benefit to percutaneous ethanol injection therapy for patients with small hepatocellular carcinoma: a multicenter randomized control study. *Oncol Lett* 2010;**1**:855-9. https://doi.org/10.3892/ol_00000151

24. Ng KKC, Chok KSH, Chan ACY, Cheung TT, Wong TCL, Fung JYY, et al. Randomized clinical trial of hepatic resection versus radiofrequency ablation for early-stage hepatocellular carcinoma. *Br J Surg.* 2017 Dec;**104**(13):1775–84.

25. Orlacchio A, Bolacchi F, Chegai F, Bergamini A, Costanzo E, Del Giudice C, et al. Comparative evaluation of percutaneous laser and radiofrequency ablation in patients with HCC smaller than 4 cm. *Radiol Med* 2014;**119**:298-308. https://dx.doi.org/10.1007/s11547-013-0339-y

26. Park SJ, Cho EJ, Lee JH, Yu SJ, Kim YJ, Yoon JH, et al. Switching Monopolar No-Touch Radiofrequency Ablation Using Octopus Electrodes for Small Hepatocellular Carcinoma: A Randomized Clinical Trial. *Liver Cancer.* 2021 Feb;**10**(1):72–81.

27. Shibata T, Iimuro Y, Yamamoto Y, Maetani Y, Ametani F, Itoh K, et al. Small hepatocellular carcinoma: comparison of radio-frequency ablation and percutaneous microwave coagulation therapy. *Radiology* 2002;**223**:331-7. https://doi.org/10.1148/radiol.2232010775

28. Shibata T, Isoda H, Hirokawa Y, Arizono S, Shimada K, Togashi K. Small hepatocellular carcinoma: is radiofrequency ablation combined with transcatheter arterial chemoembolization more effective than radiofrequency ablation alone for treatment? *Radiology.* 2009 Sep;**252**(3):905–13.

29. Song J, Cao L, Ma K, Li J, Wang X, Chen J, et al. Laparoscopic liver resection versus radiofrequency ablation for small hepatocellular carcinoma: randomized clinical trial. *Br J Surg.* 2024 Apr 3;**111**(4):znae099.

30. Sugimoto K, Imajo K, Kuroda H, Murohisa G, Shiozawa K, Sakamaki K, et al. Microwave ablation vs. single-needle radiofrequency ablation for the treatment of HCC up to 4 cm: A randomized-controlled trial. *JHEP Rep.* 2025 Jan;**7**(1):101269.

31. Suh YS, Choi JW, Yoon JH, Lee DH, Kim YJ, Lee JH, et al. No-Touch vs. Conventional Radiofrequency Ablation Using Twin Internally Cooled Wet Electrodes for Small Hepatocellular Carcinomas: A Randomized Prospective Comparative Study. *Korean J Radiol.* 2021 Dec;**22**(12):1974–84.

32. Takayama T, Hasegawa K, Izumi N, Kudo M, Shimada M, Yamanaka N, et al. Surgery versus Radiofrequency Ablation for Small Hepatocellular Carcinoma: A Randomized Controlled Trial (SURF Trial). *Liver Cancer.* 2022 Jun;**11**(3):209–18.

33. Vietti Violi N, Duran R, Guiu B, Cercueil JP, Aubé C, Digklia A, et al. Efficacy of microwave ablation versus radiofrequency ablation for the treatment of hepatocellular carcinoma in patients with chronic liver disease: a randomised controlled phase 2 trial. *Lancet Gastroenterol Hepatol.* 2018 May;**3**(5):317–25.

34. Vogl TJ, Martin SS, Gruber-Rouh T, Booz C, Koch V, Nour-Eldin NEA, et al. Comparison of Microwave and Radiofrequency Ablation for the Treatment of Small- and Medium-Sized Hepatocellular Carcinomas in a Prospective Randomized Trial. *Rofo.* 2024 May;**196**(5):482–90.

35. Wang C, Wang H, Yang W, Hu K, Xie H, Hu KQ, et al. Multicenter randomized controlled trial of percutaneous cryoablation versus radiofrequency ablation in hepatocellular carcinoma. *Hepatology.* 2015 May;**61**(5):1579–90.

36. Wei X, Jiang Y, Feng S, Lu C, Huo L, Zhou B, et al. Neoadjuvant intensity modulated radiotherapy for a single and small ($\leq$ 5 cm) hepatitis B virus-related hepatocellular carcinoma predicted to have high risks of microvascular invasion: a randomized clinical trial. *Int J Surg.* 2023 Oct 1;**109**(10):3052–60.

37. Xi M, Yang Z, Hu L, Fu Y, Hu D, Zhou Z, et al. Radiofrequency Ablation Versus Stereotactic Body Radiotherapy for Recurrent Small Hepatocellular Carcinoma: A Randomized, Open-Label, Controlled Trial. *J Clin Oncol.* 2024 Dec 18;JCO2401532.

38. Yu J, Yu XL, Han ZY, Cheng ZG, Liu FY, Zhai HY, et al. Percutaneous cooled-probe microwave versus radiofrequency ablation in early-stage hepatocellular carcinoma: a phase III randomised controlled trial. *Gut.* 2017 Jun;**66**(6):1172–3.

39. Zhang YJ, Chen MS, Chen Y, Lau WY, Peng Z. Long-term Outcomes of Transcatheter Arterial Chemoembolization Combined With Radiofrequency Ablation as an Initial Treatment for Early-Stage Hepatocellular Carcinoma. *JAMA Netw Open.* 2021 Sep 1;**4**(9):e2126992.

40. Zhang X, Zhang X, Ding X, Wang Z, Fan Y, Chen G, et al. Novel irreversible electroporation ablation (Nano-knife) versus radiofrequency ablation for the treatment of solid liver tumors: a comparative, randomized, multicenter clinical study. *Front Oncol.* 2022;**12**:945123.

41. Zhang YJ, Chen J, Zhou Z, Hu D, Wang J, Pan Y, et al. Transarterial Chemoembolization with Radiofrequency Ablation versus Surgical Resection for Small Late-Recurrence Hepatocellular Carcinoma. *Radiology.* 2025 Feb;**314**(2):e241096.
